# Performance of algorithms that reconstruct missing transverse momentum in $\sqrt{s}$= 8 TeV proton–proton collisions in the ATLAS detector

**DOI:** 10.1140/epjc/s10052-017-4780-2

**Published:** 2017-04-13

**Authors:** G. Aad, B. Abbott, J. Abdallah, O. Abdinov, B. Abeloos, R. Aben, M. Abolins, O. S. AbouZeid, H. Abramowicz, H. Abreu, R. Abreu, Y. Abulaiti, B. S. Acharya, L. Adamczyk, D. L. Adams, J. Adelman, S. Adomeit, T. Adye, A. A. Affolder, T. Agatonovic-Jovin, J. Agricola, J. A. Aguilar-Saavedra, S. P. Ahlen, F. Ahmadov, G. Aielli, H. Akerstedt, T. P. A. Åkesson, A. V. Akimov, G. L. Alberghi, J. Albert, S. Albrand, M. J. Alconada Verzini, M. Aleksa, I. N. Aleksandrov, C. Alexa, G. Alexander, T. Alexopoulos, M. Alhroob, G. Alimonti, L. Alio, J. Alison, S. P. Alkire, B. M. M. Allbrooke, B. W. Allen, P. P. Allport, A. Aloisio, A. Alonso, F. Alonso, C. Alpigiani, B. Alvarez Gonzalez, D. Álvarez Piqueras, M. G. Alviggi, B. T. Amadio, K. Amako, Y. Amaral Coutinho, C. Amelung, D. Amidei, S. P. Amor Dos Santos, A. Amorim, S. Amoroso, N. Amram, G. Amundsen, C. Anastopoulos, L. S. Ancu, N. Andari, T. Andeen, C. F. Anders, G. Anders, J. K. Anders, K. J. Anderson, A. Andreazza, V. Andrei, S. Angelidakis, I. Angelozzi, P. Anger, A. Angerami, F. Anghinolfi, A. V. Anisenkov, N. Anjos, A. Annovi, M. Antonelli, A. Antonov, J. Antos, F. Anulli, M. Aoki, L. Aperio Bella, G. Arabidze, Y. Arai, J. P. Araque, A. T. H. Arce, F. A. Arduh, J-F. Arguin, S. Argyropoulos, M. Arik, A. J. Armbruster, O. Arnaez, H. Arnold, M. Arratia, O. Arslan, A. Artamonov, G. Artoni, S. Artz, S. Asai, N. Asbah, A. Ashkenazi, B. Åsman, L. Asquith, K. Assamagan, R. Astalos, M. Atkinson, N. B. Atlay, K. Augsten, G. Avolio, B. Axen, M. K. Ayoub, G. Azuelos, M. A. Baak, A. E. Baas, M. J. Baca, H. Bachacou, K. Bachas, M. Backes, M. Backhaus, P. Bagiacchi, P. Bagnaia, Y. Bai, J. T. Baines, O. K. Baker, E. M. Baldin, P. Balek, T. Balestri, F. Balli, W. K. Balunas, E. Banas, Sw. Banerjee, A. A. E. Bannoura, L. Barak, E. L. Barberio, D. Barberis, M. Barbero, T. Barillari, T. Barklow, N. Barlow, S. L. Barnes, B. M. Barnett, R. M. Barnett, Z. Barnovska, A. Baroncelli, G. Barone, A. J. Barr, L. Barranco Navarro, F. Barreiro, J. Barreiro Guimarães da Costa, R. Bartoldus, A. E. Barton, P. Bartos, A. Basalaev, A. Bassalat, A. Basye, R. L. Bates, S. J. Batista, J. R. Batley, M. Battaglia, M. Bauce, F. Bauer, H. S. Bawa, J. B. Beacham, M. D. Beattie, T. Beau, P. H. Beauchemin, R. Beccherle, P. Bechtle, H. P. Beck, K. Becker, M. Becker, M. Beckingham, C. Becot, A. J. Beddall, A. Beddall, V. A. Bednyakov, M. Bedognetti, C. P. Bee, L. J. Beemster, T. A. Beermann, M. Begel, J. K. Behr, C. Belanger-Champagne, G. Bella, L. Bellagamba, A. Bellerive, M. Bellomo, K. Belotskiy, O. Beltramello, O. Benary, D. Benchekroun, M. Bender, K. Bendtz, N. Benekos, Y. Benhammou, E. Benhar Noccioli, J. A. Benitez Garcia, D. P. Benjamin, J. R. Bensinger, S. Bentvelsen, L. Beresford, M. Beretta, D. Berge, E. Bergeaas Kuutmann, N. Berger, F. Berghaus, J. Beringer, C. Bernard, N. R. Bernard, C. Bernius, F. U. Bernlochner, T. Berry, P. Berta, C. Bertella, G. Bertoli, F. Bertolucci, C. Bertsche, D. Bertsche, G. J. Besjes, O. Bessidskaia Bylund, M. Bessner, N. Besson, C. Betancourt, S. Bethke, A. J. Bevan, W. Bhimji, R. M. Bianchi, L. Bianchini, M. Bianco, O. Biebel, D. Biedermann, N. V. Biesuz, M. Biglietti, J. Bilbao De Mendizabal, H. Bilokon, M. Bindi, S. Binet, A. Bingul, C. Bini, S. Biondi, D. M. Bjergaard, C. W. Black, J. E. Black, K. M. Black, D. Blackburn, R. E. Blair, J. -B. Blanchard, J. E. Blanco, T. Blazek, I. Bloch, C. Blocker, W. Blum, U. Blumenschein, S. Blunier, G. J. Bobbink, V. S. Bobrovnikov, S. S. Bocchetta, A. Bocci, C. Bock, M. Boehler, D. Boerner, J. A. Bogaerts, D. Bogavac, A. G. Bogdanchikov, C. Bohm, V. Boisvert, T. Bold, V. Boldea, A. S. Boldyrev, M. Bomben, M. Bona, M. Boonekamp, A. Borisov, G. Borissov, J. Bortfeldt, V. Bortolotto, K. Bos, D. Boscherini, M. Bosman, J. Boudreau, J. Bouffard, E. V. Bouhova-Thacker, D. Boumediene, C. Bourdarios, N. Bousson, S. K. Boutle, A. Boveia, J. Boyd, I. R. Boyko, J. Bracinik, A. Brandt, G. Brandt, O. Brandt, U. Bratzler, B. Brau, J. E. Brau, H. M. Braun, W. D. Breaden Madden, K. Brendlinger, A. J. Brennan, L. Brenner, R. Brenner, S. Bressler, T. M. Bristow, D. Britton, D. Britzger, F. M. Brochu, I. Brock, R. Brock, G. Brooijmans, T. Brooks, W. K. Brooks, J. Brosamer, E. Brost, P. A. Bruckman de Renstrom, D. Bruncko, R. Bruneliere, A. Bruni, G. Bruni, BH Brunt, M. Bruschi, N. Bruscino, P. Bryant, L. Bryngemark, T. Buanes, Q. Buat, P. Buchholz, A. G. Buckley, I. A. Budagov, F. Buehrer, L. Bugge, M. K. Bugge, O. Bulekov, D. Bullock, H. Burckhart, S. Burdin, C. D. Burgard, B. Burghgrave, S. Burke, I. Burmeister, E. Busato, D. Büscher, V. Büscher, P. Bussey, J. M. Butler, A. I. Butt, C. M. Buttar, J. M. Butterworth, P. Butti, W. Buttinger, A. Buzatu, A. R. Buzykaev, S. Cabrera Urbán, D. Caforio, V. M. Cairo, O. Cakir, N. Calace, P. Calafiura, A. Calandri, G. Calderini, P. Calfayan, L. P. Caloba, D. Calvet, S. Calvet, T. P. Calvet, R. Camacho Toro, S. Camarda, P. Camarri, D. Cameron, R. Caminal Armadans, C. Camincher, S. Campana, M. Campanelli, A. Campoverde, V. Canale, A. Canepa, M. Cano Bret, J. Cantero, R. Cantrill, T. Cao, M. D. M. Capeans Garrido, I. Caprini, M. Caprini, M. Capua, R. Caputo, R. M. Carbone, R. Cardarelli, F. Cardillo, I. Carli, T. Carli, G. Carlino, L. Carminati, S. Caron, E. Carquin, G. D. Carrillo-Montoya, J. R. Carter, J. Carvalho, D. Casadei, M. P. Casado, M. Casolino, D. W. Casper, E. Castaneda-Miranda, A. Castelli, V. Castillo Gimenez, N. F. Castro, A. Catinaccio, J. R. Catmore, A. Cattai, J. Caudron, V. Cavaliere, D. Cavalli, M. Cavalli-Sforza, V. Cavasinni, F. Ceradini, L. Cerda Alberich, B. C. Cerio, A. S. Cerqueira, A. Cerri, L. Cerrito, F. Cerutti, M. Cerv, A. Cervelli, S. A. Cetin, A. Chafaq, D. Chakraborty, Y. L. Chan, P. Chang, J. D. Chapman, D. G. Charlton, C. C. Chau, C. A. Chavez Barajas, S. Che, S. Cheatham, A. Chegwidden, S. Chekanov, S. V. Chekulaev, G. A. Chelkov, M. A. Chelstowska, C. Chen, H. Chen, K. Chen, S. Chen, S. Chen, X. Chen, Y. Chen, H. C. Cheng, Y. Cheng, A. Cheplakov, E. Cheremushkina, R. Cherkaoui El Moursli, V. Chernyatin, E. Cheu, L. Chevalier, V. Chiarella, G. Chiarelli, G. Chiodini, A. S. Chisholm, R. T. Chislett, A. Chitan, M. V. Chizhov, K. Choi, S. Chouridou, B. K. B. Chow, V. Christodoulou, D. Chromek-Burckhart, J. Chudoba, A. J. Chuinard, J. J. Chwastowski, L. Chytka, G. Ciapetti, A. K. Ciftci, D. Cinca, V. Cindro, I. A. Cioara, A. Ciocio, F. Cirotto, Z. H. Citron, M. Ciubancan, A. Clark, B. L. Clark, P. J. Clark, R. N. Clarke, C. Clement, Y. Coadou, M. Cobal, A. Coccaro, J. Cochran, L. Coffey, L. Colasurdo, B. Cole, S. Cole, A. P. Colijn, J. Collot, T. Colombo, G. Compostella, P. Conde Muiño, E. Coniavitis, S. H. Connell, I. A. Connelly, V. Consorti, S. Constantinescu, C. Conta, G. Conti, F. Conventi, M. Cooke, B. D. Cooper, A. M. Cooper-Sarkar, T. Cornelissen, M. Corradi, F. Corriveau, A. Corso-Radu, A. Cortes-Gonzalez, G. Cortiana, G. Costa, M. J. Costa, D. Costanzo, G. Cottin, G. Cowan, B. E. Cox, K. Cranmer, S. J. Crawley, G. Cree, S. Crépé-Renaudin, F. Crescioli, W. A. Cribbs, M. Crispin Ortuzar, M. Cristinziani, V. Croft, G. Crosetti, T. Cuhadar Donszelmann, J. Cummings, M. Curatolo, J. Cúth, C. Cuthbert, H. Czirr, P. Czodrowski, S. D’Auria, M. D’Onofrio, M. J. Da Cunha Sargedas De Sousa, C. Da Via, W. Dabrowski, A. Dafinca, T. Dai, O. Dale, F. Dallaire, C. Dallapiccola, M. Dam, J. R. Dandoy, N. P. Dang, A. C. Daniells, M. Danninger, M. Dano Hoffmann, V. Dao, G. Darbo, S. Darmora, J. Dassoulas, A. Dattagupta, W. Davey, C. David, T. Davidek, E. Davies, M. Davies, P. Davison, Y. Davygora, E. Dawe, I. Dawson, R. K. Daya-Ishmukhametova, K. De, R. de Asmundis, A. De Benedetti, S. De Castro, S. De Cecco, N. De Groot, P. de Jong, H. De la Torre, F. De Lorenzi, D. De Pedis, A. De Salvo, U. De Sanctis, A. De Santo, J. B. De Vivie De Regie, W. J. Dearnaley, R. Debbe, C. Debenedetti, D. V. Dedovich, I. Deigaard, J. Del Peso, T. Del Prete, D. Delgove, F. Deliot, C. M. Delitzsch, M. Deliyergiyev, A. Dell’Acqua, L. Dell’Asta, M. Dell’Orso, M. Della Pietra, D. della Volpe, M. Delmastro, P. A. Delsart, C. Deluca, D. A. DeMarco, S. Demers, M. Demichev, A. Demilly, S. P. Denisov, D. Denysiuk, D. Derendarz, J. E. Derkaoui, F. Derue, P. Dervan, K. Desch, C. Deterre, K. Dette, P. O. Deviveiros, A. Dewhurst, S. Dhaliwal, A. Di Ciaccio, L. Di Ciaccio, C. Di Donato, A. Di Girolamo, B. Di Girolamo, B. Di Micco, R. Di Nardo, A. Di Simone, R. Di Sipio, D. Di Valentino, C. Diaconu, M. Diamond, F. A. Dias, M. A. Diaz, E. B. Diehl, J. Dietrich, S. Diglio, A. Dimitrievska, J. Dingfelder, P. Dita, S. Dita, F. Dittus, F. Djama, T. Djobava, J. I. Djuvsland, M. A. B. do Vale, D. Dobos, M. Dobre, C. Doglioni, T. Dohmae, J. Dolejsi, Z. Dolezal, B. A. Dolgoshein, M. Donadelli, S. Donati, P. Dondero, J. Donini, J. Dopke, A. Doria, M. T. Dova, A. T. Doyle, E. Drechsler, M. Dris, Y. Du, J. Duarte-Campderros, E. Dubreuil, E. Duchovni, G. Duckeck, O. A. Ducu, D. Duda, A. Dudarev, L. Duflot, L. Duguid, M. Dührssen, M. Dunford, H. Duran Yildiz, M. Düren, A. Durglishvili, D. Duschinger, B. Dutta, M. Dyndal, C. Eckardt, K. M. Ecker, R. C. Edgar, W. Edson, N. C. Edwards, T. Eifert, G. Eigen, K. Einsweiler, T. Ekelof, M. El Kacimi, V. Ellajosyula, M. Ellert, S. Elles, F. Ellinghaus, A. A. Elliot, N. Ellis, J. Elmsheuser, M. Elsing, D. Emeliyanov, Y. Enari, O. C. Endner, M. Endo, J. S. Ennis, J. Erdmann, A. Ereditato, G. Ernis, J. Ernst, M. Ernst, S. Errede, E. Ertel, M. Escalier, H. Esch, C. Escobar, B. Esposito, A. I. Etienvre, E. Etzion, H. Evans, A. Ezhilov, L. Fabbri, G. Facini, R. M. Fakhrutdinov, S. Falciano, R. J. Falla, J. Faltova, Y. Fang, M. Fanti, A. Farbin, A. Farilla, C. Farina, T. Farooque, S. Farrell, S. M. Farrington, P. Farthouat, F. Fassi, P. Fassnacht, D. Fassouliotis, M. Faucci Giannelli, A. Favareto, L. Fayard, O. L. Fedin, W. Fedorko, S. Feigl, L. Feligioni, C. Feng, E. J. Feng, H. Feng, A. B. Fenyuk, L. Feremenga, P. Fernandez Martinez, S. Fernandez Perez, J. Ferrando, A. Ferrari, P. Ferrari, R. Ferrari, D. E. Ferreira de Lima, A. Ferrer, D. Ferrere, C. Ferretti, A. Ferretto Parodi, F. Fiedler, A. Filipčič, M. Filipuzzi, F. Filthaut, M. Fincke-Keeler, K. D. Finelli, M. C. N. Fiolhais, L. Fiorini, A. Firan, A. Fischer, C. Fischer, J. Fischer, W. C. Fisher, N. Flaschel, I. Fleck, P. Fleischmann, G. T. Fletcher, G. Fletcher, R. R. M. Fletcher, T. Flick, A. Floderus, L. R. Flores Castillo, M. J. Flowerdew, G. T. Forcolin, A. Formica, A. Forti, D. Fournier, H. Fox, S. Fracchia, P. Francavilla, M. Franchini, D. Francis, L. Franconi, M. Franklin, M. Frate, M. Fraternali, D. Freeborn, S. M. Fressard-Batraneanu, F. Friedrich, D. Froidevaux, J. A. Frost, C. Fukunaga, E. Fullana Torregrosa, T. Fusayasu, J. Fuster, C. Gabaldon, O. Gabizon, A. Gabrielli, A. Gabrielli, G. P. Gach, S. Gadatsch, S. Gadomski, G. Gagliardi, P. Gagnon, C. Galea, B. Galhardo, E. J. Gallas, B. J. Gallop, P. Gallus, G. Galster, K. K. Gan, J. Gao, Y. Gao, Y. S. Gao, F. M. Garay Walls, C. García, J. E. García Navarro, M. Garcia-Sciveres, R. W. Gardner, N. Garelli, V. Garonne, C. Gatti, A. Gaudiello, G. Gaudio, B. Gaur, L. Gauthier, I. L. Gavrilenko, C. Gay, G. Gaycken, E. N. Gazis, Z. Gecse, C. N. P. Gee, Ch. Geich-Gimbel, M. P. Geisler, C. Gemme, M. H. Genest, C. Geng, S. Gentile, S. George, D. Gerbaudo, A. Gershon, S. Ghasemi, H. Ghazlane, B. Giacobbe, S. Giagu, P. Giannetti, B. Gibbard, S. M. Gibson, M. Gignac, M. Gilchriese, T. P. S. Gillam, D. Gillberg, G. Gilles, D. M. Gingrich, N. Giokaris, M. P. Giordani, F. M. Giorgi, F. M. Giorgi, P. F. Giraud, P. Giromini, D. Giugni, C. Giuliani, M. Giulini, B. K. Gjelsten, S. Gkaitatzis, I. Gkialas, E. L. Gkougkousis, L. K. Gladilin, C. Glasman, J. Glatzer, P. C. F. Glaysher, A. Glazov, M. Goblirsch-Kolb, J. R. Goddard, J. Godlewski, S. Goldfarb, T. Golling, D. Golubkov, A. Gomes, R. Gonçalo, J. Goncalves Pinto Firmino Da Costa, L. Gonella, S. González de la Hoz, G. Gonzalez Parra, S. Gonzalez-Sevilla, L. Goossens, P. A. Gorbounov, H. A. Gordon, I. Gorelov, B. Gorini, E. Gorini, A. Gorišek, E. Gornicki, A. T. Goshaw, C. Gössling, M. I. Gostkin, C. R. Goudet, D. Goujdami, A. G. Goussiou, N. Govender, E. Gozani, L. Graber, I. Grabowska-Bold, P. O. J. Gradin, P. Grafström, J. Gramling, E. Gramstad, S. Grancagnolo, V. Gratchev, H. M. Gray, E. Graziani, Z. D. Greenwood, C. Grefe, K. Gregersen, I. M. Gregor, P. Grenier, K. Grevtsov, J. Griffiths, A. A. Grillo, K. Grimm, S. Grinstein, Ph. Gris, J.-F. Grivaz, S. Groh, J. P. Grohs, E. Gross, J. Grosse-Knetter, G. C. Grossi, Z. J. Grout, L. Guan, J. Guenther, F. Guescini, D. Guest, O. Gueta, E. Guido, T. Guillemin, S. Guindon, U. Gul, C. Gumpert, J. Guo, Y. Guo, S. Gupta, G. Gustavino, P. Gutierrez, N. G. Gutierrez Ortiz, C. Gutschow, C. Guyot, C. Gwenlan, C. B. Gwilliam, A. Haas, C. Haber, H. K. Hadavand, N. Haddad, A. Hadef, P. Haefner, S. Hageböck, Z. Hajduk, H. Hakobyan, M. Haleem, J. Haley, D. Hall, G. Halladjian, G. D. Hallewell, K. Hamacher, P. Hamal, K. Hamano, A. Hamilton, G. N. Hamity, P. G. Hamnett, L. Han, K. Hanagaki, K. Hanawa, M. Hance, B. Haney, P. Hanke, R. Hanna, J. B. Hansen, J. D. Hansen, M. C. Hansen, P. H. Hansen, K. Hara, A. S. Hard, T. Harenberg, F. Hariri, S. Harkusha, R. D. Harrington, P. F. Harrison, F. Hartjes, M. Hasegawa, Y. Hasegawa, A. Hasib, S. Hassani, S. Haug, R. Hauser, L. Hauswald, M. Havranek, C. M. Hawkes, R. J. Hawkings, A. D. Hawkins, T. Hayashi, D. Hayden, C. P. Hays, J. M. Hays, H. S. Hayward, S. J. Haywood, S. J. Head, T. Heck, V. Hedberg, L. Heelan, S. Heim, T. Heim, B. Heinemann, L. Heinrich, J. Hejbal, L. Helary, S. Hellman, C. Helsens, J. Henderson, R. C. W. Henderson, Y. Heng, S. Henkelmann, A. M. Henriques Correia, S. Henrot-Versille, G. H. Herbert, Y. Hernández Jiménez, G. Herten, R. Hertenberger, L. Hervas, G. G. Hesketh, N. P. Hessey, J. W. Hetherly, R. Hickling, E. Higón-Rodriguez, E. Hill, J. C. Hill, K. H. Hiller, S. J. Hillier, I. Hinchliffe, E. Hines, R. R. Hinman, M. Hirose, D. Hirschbuehl, J. Hobbs, N. Hod, M. C. Hodgkinson, P. Hodgson, A. Hoecker, M. R. Hoeferkamp, F. Hoenig, M. Hohlfeld, D. Hohn, T. R. Holmes, M. Homann, T. M. Hong, B. H. Hooberman, W. H. Hopkins, Y. Horii, A. J. Horton, J-Y. Hostachy, S. Hou, A. Hoummada, J. Howard, J. Howarth, M. Hrabovsky, I. Hristova, J. Hrivnac, T. Hryn’ova, A. Hrynevich, C. Hsu, P. J. Hsu, S.-C. Hsu, D. Hu, Q. Hu, Y. Huang, Z. Hubacek, F. Hubaut, F. Huegging, T. B. Huffman, E. W. Hughes, G. Hughes, M. Huhtinen, T. A. Hülsing, N. Huseynov, J. Huston, J. Huth, G. Iacobucci, G. Iakovidis, I. Ibragimov, L. Iconomidou-Fayard, E. Ideal, Z. Idrissi, P. Iengo, O. Igonkina, T. Iizawa, Y. Ikegami, M. Ikeno, Y. Ilchenko, D. Iliadis, N. Ilic, T. Ince, G. Introzzi, P. Ioannou, M. Iodice, K. Iordanidou, V. Ippolito, A. Irles Quiles, C. Isaksson, M. Ishino, M. Ishitsuka, R. Ishmukhametov, C. Issever, S. Istin, J. M. Iturbe Ponce, R. Iuppa, J. Ivarsson, W. Iwanski, H. Iwasaki, J. M. Izen, V. Izzo, S. Jabbar, B. Jackson, M. Jackson, P. Jackson, V. Jain, K. B. Jakobi, K. Jakobs, S. Jakobsen, T. Jakoubek, D. O. Jamin, D. K. Jana, E. Jansen, R. Jansky, J. Janssen, M. Janus, G. Jarlskog, N. Javadov, T. Javůrek, F. Jeanneau, L. Jeanty, J. Jejelava, G. -Y. Jeng, D. Jennens, P. Jenni, J. Jentzsch, C. Jeske, S. Jézéquel, H. Ji, J. Jia, H. Jiang, Y. Jiang, S. Jiggins, J. Jimenez Pena, S. Jin, A. Jinaru, O. Jinnouchi, P. Johansson, K. A. Johns, W. J. Johnson, K. Jon-And, G. Jones, R. W. L. Jones, S. Jones, T. J. Jones, J. Jongmanns, P. M. Jorge, J. Jovicevic, X. Ju, A. Juste Rozas, M. K. Köhler, M. Kaci, A. Kaczmarska, M. Kado, H. Kagan, M. Kagan, S. J. Kahn, E. Kajomovitz, C. W. Kalderon, A. Kaluza, S. Kama, A. Kamenshchikov, N. Kanaya, S. Kaneti, V. A. Kantserov, J. Kanzaki, B. Kaplan, L. S. Kaplan, A. Kapliy, D. Kar, K. Karakostas, A. Karamaoun, N. Karastathis, M. J. Kareem, E. Karentzos, M. Karnevskiy, S. N. Karpov, Z. M. Karpova, K. Karthik, V. Kartvelishvili, A. N. Karyukhin, K. Kasahara, L. Kashif, R. D. Kass, A. Kastanas, Y. Kataoka, C. Kato, A. Katre, J. Katzy, K. Kawagoe, T. Kawamoto, G. Kawamura, S. Kazama, V. F. Kazanin, R. Keeler, R. Kehoe, J. S. Keller, J. J. Kempster, K Kentaro, H. Keoshkerian, O. Kepka, B. P. Kerševan, S. Kersten, R. A. Keyes, F. Khalil-zada, H. Khandanyan, A. Khanov, A. G. Kharlamov, T. J. Khoo, V. Khovanskiy, E. Khramov, J. Khubua, S. Kido, H. Y. Kim, S. H. Kim, Y. K. Kim, N. Kimura, O. M. Kind, B. T. King, M. King, S. B. King, J. Kirk, A. E. Kiryunin, T. Kishimoto, D. Kisielewska, F. Kiss, K. Kiuchi, O. Kivernyk, E. Kladiva, M. H. Klein, M. Klein, U. Klein, K. Kleinknecht, P. Klimek, A. Klimentov, R. Klingenberg, J. A. Klinger, T. Klioutchnikova, E. -E. Kluge, P. Kluit, S. Kluth, J. Knapik, E. Kneringer, E. B. F. G. Knoops, A. Knue, A. Kobayashi, D. Kobayashi, T. Kobayashi, M. Kobel, M. Kocian, P. Kodys, T. Koffas, E. Koffeman, L. A. Kogan, S. Kohlmann, T. Koi, H. Kolanoski, M. Kolb, I. Koletsou, A. A. Komar, Y. Komori, T. Kondo, N. Kondrashova, K. Köneke, A. C. König, T. Kono, R. Konoplich, N. Konstantinidis, R. Kopeliansky, S. Koperny, L. Köpke, A. K. Kopp, K. Korcyl, K. Kordas, A. Korn, A. A. Korol, I. Korolkov, E. V. Korolkova, O. Kortner, S. Kortner, T. Kosek, V. V. Kostyukhin, V. M. Kotov, A. Kotwal, A. Kourkoumeli-Charalampidi, C. Kourkoumelis, V. Kouskoura, A. Koutsman, R. Kowalewski, T. Z. Kowalski, W. Kozanecki, A. S. Kozhin, V. A. Kramarenko, G. Kramberger, D. Krasnopevtsev, M. W. Krasny, A. Krasznahorkay, J. K. Kraus, A. Kravchenko, M. Kretz, J. Kretzschmar, K. Kreutzfeldt, P. Krieger, K. Krizka, K. Kroeninger, H. Kroha, J. Kroll, J. Kroseberg, J. Krstic, U. Kruchonak, H. Krüger, N. Krumnack, A. Kruse, M. C. Kruse, M. Kruskal, T. Kubota, H. Kucuk, S. Kuday, J. T. Kuechler, S. Kuehn, A. Kugel, F. Kuger, A. Kuhl, T. Kuhl, V. Kukhtin, R. Kukla, Y. Kulchitsky, S. Kuleshov, M. Kuna, T. Kunigo, A. Kupco, H. Kurashige, Y. A. Kurochkin, V. Kus, E. S. Kuwertz, M. Kuze, J. Kvita, T. Kwan, D. Kyriazopoulos, A. La Rosa, J. L. La Rosa Navarro, L. La Rotonda, C. Lacasta, F. Lacava, J. Lacey, H. Lacker, D. Lacour, V. R. Lacuesta, E. Ladygin, R. Lafaye, B. Laforge, T. Lagouri, S. Lai, L. Lambourne, S. Lammers, C. L. Lampen, W. Lampl, E. Lançon, U. Landgraf, M. P. J. Landon, V. S. Lang, J. C. Lange, A. J. Lankford, F. Lanni, K. Lantzsch, A. Lanza, S. Laplace, C. Lapoire, J. F. Laporte, T. Lari, F. Lasagni Manghi, M. Lassnig, P. Laurelli, W. Lavrijsen, A. T. Law, P. Laycock, T. Lazovich, O. Le Dortz, E. Le Guirriec, E. Le Menedeu, M. LeBlanc, T. LeCompte, F. Ledroit-Guillon, C. A. Lee, S. C. Lee, L. Lee, G. Lefebvre, M. Lefebvre, F. Legger, C. Leggett, A. Lehan, G. Lehmann Miotto, X. Lei, W. A. Leight, A. Leisos, A. G. Leister, M. A. L. Leite, R. Leitner, D. Lellouch, B. Lemmer, K. J. C. Leney, T. Lenz, B. Lenzi, R. Leone, S. Leone, C. Leonidopoulos, S. Leontsinis, C. Leroy, C. G. Lester, M. Levchenko, J. Levêque, D. Levin, L. J. Levinson, M. Levy, A. Lewis, A. M. Leyko, M. Leyton, B. Li, H. Li, H. L. Li, L. Li, L. Li, S. Li, X. Li, Y. Li, Z. Liang, H. Liao, B. Liberti, A. Liblong, P. Lichard, K. Lie, J. Liebal, W. Liebig, C. Limbach, A. Limosani, S. C. Lin, T. H. Lin, B. E. Lindquist, E. Lipeles, A. Lipniacka, M. Lisovyi, T. M. Liss, D. Lissauer, A. Lister, A. M. Litke, B. Liu, D. Liu, H. Liu, H. Liu, J. Liu, J. B. Liu, K. Liu, L. Liu, M. Liu, M. Liu, Y. L. Liu, Y. Liu, M. Livan, A. Lleres, J. Llorente Merino, S. L. Lloyd, F. Lo Sterzo, E. Lobodzinska, P. Loch, W. S. Lockman, F. K. Loebinger, A. E. Loevschall-Jensen, K. M. Loew, A. Loginov, T. Lohse, K. Lohwasser, M. Lokajicek, B. A. Long, J. D. Long, R. E. Long, K. A. Looper, L. Lopes, D. Lopez Mateos, B. Lopez Paredes, I. Lopez Paz, A. Lopez Solis, J. Lorenz, N. Lorenzo Martinez, M. Losada, P. J. Lösel, X. Lou, A. Lounis, J. Love, P. A. Love, H. Lu, N. Lu, H. J. Lubatti, C. Luci, A. Lucotte, C. Luedtke, F. Luehring, W. Lukas, L. Luminari, O. Lundberg, B. Lund-Jensen, D. Lynn, R. Lysak, E. Lytken, H. Ma, L. L. Ma, G. Maccarrone, A. Macchiolo, C. M. Macdonald, B. Maček, J. Machado Miguens, D. Madaffari, R. Madar, H. J. Maddocks, W. F. Mader, A. Madsen, J. Maeda, S. Maeland, T. Maeno, A. Maevskiy, E. Magradze, J. Mahlstedt, C. Maiani, C. Maidantchik, A. A. Maier, T. Maier, A. Maio, S. Majewski, Y. Makida, N. Makovec, B. Malaescu, Pa. Malecki, V. P. Maleev, F. Malek, U. Mallik, D. Malon, C. Malone, S. Maltezos, S. Malyukov, J. Mamuzic, G. Mancini, B. Mandelli, L. Mandelli, I. Mandić, J. Maneira, L. Manhaes de Andrade Filho, J. Manjarres Ramos, A. Mann, B. Mansoulie, R. Mantifel, M. Mantoani, S. Manzoni, L. Mapelli, L. March, G. Marchiori, M. Marcisovsky, M. Marjanovic, D. E. Marley, F. Marroquim, S. P. Marsden, Z. Marshall, L. F. Marti, S. Marti-Garcia, B. Martin, T. A. Martin, V. J. Martin, B. Martin dit Latour, M. Martinez, S. Martin-Haugh, V. S. Martoiu, A. C. Martyniuk, M. Marx, F. Marzano, A. Marzin, L. Masetti, T. Mashimo, R. Mashinistov, J. Masik, A. L. Maslennikov, I. Massa, L. Massa, P. Mastrandrea, A. Mastroberardino, T. Masubuchi, P. Mättig, J. Mattmann, J. Maurer, S. J. Maxfield, D. A. Maximov, R. Mazini, S. M. Mazza, N. C. Mc Fadden, G. Mc Goldrick, S. P. Mc Kee, A. McCarn, R. L. McCarthy, T. G. McCarthy, K. W. McFarlane, J. A. Mcfayden, G. Mchedlidze, S. J. McMahon, R. A. McPherson, M. Medinnis, S. Meehan, S. Mehlhase, A. Mehta, K. Meier, C. Meineck, B. Meirose, B. R. Mellado Garcia, F. Meloni, A. Mengarelli, S. Menke, E. Meoni, K. M. Mercurio, S. Mergelmeyer, P. Mermod, L. Merola, C. Meroni, F. S. Merritt, A. Messina, J. Metcalfe, A. S. Mete, C. Meyer, C. Meyer, J-P. Meyer, J. Meyer, H. Meyer Zu Theenhausen, R. P. Middleton, S. Miglioranzi, L. Mijović, G. Mikenberg, M. Mikestikova, M. Mikuž, M. Milesi, A. Milic, D. W. Miller, C. Mills, A. Milov, D. A. Milstead, A. A. Minaenko, Y. Minami, I. A. Minashvili, A. I. Mincer, B. Mindur, M. Mineev, Y. Ming, L. M. Mir, K. P. Mistry, T. Mitani, J. Mitrevski, V. A. Mitsou, A. Miucci, P. S. Miyagawa, J. U. Mjörnmark, T. Moa, K. Mochizuki, S. Mohapatra, W. Mohr, S. Molander, R. Moles-Valls, R. Monden, M. C. Mondragon, K. Mönig, J. Monk, E. Monnier, A. Montalbano, J. Montejo Berlingen, F. Monticelli, S. Monzani, R. W. Moore, N. Morange, D. Moreno, M. Moreno Llácer, P. Morettini, D. Mori, T. Mori, M. Morii, M. Morinaga, V. Morisbak, S. Moritz, A. K. Morley, G. Mornacchi, J. D. Morris, S. S. Mortensen, L. Morvaj, M. Mosidze, J. Moss, K. Motohashi, R. Mount, E. Mountricha, S. V. Mouraviev, E. J. W. Moyse, S. Muanza, R. D. Mudd, F. Mueller, J. Mueller, R. S. P. Mueller, T. Mueller, D. Muenstermann, P. Mullen, G. A. Mullier, F. J. Munoz Sanchez, J. A. Murillo Quijada, W. J. Murray, H. Musheghyan, A. G. Myagkov, M. Myska, B. P. Nachman, O. Nackenhorst, J. Nadal, K. Nagai, R. Nagai, Y. Nagai, K. Nagano, Y. Nagasaka, K. Nagata, M. Nagel, E. Nagy, A. M. Nairz, Y. Nakahama, K. Nakamura, T. Nakamura, I. Nakano, H. Namasivayam, R. F. Naranjo Garcia, R. Narayan, D. I. Narrias Villar, I. Naryshkin, T. Naumann, G. Navarro, R. Nayyar, H. A. Neal, P. Yu. Nechaeva, T. J. Neep, P. D. Nef, A. Negri, M. Negrini, S. Nektarijevic, C. Nellist, A. Nelson, S. Nemecek, P. Nemethy, A. A. Nepomuceno, M. Nessi, M. S. Neubauer, M. Neumann, R. M. Neves, P. Nevski, P. R. Newman, D. H. Nguyen, R. B. Nickerson, R. Nicolaidou, B. Nicquevert, J. Nielsen, A. Nikiforov, V. Nikolaenko, I. Nikolic-Audit, K. Nikolopoulos, J. K. Nilsen, P. Nilsson, Y. Ninomiya, A. Nisati, R. Nisius, T. Nobe, L. Nodulman, M. Nomachi, I. Nomidis, T. Nooney, S. Norberg, M. Nordberg, O. Novgorodova, S. Nowak, M. Nozaki, L. Nozka, K. Ntekas, E. Nurse, F. Nuti, F. O’grady, D. C. O’Neil, V. O’Shea, F. G. Oakham, H. Oberlack, T. Obermann, J. Ocariz, A. Ochi, I. Ochoa, J. P. Ochoa-Ricoux, S. Oda, S. Odaka, H. Ogren, A. Oh, S. H. Oh, C. C. Ohm, H. Ohman, H. Oide, H. Okawa, Y. Okumura, T. Okuyama, A. Olariu, L. F. Oleiro Seabra, S. A. Olivares Pino, D. Oliveira Damazio, A. Olszewski, J. Olszowska, A. Onofre, K. Onogi, P. U. E. Onyisi, C. J. Oram, M. J. Oreglia, Y. Oren, D. Orestano, N. Orlando, R. S. Orr, B. Osculati, R. Ospanov, G. Otero y Garzon, H. Otono, M. Ouchrif, F. Ould-Saada, A. Ouraou, K. P. Oussoren, Q. Ouyang, A. Ovcharova, M. Owen, R. E. Owen, V. E. Ozcan, N. Ozturk, K. Pachal, A. Pacheco Pages, C. Padilla Aranda, M. Pagáčová, S. Pagan Griso, F. Paige, P. Pais, K. Pajchel, G. Palacino, S. Palestini, M. Palka, D. Pallin, A. Palma, E. St. Panagiotopoulou, C. E. Pandini, J. G. Panduro Vazquez, P. Pani, S. Panitkin, D. Pantea, L. Paolozzi, Th. D. Papadopoulou, K. Papageorgiou, A. Paramonov, D. Paredes Hernandez, M. A. Parker, K. A. Parker, F. Parodi, J. A. Parsons, U. Parzefall, V. R. Pascuzzi, E. Pasqualucci, S. Passaggio, F. Pastore, Fr. Pastore, G. Pásztor, S. Pataraia, N. D. Patel, J. R. Pater, T. Pauly, J. Pearce, B. Pearson, L. E. Pedersen, M. Pedersen, S. Pedraza Lopez, R. Pedro, S. V. Peleganchuk, D. Pelikan, O. Penc, C. Peng, H. Peng, B. Penning, J. Penwell, D. V. Perepelitsa, E. Perez Codina, L. Perini, H. Pernegger, S. Perrella, R. Peschke, V. D. Peshekhonov, K. Peters, R. F. Y. Peters, B. A. Petersen, T. C. Petersen, E. Petit, A. Petridis, C. Petridou, P. Petroff, E. Petrolo, F. Petrucci, N. E. Pettersson, A. Peyaud, R. Pezoa, P. W. Phillips, G. Piacquadio, E. Pianori, A. Picazio, E. Piccaro, M. Piccinini, M. A. Pickering, R. Piegaia, J. E. Pilcher, A. D. Pilkington, A. W. J. Pin, J. Pina, M. Pinamonti, J. L. Pinfold, A. Pingel, S. Pires, H. Pirumov, M. Pitt, C. Pizio, L. Plazak, M. -A. Pleier, V. Pleskot, E. Plotnikova, P. Plucinski, D. Pluth, R. Poettgen, L. Poggioli, D. Pohl, G. Polesello, A. Poley, A. Policicchio, R. Polifka, A. Polini, C. S. Pollard, V. Polychronakos, K. Pommès, L. Pontecorvo, B. G. Pope, G. A. Popeneciu, D. S. Popovic, A. Poppleton, S. Pospisil, K. Potamianos, I. N. Potrap, C. J. Potter, C. T. Potter, G. Poulard, J. Poveda, V. Pozdnyakov, M. E. Pozo Astigarraga, P. Pralavorio, A. Pranko, S. Prell, D. Price, L. E. Price, M. Primavera, S. Prince, M. Proissl, K. Prokofiev, F. Prokoshin, E. Protopapadaki, S. Protopopescu, J. Proudfoot, M. Przybycien, D. Puddu, D. Puldon, M. Purohit, P. Puzo, J. Qian, G. Qin, Y. Qin, A. Quadt, D. R. Quarrie, W. B. Quayle, M. Queitsch-Maitland, D. Quilty, S. Raddum, V. Radeka, V. Radescu, S. K. Radhakrishnan, P. Radloff, P. Rados, F. Ragusa, G. Rahal, S. Rajagopalan, M. Rammensee, C. Rangel-Smith, F. Rauscher, S. Rave, T. Ravenscroft, M. Raymond, A. L. Read, N. P. Readioff, D. M. Rebuzzi, A. Redelbach, G. Redlinger, R. Reece, K. Reeves, L. Rehnisch, J. Reichert, H. Reisin, C. Rembser, H. Ren, M. Rescigno, S. Resconi, O. L. Rezanova, P. Reznicek, R. Rezvani, R. Richter, S. Richter, E. Richter-Was, O. Ricken, M. Ridel, P. Rieck, C. J. Riegel, J. Rieger, O. Rifki, M. Rijssenbeek, A. Rimoldi, L. Rinaldi, B. Ristić, E. Ritsch, I. Riu, F. Rizatdinova, E. Rizvi, S. H. Robertson, A. Robichaud-Veronneau, D. Robinson, J. E. M. Robinson, A. Robson, C. Roda, Y. Rodina, A. Rodriguez Perez, S. Roe, C. S. Rogan, O. Røhne, A. Romaniouk, M. Romano, S. M. Romano Saez, E. Romero Adam, N. Rompotis, M. Ronzani, L. Roos, E. Ros, S. Rosati, K. Rosbach, P. Rose, O. Rosenthal, V. Rossetti, E. Rossi, L. P. Rossi, J. H. N. Rosten, R. Rosten, M. Rotaru, I. Roth, J. Rothberg, D. Rousseau, C. R. Royon, A. Rozanov, Y. Rozen, X. Ruan, F. Rubbo, I. Rubinskiy, V. I. Rud, M. S. Rudolph, F. Rühr, A. Ruiz-Martinez, Z. Rurikova, N. A. Rusakovich, A. Ruschke, H. L. Russell, J. P. Rutherfoord, N. Ruthmann, Y. F. Ryabov, M. Rybar, G. Rybkin, N. C. Ryder, A. Ryzhov, A. F. Saavedra, G. Sabato, S. Sacerdoti, H. F-W. Sadrozinski, R. Sadykov, F. Safai Tehrani, P. Saha, M. Sahinsoy, M. Saimpert, T. Saito, H. Sakamoto, Y. Sakurai, G. Salamanna, A. Salamon, J. E. Salazar Loyola, D. Salek, P. H. Sales De Bruin, D. Salihagic, A. Salnikov, J. Salt, D. Salvatore, F. Salvatore, A. Salvucci, A. Salzburger, D. Sammel, D. Sampsonidis, A. Sanchez, J. Sánchez, V. Sanchez Martinez, H. Sandaker, R. L. Sandbach, H. G. Sander, M. P. Sanders, M. Sandhoff, C. Sandoval, R. Sandstroem, D. P. C. Sankey, M. Sannino, A. Sansoni, C. Santoni, R. Santonico, H. Santos, I. Santoyo Castillo, K. Sapp, A. Sapronov, J. G. Saraiva, B. Sarrazin, O. Sasaki, Y. Sasaki, K. Sato, G. Sauvage, E. Sauvan, G. Savage, P. Savard, C. Sawyer, L. Sawyer, J. Saxon, C. Sbarra, A. Sbrizzi, T. Scanlon, D. A. Scannicchio, M. Scarcella, V. Scarfone, J. Schaarschmidt, P. Schacht, D. Schaefer, R. Schaefer, J. Schaeffer, S. Schaepe, S. Schaetzel, U. Schäfer, A. C. Schaffer, D. Schaile, R. D. Schamberger, V. Scharf, V. A. Schegelsky, D. Scheirich, M. Schernau, C. Schiavi, C. Schillo, M. Schioppa, S. Schlenker, K. Schmieden, C. Schmitt, S. Schmitt, S. Schmitt, S. Schmitz, B. Schneider, Y. J. Schnellbach, U. Schnoor, L. Schoeffel, A. Schoening, B. D. Schoenrock, E. Schopf, A. L. S. Schorlemmer, M. Schott, D. Schouten, J. Schovancova, S. Schramm, M. Schreyer, N. Schuh, M. J. Schultens, H. -C. Schultz-Coulon, H. Schulz, M. Schumacher, B. A. Schumm, Ph. Schune, C. Schwanenberger, A. Schwartzman, T. A. Schwarz, Ph. Schwegler, H. Schweiger, Ph. Schwemling, R. Schwienhorst, J. Schwindling, T. Schwindt, G. Sciolla, F. Scuri, F. Scutti, J. Searcy, P. Seema, S. C. Seidel, A. Seiden, F. Seifert, J. M. Seixas, G. Sekhniaidze, K. Sekhon, S. J. Sekula, D. M. Seliverstov, N. Semprini-Cesari, C. Serfon, L. Serin, L. Serkin, M. Sessa, R. Seuster, H. Severini, T. Sfiligoj, F. Sforza, A. Sfyrla, E. Shabalina, N. W. Shaikh, L. Y. Shan, R. Shang, J. T. Shank, M. Shapiro, P. B. Shatalov, K. Shaw, S. M. Shaw, A. Shcherbakova, C. Y. Shehu, P. Sherwood, L. Shi, S. Shimizu, C. O. Shimmin, M. Shimojima, M. Shiyakova, A. Shmeleva, D. Shoaleh Saadi, M. J. Shochet, S. Shojaii, S. Shrestha, E. Shulga, M. A. Shupe, P. Sicho, P. E. Sidebo, O. Sidiropoulou, D. Sidorov, A. Sidoti, F. Siegert, Dj. Sijacki, J. Silva, S. B. Silverstein, V. Simak, O. Simard, Lj. Simic, S. Simion, E. Simioni, B. Simmons, D. Simon, M. Simon, R. Simoniello, P. Sinervo, N. B. Sinev, M. Sioli, G. Siragusa, S. Yu. Sivoklokov, J. Sjölin, T. B. Sjursen, M. B. Skinner, H. P. Skottowe, P. Skubic, M. Slater, T. Slavicek, M. Slawinska, K. Sliwa, V. Smakhtin, B. H. Smart, L. Smestad, S. Yu. Smirnov, Y. Smirnov, L. N. Smirnova, O. Smirnova, M. N. K. Smith, R. W. Smith, M. Smizanska, K. Smolek, A. A. Snesarev, G. Snidero, S. Snyder, R. Sobie, F. Socher, A. Soffer, D. A. Soh, G. Sokhrannyi, C. A. Solans Sanchez, M. Solar, E. Yu. Soldatov, U. Soldevila, A. A. Solodkov, A. Soloshenko, O. V. Solovyanov, V. Solovyev, P. Sommer, H. Y. Song, N. Soni, A. Sood, A. Sopczak, V. Sopko, V. Sorin, D. Sosa, C. L. Sotiropoulou, R. Soualah, A. M. Soukharev, D. South, B. C. Sowden, S. Spagnolo, M. Spalla, M. Spangenberg, F. Spanò, D. Sperlich, F. Spettel, R. Spighi, G. Spigo, L. A. Spiller, M. Spousta, R. D. St. Denis, A. Stabile, J. Stahlman, R. Stamen, S. Stamm, E. Stanecka, R. W. Stanek, C. Stanescu, M. Stanescu-Bellu, M. M. Stanitzki, S. Stapnes, E. A. Starchenko, G. H. Stark, J. Stark, P. Staroba, P. Starovoitov, S. Stärz, R. Staszewski, P. Steinberg, B. Stelzer, H. J. Stelzer, O. Stelzer-Chilton, H. Stenzel, G. A. Stewart, J. A. Stillings, M. C. Stockton, M. Stoebe, G. Stoicea, P. Stolte, S. Stonjek, A. R. Stradling, A. Straessner, M. E. Stramaglia, J. Strandberg, S. Strandberg, A. Strandlie, M. Strauss, P. Strizenec, R. Ströhmer, D. M. Strom, R. Stroynowski, A. Strubig, S. A. Stucci, B. Stugu, N. A. Styles, D. Su, J. Su, R. Subramaniam, S. Suchek, Y. Sugaya, M. Suk, V. V. Sulin, S. Sultansoy, T. Sumida, S. Sun, X. Sun, J. E. Sundermann, K. Suruliz, G. Susinno, M. R. Sutton, S. Suzuki, M. Svatos, M. Swiatlowski, I. Sykora, T. Sykora, D. Ta, C. Taccini, K. Tackmann, J. Taenzer, A. Taffard, R. Tafirout, N. Taiblum, H. Takai, R. Takashima, H. Takeda, T. Takeshita, Y. Takubo, M. Talby, A. A. Talyshev, J. Y. C. Tam, K. G. Tan, J. Tanaka, R. Tanaka, S. Tanaka, B. B. Tannenwald, S. Tapia Araya, S. Tapprogge, S. Tarem, G. F. Tartarelli, P. Tas, M. Tasevsky, T. Tashiro, E. Tassi, A. Tavares Delgado, Y. Tayalati, A. C. Taylor, G. N. Taylor, P. T. E. Taylor, W. Taylor, F. A. Teischinger, P. Teixeira-Dias, K. K. Temming, D. Temple, H. Ten Kate, P. K. Teng, J. J. Teoh, F. Tepel, S. Terada, K. Terashi, J. Terron, S. Terzo, M. Testa, R. J. Teuscher, T. Theveneaux-Pelzer, J. P. Thomas, J. Thomas-Wilsker, E. N. Thompson, P. D. Thompson, R. J. Thompson, A. S. Thompson, L. A. Thomsen, E. Thomson, M. Thomson, M. J. Tibbetts, R. E. Ticse Torres, V. O. Tikhomirov, Yu. A. Tikhonov, S. Timoshenko, E. Tiouchichine, P. Tipton, S. Tisserant, K. Todome, T. Todorov, S. Todorova-Nova, J. Tojo, S. Tokár, K. Tokushuku, E. Tolley, L. Tomlinson, M. Tomoto, L. Tompkins, K. Toms, B. Tong, E. Torrence, H. Torres, E. Torró Pastor, J. Toth, F. Touchard, D. R. Tovey, T. Trefzger, A. Tricoli, I. M. Trigger, S. Trincaz-Duvoid, M. F. Tripiana, W. Trischuk, B. Trocmé, A. Trofymov, C. Troncon, M. Trottier-McDonald, M. Trovatelli, L. Truong, M. Trzebinski, A. Trzupek, J. C-L. Tseng, P. V. Tsiareshka, G. Tsipolitis, N. Tsirintanis, S. Tsiskaridze, V. Tsiskaridze, E. G. Tskhadadze, K. M. Tsui, I. I. Tsukerman, V. Tsulaia, S. Tsuno, D. Tsybychev, A. Tudorache, V. Tudorache, A. N. Tuna, S. A. Tupputi, S. Turchikhin, D. Turecek, D. Turgeman, R. Turra, A. J. Turvey, P. M. Tuts, M. Tylmad, M. Tyndel, I. Ueda, R. Ueno, M. Ughetto, F. Ukegawa, G. Unal, A. Undrus, G. Unel, F. C. Ungaro, Y. Unno, C. Unverdorben, J. Urban, P. Urquijo, P. Urrejola, G. Usai, A. Usanova, L. Vacavant, V. Vacek, B. Vachon, C. Valderanis, N. Valencic, S. Valentinetti, A. Valero, L. Valery, S. Valkar, S. Vallecorsa, J. A. Valls Ferrer, W. Van Den Wollenberg, P. C. Van Der Deijl, R. van der Geer, H. van der Graaf, N. van Eldik, P. van Gemmeren, J. Van Nieuwkoop, I. van Vulpen, M. C. van Woerden, M. Vanadia, W. Vandelli, R. Vanguri, A. Vaniachine, G. Vardanyan, R. Vari, E. W. Varnes, T. Varol, D. Varouchas, A. Vartapetian, K. E. Varvell, F. Vazeille, T. Vazquez Schroeder, J. Veatch, L. M. Veloce, F. Veloso, S. Veneziano, A. Ventura, M. Venturi, N. Venturi, A. Venturini, V. Vercesi, M. Verducci, W. Verkerke, J. C. Vermeulen, A. Vest, M. C. Vetterli, O. Viazlo, I. Vichou, T. Vickey, O. E. Vickey Boeriu, G. H. A. Viehhauser, S. Viel, R. Vigne, M. Villa, M. Villaplana Perez, E. Vilucchi, M. G. Vincter, V. B. Vinogradov, I. Vivarelli, S. Vlachos, D. Vladoiu, M. Vlasak, M. Vogel, P. Vokac, G. Volpi, M. Volpi, H. von der Schmitt, E. von Toerne, V. Vorobel, K. Vorobev, M. Vos, R. Voss, J. H. Vossebeld, N. Vranjes, M. Vranjes Milosavljevic, V. Vrba, M. Vreeswijk, R. Vuillermet, I. Vukotic, Z. Vykydal, P. Wagner, W. Wagner, H. Wahlberg, S. Wahrmund, J. Wakabayashi, J. Walder, R. Walker, W. Walkowiak, V. Wallangen, C. Wang, C. Wang, F. Wang, H. Wang, H. Wang, J. Wang, J. Wang, K. Wang, R. Wang, S. M. Wang, T. Wang, T. Wang, X. Wang, C. Wanotayaroj, A. Warburton, C. P. Ward, D. R. Wardrope, A. Washbrook, P. M. Watkins, A. T. Watson, I. J. Watson, M. F. Watson, G. Watts, S. Watts, B. M. Waugh, S. Webb, M. S. Weber, S. W. Weber, J. S. Webster, A. R. Weidberg, B. Weinert, J. Weingarten, C. Weiser, H. Weits, P. S. Wells, T. Wenaus, T. Wengler, S. Wenig, N. Wermes, M. Werner, P. Werner, M. Wessels, J. Wetter, K. Whalen, A. M. Wharton, A. White, M. J. White, R. White, S. White, D. Whiteson, F. J. Wickens, W. Wiedenmann, M. Wielers, P. Wienemann, C. Wiglesworth, L. A. M. Wiik-Fuchs, A. Wildauer, H. G. Wilkens, H. H. Williams, S. Williams, C. Willis, S. Willocq, J. A. Wilson, I. Wingerter-Seez, F. Winklmeier, B. T. Winter, M. Wittgen, J. Wittkowski, S. J. Wollstadt, M. W. Wolter, H. Wolters, B. K. Wosiek, J. Wotschack, M. J. Woudstra, K. W. Wozniak, M. Wu, M. Wu, S. L. Wu, X. Wu, Y. Wu, T. R. Wyatt, B. M. Wynne, S. Xella, D. Xu, L. Xu, B. Yabsley, S. Yacoob, R. Yakabe, D. Yamaguchi, Y. Yamaguchi, A. Yamamoto, S. Yamamoto, T. Yamanaka, K. Yamauchi, Y. Yamazaki, Z. Yan, H. Yang, H. Yang, Y. Yang, Z. Yang, W-M. Yao, Y. C. Yap, Y. Yasu, E. Yatsenko, K. H. Yau Wong, J. Ye, S. Ye, I. Yeletskikh, A. L. Yen, E. Yildirim, K. Yorita, R. Yoshida, K. Yoshihara, C. Young, C. J. S. Young, S. Youssef, D. R. Yu, J. Yu, J. M. Yu, J. Yu, L. Yuan, S. P. Y. Yuen, I. Yusuff, B. Zabinski, R. Zaidan, A. M. Zaitsev, N. Zakharchuk, J. Zalieckas, A. Zaman, S. Zambito, L. Zanello, D. Zanzi, C. Zeitnitz, M. Zeman, A. Zemla, J. C. Zeng, Q. Zeng, K. Zengel, O. Zenin, T. Ženiš, D. Zerwas, D. Zhang, F. Zhang, G. Zhang, H. Zhang, J. Zhang, L. Zhang, R. Zhang, R. Zhang, X. Zhang, Z. Zhang, X. Zhao, Y. Zhao, Z. Zhao, A. Zhemchugov, J. Zhong, B. Zhou, C. Zhou, L. Zhou, L. Zhou, M. Zhou, N. Zhou, C. G. Zhu, H. Zhu, J. Zhu, Y. Zhu, X. Zhuang, K. Zhukov, A. Zibell, D. Zieminska, N. I. Zimine, C. Zimmermann, S. Zimmermann, Z. Zinonos, M. Zinser, M. Ziolkowski, L. Živković, G. Zobernig, A. Zoccoli, M. zur Nedden, G. Zurzolo, L. Zwalinski, ATLAS Collaboration

**Affiliations:** 1 Department of Physics, University of Adelaide, Adelaide, Australia; 2 Physics Department, SUNY Albany, Albany, NY USA; 3 Department of Physics, University of Alberta, Edmonton, AB Canada; 4 Department of Physics, Ankara University, Ankara, Turkey; 5 Istanbul Aydin University, Istanbul, Turkey; 6 Division of Physics, TOBB University of Economics and Technology, Ankara, Turkey; 7 LAPP, CNRS/IN2P3 and Université Savoie Mont Blanc, Annecy-le-Vieux, France; 8 High Energy Physics Division, Argonne National Laboratory, Argonne, IL USA; 9 Department of Physics, University of Arizona, Tucson, AZ USA; 10 Department of Physics, The University of Texas at Arlington, Arlington, TX USA; 11 Physics Department, University of Athens, Athens, Greece; 12 Physics Department, National Technical University of Athens, Zografou, Greece; 13 Department of Physics, The University of Texas at Austin, Austin, TX USA; 14 Institute of Physics, Azerbaijan Academy of Sciences, Baku, Azerbaijan; 15 Institut de Física d’Altes Energies (IFAE), The Barcelona Institute of Science and Technology, Barcelona, Spain; 16 Institute of Physics, University of Belgrade, Belgrade, Serbia; 17 Department for Physics and Technology, University of Bergen, Bergen, Norway; 18 Physics Division, Lawrence Berkeley National Laboratory and University of California, Berkeley, CA USA; 19 Department of Physics, Humboldt University, Berlin, Germany; 20 Albert Einstein Center for Fundamental Physics and Laboratory for High Energy Physics, University of Bern, Bern, Switzerland; 21 School of Physics and Astronomy, University of Birmingham, Birmingham, UK; 22 Department of Physics, Bogazici University, Istanbul, Turkey; 23 Department of Physics Engineering, Gaziantep University, Gaziantep, Turkey; 24 Department of Physics, Dogus University, Istanbul, Turkey; 25 Centro de Investigaciones, Universidad Antonio Narino, Bogota, Colombia; 26 INFN Sezione di Bologna, Bologna, Italy; 27 Dipartimento di Fisica e Astronomia, Università di Bologna, Bologna, Italy; 28 Physikalisches Institut, University of Bonn, Bonn, Germany; 29 Department of Physics, Boston University, Boston, MA USA; 30 Department of Physics, Brandeis University, Waltham, MA USA; 31 Universidade Federal do Rio De Janeiro COPPE/EE/IF, Rio de Janeiro, Brazil; 32 Electrical Circuits Department, Federal University of Juiz de Fora (UFJF), Juiz de Fora, Brazil; 33 Federal University of Sao Joao del Rei (UFSJ), Sao Joao del Rei, Brazil; 34 Instituto de Fisica, Universidade de Sao Paulo, Sao Paulo, Brazil; 35 Physics Department, Brookhaven National Laboratory, Upton, NY USA; 36 Transilvania University of Brasov, Brasov, Romania; 37 National Institute of Physics and Nuclear Engineering, Bucharest, Romania; 38 Physics Department, National Institute for Research and Development of Isotopic and Molecular Technologies, Cluj Napoca, Romania; 39 University Politehnica Bucharest, Bucharest, Romania; 40 West University in Timisoara, Timisoara, Romania; 41 Departamento de Física, Universidad de Buenos Aires, Buenos Aires, Argentina; 42 Cavendish Laboratory, University of Cambridge, Cambridge, UK; 43 Department of Physics, Carleton University, Ottawa, ON Canada; 44 CERN, Geneva, Switzerland; 45 Enrico Fermi Institute, University of Chicago, Chicago, IL USA; 46 Departamento de Física, Pontificia Universidad Católica de Chile, Santiago, Chile; 47 Departamento de Física, Universidad Técnica Federico Santa María, Valparaiso, Chile; 48 Institute of High Energy Physics, Chinese Academy of Sciences, Beijing, China; 49 Department of Modern Physics, University of Science and Technology of China, Anhui, China; 50 Department of Physics, Nanjing University, Jiangsu, China; 51 School of Physics, Shandong University, Shandong, China; 52 Department of Physics and Astronomy, Shanghai Key Laboratory for Particle Physics and Cosmology, Shanghai Jiao Tong University, Shanghai (also affiliated with PKU-CHEP), China; 53 Physics Department, Tsinghua University, Beijing, 100084 China; 54 Laboratoire de Physique Corpusculaire, Clermont Université and Université Blaise Pascal and CNRS/IN2P3, Clermont-Ferrand, France; 55 Nevis Laboratory, Columbia University, Irvington, NY USA; 56 Niels Bohr Institute, University of Copenhagen, Kobenhavn, Denmark; 57 INFN Gruppo Collegato di Cosenza, Laboratori Nazionali di Frascati, Frascati, Italy; 58 Dipartimento di Fisica, Università della Calabria, Rende, Italy; 59 Faculty of Physics and Applied Computer Science, AGH University of Science and Technology, Kraków, Poland; 60 Marian Smoluchowski Institute of Physics, Jagiellonian University, Kraków, Poland; 61 Institute of Nuclear Physics, Polish Academy of Sciences, Kraków, Poland; 62 Physics Department, Southern Methodist University, Dallas, TX USA; 63 Physics Department, University of Texas at Dallas, Richardson, TX USA; 64 DESY, Hamburg and Zeuthen, Germany; 65 Institut für Experimentelle Physik IV, Technische Universität Dortmund, Dortmund, Germany; 66 Institut für Kern-und Teilchenphysik, Technische Universität Dresden, Dresden, Germany; 67 Department of Physics, Duke University, Durham, NC USA; 68 SUPA-School of Physics and Astronomy, University of Edinburgh, Edinburgh, UK; 69 INFN Laboratori Nazionali di Frascati, Frascati, Italy; 70 Fakultät für Mathematik und Physik, Albert-Ludwigs-Universität, Freiburg, Germany; 71 Section de Physique, Université de Genève, Geneva, Switzerland; 72 INFN Sezione di Genova, Genoa, Italy; 73 Dipartimento di Fisica, Università di Genova, Genoa, Italy; 74 E. Andronikashvili Institute of Physics, Iv. Javakhishvili Tbilisi State University, Tbilisi, Georgia; 75 High Energy Physics Institute, Tbilisi State University, Tbilisi, Georgia; 76 II Physikalisches Institut, Justus-Liebig-Universität Giessen, Giessen, Germany; 77 SUPA-School of Physics and Astronomy, University of Glasgow, Glasgow, UK; 78 II Physikalisches Institut, Georg-August-Universität, Göttingen, Germany; 79 Laboratoire de Physique Subatomique et de Cosmologie, Université Grenoble-Alpes, CNRS/IN2P3, Grenoble, France; 80 Department of Physics, Hampton University, Hampton, VA USA; 81 Laboratory for Particle Physics and Cosmology, Harvard University, Cambridge, MA USA; 82 Kirchhoff-Institut für Physik, Ruprecht-Karls-Universität Heidelberg, Heidelberg, Germany; 83 Physikalisches Institut, Ruprecht-Karls-Universität Heidelberg, Heidelberg, Germany; 84 ZITI Institut für technische Informatik, Ruprecht-Karls-Universität Heidelberg, Mannheim, Germany; 85 Faculty of Applied Information Science, Hiroshima Institute of Technology, Hiroshima, Japan; 86 Department of Physics, The Chinese University of Hong Kong, Shatin, N.T. Hong Kong; 87 Department of Physics, The University of Hong Kong, Hong Kong, China; 88 Department of Physics, The Hong Kong University of Science and Technology, Clear Water Bay, Kowloon, Hong Kong, China; 89 Department of Physics, Indiana University, Bloomington, IN USA; 90 Institut für Astro- und Teilchenphysik, Leopold-Franzens-Universität, Innsbruck, Austria; 91 University of Iowa, Iowa City, IA USA; 92 Department of Physics and Astronomy, Iowa State University, Ames, IA USA; 93 Joint Institute for Nuclear Research, JINR Dubna, Dubna, Russia; 94 KEK, High Energy Accelerator Research Organization, Tsukuba, Japan; 95 Graduate School of Science, Kobe University, Kobe, Japan; 96 Faculty of Science, Kyoto University, Kyoto, Japan; 97 Kyoto University of Education, Kyoto, Japan; 98 Department of Physics, Kyushu University, Fukuoka, Japan; 99 Instituto de Física La Plata, Universidad Nacional de La Plata and CONICET, La Plata, Argentina; 100 Physics Department, Lancaster University, Lancaster, UK; 101 INFN Sezione di Lecce, Lecce, Italy; 102 Dipartimento di Matematica e Fisica, Università del Salento, Lecce, Italy; 103 Oliver Lodge Laboratory, University of Liverpool, Liverpool, UK; 104 Department of Physics, Jožef Stefan Institute, University of Ljubljana, Ljubljana, Slovenia; 105 School of Physics and Astronomy, Queen Mary University of London, London, UK; 106 Department of Physics, Royal Holloway University of London, Surrey, UK; 107 Department of Physics and Astronomy, University College London, London, UK; 108 Louisiana Tech University, Ruston, LA USA; 109 Laboratoire de Physique Nucléaire et de Hautes Energies, UPMC and Université Paris-Diderot and CNRS/IN2P3, Paris, France; 110 Fysiska institutionen, Lunds universitet, Lund, Sweden; 111 Departamento de Fisica Teorica C-15, Universidad Autonoma de Madrid, Madrid, Spain; 112 Institut für Physik, Universität Mainz, Mainz, Germany; 113 School of Physics and Astronomy, University of Manchester, Manchester, UK; 114 CPPM, Aix-Marseille Université and CNRS/IN2P3, Marseille, France; 115 Department of Physics, University of Massachusetts, Amherst, MA USA; 116 Department of Physics, McGill University, Montreal, QC Canada; 117 School of Physics, University of Melbourne, Victoria, Australia; 118 Department of Physics, The University of Michigan, Ann Arbor, MI USA; 119 Department of Physics and Astronomy, Michigan State University, East Lansing, MI USA; 120 INFN Sezione di Milano, Milan, Italy; 121 Dipartimento di Fisica, Università di Milano, Milan, Italy; 122 B.I. Stepanov Institute of Physics, National Academy of Sciences of Belarus, Minsk, Republic of Belarus; 123 National Scientific and Educational Centre for Particle and High Energy Physics, Minsk, Republic of Belarus; 124 Group of Particle Physics, University of Montreal, Montreal, QC Canada; 125 P.N. Lebedev Physical Institute of the Russian Academy of Sciences, Moscow, Russia; 126 Institute for Theoretical and Experimental Physics (ITEP), Moscow, Russia; 127 National Research Nuclear University MEPhI, Moscow, Russia; 128 D.V. Skobeltsyn Institute of Nuclear Physics, M.V. Lomonosov Moscow State University, Moscow, Russia; 129 Fakultät für Physik, Ludwig-Maximilians-Universität München, Munich, Germany; 130 Max-Planck-Institut für Physik (Werner-Heisenberg-Institut), Munich, Germany; 131 Nagasaki Institute of Applied Science, Nagasaki, Japan; 132 Graduate School of Science and Kobayashi-Maskawa Institute, Nagoya University, Nagoya, Japan; 133 INFN Sezione di Napoli, Naples, Italy; 134 Dipartimento di Fisica, Università di Napoli, Naples, Italy; 135 Department of Physics and Astronomy, University of New Mexico, Albuquerque, NM USA; 136 Institute for Mathematics, Astrophysics and Particle Physics, Radboud University Nijmegen/Nikhef, Nijmegen, The Netherlands; 137 Nikhef National Institute for Subatomic Physics and University of Amsterdam, Amsterdam, The Netherlands; 138 Department of Physics, Northern Illinois University, DeKalb, IL USA; 139 Budker Institute of Nuclear Physics, SB RAS, Novosibirsk, Russia; 140 Department of Physics, New York University, New York, NY USA; 141 Ohio State University, Columbus, OH USA; 142 Faculty of Science, Okayama University, Okayama, Japan; 143 Homer L. Dodge Department of Physics and Astronomy, University of Oklahoma, Norman, OK USA; 144 Department of Physics, Oklahoma State University, Stillwater, OK USA; 145 Palacký University, RCPTM, Olomouc, Czech Republic; 146 Center for High Energy Physics, University of Oregon, Eugene, OR USA; 147 LAL, Univ. Paris-Sud, CNRS/IN2P3, Université Paris-Saclay, Orsay, France; 148 Graduate School of Science, Osaka University, Osaka, Japan; 149 Department of Physics, University of Oslo, Oslo, Norway; 150 Department of Physics, Oxford University, Oxford, UK; 151 INFN Sezione di Pavia, Pavia, Italy; 152 Dipartimento di Fisica, Università di Pavia, Pavia, Italy; 153 Department of Physics, University of Pennsylvania, Philadelphia, PA USA; 154 National Research Centre “Kurchatov Institute” B.P.Konstantinov Petersburg Nuclear Physics Institute, St. Petersburg, Russia; 155 INFN Sezione di Pisa, Pisa, Italy; 156 Dipartimento di Fisica E. Fermi, Università di Pisa, Pisa, Italy; 157 Department of Physics and Astronomy, University of Pittsburgh, Pittsburgh, PA USA; 158 Laboratório de Instrumentação e Física Experimental de Partículas-LIP, Lisbon, Portugal; 159 Faculdade de Ciências, Universidade de Lisboa, Lisbon, Portugal; 160 Department of Physics, University of Coimbra, Coimbra, Portugal; 161 Centro de Física Nuclear da Universidade de Lisboa, Lisbon, Portugal; 162 Departamento de Fisica, Universidade do Minho, Braga, Portugal; 163 Departamento de Fisica Teorica y del Cosmos and CAFPE, Universidad de Granada, Granada, Spain; 164 Dep Fisica and CEFITEC of Faculdade de Ciencias e Tecnologia, Universidade Nova de Lisboa, Caparica, Portugal; 165 Institute of Physics, Academy of Sciences of the Czech Republic, Praha, Czech Republic; 166 Czech Technical University in Prague, Praha, Czech Republic; 167 Faculty of Mathematics and Physics, Charles University in Prague, Praha, Czech Republic; 168 State Research Center Institute for High Energy Physics (Protvino), NRC KI, Protvino, Russia; 169 Particle Physics Department, Rutherford Appleton Laboratory, Didcot, UK; 170 INFN Sezione di Roma, Rome, Italy; 171 Dipartimento di Fisica, Sapienza Università di Roma, Rome, Italy; 172 INFN Sezione di Roma Tor Vergata, Rome, Italy; 173 Dipartimento di Fisica, Università di Roma Tor Vergata, Rome, Italy; 174 INFN Sezione di Roma Tre, Rome, Italy; 175 Dipartimento di Matematica e Fisica, Università Roma Tre, Rome, Italy; 176 Faculté des Sciences Ain Chock, Réseau Universitaire de Physique des Hautes Energies-Université Hassan II, Casablanca, Morocco; 177 Centre National de l’Energie des Sciences Techniques Nucleaires, Rabat, Morocco; 178 Faculté des Sciences Semlalia, Université Cadi Ayyad, LPHEA-Marrakech, Marrakech, Morocco; 179 Faculté des Sciences, Université Mohamed Premier and LPTPM, Oujda, Morocco; 180 Faculté des Sciences, Université Mohammed V, Rabat, Morocco; 181 DSM/IRFU (Institut de Recherches sur les Lois Fondamentales de l’Univers), CEA Saclay (Commissariat à l’Energie Atomique et aux Energies Alternatives), Gif-sur-Yvette, France; 182 Santa Cruz Institute for Particle Physics, University of California Santa Cruz, Santa Cruz, CA USA; 183 Department of Physics, University of Washington, Seattle, WA USA; 184 Department of Physics and Astronomy, University of Sheffield, Sheffield, UK; 185 Department of Physics, Shinshu University, Nagano, Japan; 186 Fachbereich Physik, Universität Siegen, Siegen, Germany; 187 Department of Physics, Simon Fraser University, Burnaby, BC Canada; 188 SLAC National Accelerator Laboratory, Stanford, CA USA; 189 Faculty of Mathematics, Physics and Informatics, Comenius University, Bratislava, Slovak Republic; 190 Department of Subnuclear Physics, Institute of Experimental Physics of the Slovak Academy of Sciences, Kosice, Slovak Republic; 191 Department of Physics, University of Cape Town, Cape Town, South Africa; 192 Department of Physics, University of Johannesburg, Johannesburg, South Africa; 193 School of Physics, University of the Witwatersrand, Johannesburg, South Africa; 194 Department of Physics, Stockholm University, Stockholm, Sweden; 195 The Oskar Klein Centre, Stockholm, Sweden; 196 Physics Department, Royal Institute of Technology, Stockholm, Sweden; 197 Departments of Physics and Astronomy and Chemistry, Stony Brook University, Stony Brook, NY USA; 198 Department of Physics and Astronomy, University of Sussex, Brighton, UK; 199 School of Physics, University of Sydney, Sydney, Australia; 200 Institute of Physics, Academia Sinica, Taipei, Taiwan; 201 Department of Physics, Technion: Israel Institute of Technology, Haifa, Israel; 202 Raymond and Beverly Sackler School of Physics and Astronomy, Tel Aviv University, Tel Aviv, Israel; 203 Department of Physics, Aristotle University of Thessaloniki, Thessaloniki, Greece; 204 International Center for Elementary Particle Physics and Department of Physics, The University of Tokyo, Tokyo, Japan; 205 Graduate School of Science and Technology, Tokyo Metropolitan University, Tokyo, Japan; 206 Department of Physics, Tokyo Institute of Technology, Tokyo, Japan; 207 Department of Physics, University of Toronto, Toronto, ON Canada; 208 TRIUMF, Vancouver, BC Canada; 209 Department of Physics and Astronomy, York University, Toronto, ON Canada; 210 Faculty of Pure and Applied Sciences, and Center for Integrated Research in Fundamental Science and Engineering, University of Tsukuba, Tsukuba, Japan; 211 Department of Physics and Astronomy, Tufts University, Medford, MA USA; 212 Department of Physics and Astronomy, University of California Irvine, Irvine, CA USA; 213 INFN Gruppo Collegato di Udine, Sezione di Trieste, Udine, Italy; 214 ICTP, Trieste, Italy; 215 Dipartimento di Chimica Fisica e Ambiente, Università di Udine, Udine, Italy; 216 Department of Physics and Astronomy, University of Uppsala, Uppsala, Sweden; 217 Department of Physics, University of Illinois, Urbana, IL USA; 218 Instituto de Fisica Corpuscular (IFIC) and Departamento de Fisica Atomica, Molecular y Nuclear and Departamento de Ingeniería Electrónica and Instituto de Microelectrónica de Barcelona (IMB-CNM), University of Valencia and CSIC, Valencia, Spain; 219 Department of Physics, University of British Columbia, Vancouver, BC Canada; 220 Department of Physics and Astronomy, University of Victoria, Victoria, BC Canada; 221 Department of Physics, University of Warwick, Coventry, UK; 222 Waseda University, Tokyo, Japan; 223 Department of Particle Physics, The Weizmann Institute of Science, Rehovot, Israel; 224 Department of Physics, University of Wisconsin, Madison, WI USA; 225 Fakultät für Physik und Astronomie, Julius-Maximilians-Universität, Würzburg, Germany; 226 Fakultät für Mathematik und Naturwissenschaften, Fachgruppe Physik, Bergische Universität Wuppertal, Wuppertal, Germany; 227 Department of Physics, Yale University, New Haven, CT USA; 228 Yerevan Physics Institute, Yerevan, Armenia; 229 Centre de Calcul de l’Institut National de Physique Nucléaire et de Physique des Particules (IN2P3), Villeurbanne, France; 230 CERN, 1211 Geneva 23, Switzerland

## Abstract

The reconstruction and calibration algorithms used to calculate missing transverse momentum ($E_{\text {T}}^{\text {miss}}$ ) with the ATLAS detector exploit energy deposits in the calorimeter and tracks reconstructed in the inner detector as well as the muon spectrometer. Various strategies are used to suppress effects arising from additional proton–proton interactions, called pileup, concurrent with the hard-scatter processes. Tracking information is used to distinguish contributions from the pileup interactions using their vertex separation along the beam axis. The performance of the $E_{\text {T}}^{\text {miss}}$ reconstruction algorithms, especially with respect to the amount of pileup, is evaluated using data collected in proton–proton collisions at a centre-of-mass energy of 8 $\text {TeV}$ during 2012, and results are shown for a data sample corresponding to an integrated luminosity of $20.3\, \mathrm{fb}^{-1}$. The simulation and modelling of $E_{\text {T}}^{\text {miss}}$  in events containing a *Z* boson decaying to two charged leptons (electrons or muons) or a *W* boson decaying to a charged lepton and a neutrino are compared to data. The acceptance for different event topologies, with and without high transverse momentum neutrinos, is shown for a range of threshold criteria for $E_{\text {T}}^{\text {miss}}$ , and estimates of the systematic uncertainties in the $E_{\text {T}}^{\text {miss}}$  measurements are presented.

## Introduction

The Large Hadron Collider (LHC) provided proton–proton (*pp*) collisions at a centre-of-mass energy of 8 $\text {TeV}$ during 2012. Momentum conservation transverse to the beam axis^1^ implies that the transverse momenta of all particles in the final state should sum to zero. Any imbalance may indicate the presence of undetectable particles such as neutrinos or new, stable particles escaping detection.

The missing transverse momentum ($\vec {E}_{{\mathrm{T}}}^{\mathrm{miss}}$) is reconstructed as the negative vector sum of the transverse momenta ($\vec {p_{\text {T}}}$ ) of all detected particles, and its magnitude is represented by the symbol $E_{\mathrm {T}}^{\mathrm {miss}}$. The measurement of $E_{\text {T}}^{\text {miss}}$  strongly depends on the energy scale and resolution of the reconstructed “physics objects”. The physics objects considered in the $E_{\text {T}}^{\text {miss}}$  calculation are electrons, photons, muons, $\tau$-leptons, and jets. Momentum contributions not attributed to any of the physics objects mentioned above are reconstructed as the $E_{\text {T}}^{\text {miss}}$ “soft term”. Several algorithms for reconstructing the $E_{\text {T}}^{\text {miss}}$  soft term utilizing a combination of calorimeter signals and tracks in the inner detector are considered.

The $E_{\text {T}}^{\text {miss}}$ reconstruction algorithms and calibrations developed by ATLAS for 7 $\text {TeV}$ data from 2010 are summarized in Ref. [1]. The 2011 and 2012 datasets are more affected by contributions from additional *pp* collisions, referred to as “pileup”, concurrent with the hard-scatter process. Various techniques have been developed to suppress such contributions. This paper describes the pileup dependence, calibration, and resolution of the $E_{\text {T}}^{\text {miss}}$ reconstructed with different algorithms and pileup-mitigation techniques.

The performance of $E_{\text {T}}^{\text {miss}}$  reconstruction algorithms, or “$E_{\text {T}}^{\text {miss}}$  performance”, refers to the use of derived quantities like the mean, width, or tail of the $E_{\text {T}}^{\text {miss}}$ distribution to study pileup dependence and calibration. The $E_{\text {T}}^{\text {miss}}$ reconstructed with different algorithms is studied in both data and Monte Carlo (MC) simulation, and the level of agreement between the two is compared using datasets in which events with a leptonically decaying *W* or *Z* boson dominate. The *W* boson sample provides events with intrinsic $E_{\text {T}}^{\text {miss}}$ from non-interacting particles (e.g. neutrinos). Contributions to the $E_{\text {T}}^{\text {miss}}$ due to mismeasurement are referred to as fake $E_{\text {T}}^{\text {miss}}$ . Sources of fake $E_{\text {T}}^{\text {miss}}$ may include ${p}_{\text {T}}$ mismeasurement, miscalibration, and particles going through un-instrumented regions of the detector. In MC simulations, the $E_{\text {T}}^{\text {miss}}$ from each algorithm is compared to the true $E_{\text {T}}^{\text {miss}}$  ($E_{\mathrm {T}}^{\mathrm {miss,True}}$), which is defined as the magnitude of the vector sum of $\vec {p_{\text {T}}}$  of stable^2^ weakly interacting particles from the hard-scatter collision. Then the selection efficiency after a $E_{\text {T}}^{\text {miss}}$-threshold requirement is studied in simulated events with high-${p}_{\text {T}}$  neutrinos (such as top-quark pair production and vector-boson fusion $H \rightarrow \tau \tau$) or possible new weakly interacting particles that escape detection (such as the lightest supersymmetric particles).

This paper is organized as follows. Section 2 gives a brief introduction to the ATLAS detector. Section 3 describes the data and MC simulation used as well as the event selections applied. Section 4 outlines how the $E_{\text {T}}^{\text {miss}}$  is reconstructed and calibrated while Sect. 5 presents the level of agreement between data and MC simulation in *W* and *Z* boson production events. Performance studies of the $E_{\text {T}}^{\text {miss}}$  algorithms on data and MC simulation are shown for samples with different event topologies in Sect. 6. The choice of jet selection criteria used in the $E_{\text {T}}^{\text {miss}}$ reconstruction is discussed in Sect. 7. Finally, the systematic uncertainty in the absolute scale and resolution of the $E_{\text {T}}^{\text {miss}}$  is discussed in Sect. 8. To provide a reference, Table 1 summarizes the different $E_{\text {T}}^{\text {miss}}$ terms discussed in this paper.

**Table 1:** Summary of definitions for $E_{\text {T}}^{\text {miss}}$  terms used in this paper

Term	Brief description
Intrinsic $E_{\text {T}}^{\text {miss}}$	Missing transverse momentum arising from the presence of neutrinos or other non-interacting particles in an event. In case of simulated events the true $E_{\text {T}}^{\text {miss}}$ ($E_\mathrm{T}^\mathrm{miss,True}$) corresponds to the $E_{\text {T}}^{\text {miss}}$ in such events defined as the magnitude of the vector sum of $\vec {p_{\text {T}}}$ of non-interacting particles computed from the generator information
Fake $E_{\text {T}}^{\text {miss}}$	Missing transverse momentum arising from the miscalibration or misidentification of physics objects in the event. It is typically studied in $Z \rightarrow \mu \mu$ events where the intrinsic $E_{\text {T}}^{\text {miss}}$ is normally expected to be zero
Hard terms	The component of the $E_{\text {T}}^{\text {miss}}$ computed from high-${p}_{\text {T}}$ physics objects, which includes reconstructed electrons, photons, muons, $\tau$-leptons, and jets
Soft terms	Typically low-$p_{\text {T}}$ calorimeter energy deposits or tracks, depending on the soft-term definition, that are not associated to physics objects included in the hard terms
Pileup-suppressed $E_{\text {T}}^{\text {miss}}$	All $E_{\text {T}}^{\text {miss}}$ reconstruction algorithms in Sect. 4.1.2 except the Calorimeter Soft Term, which does not apply pileup suppression
Object-based	This refers to all reconstruction algorithms in Sect. 4.1.2 except the Track $E_{\text {T}}^{\text {miss}}$ , namely the Calorimeter Soft Term, Track Soft Term, Extrapolated Jet Area with Filter, and Soft-Term Vertex-Fraction algorithms. These consider the physics objects such as electrons, photons, muons, $\tau$-leptons, and jets during the $E_{\text {T}}^{\text {miss}}$ reconstruction

## ATLAS detector

The ATLAS detector [2] is a multi-purpose particle physics apparatus with a forward-backward symmetric cylindrical geometry and nearly 4$\pi$ coverage in solid angle. For tracking, the inner detector (ID) covers the pseudorapidity range of $|\eta |$ < 2.5, and consists of a silicon-based pixel detector, a semiconductor tracker (SCT) based on microstrip technology, and, for $|\eta |$ < 2.0, a transition radiation tracker (TRT). The ID is surrounded by a thin superconducting solenoid providing a 2 T magnetic field, which allows the measurement of the momenta of charged particles. A high-granularity electromagnetic sampling calorimeter based on lead and liquid argon (LAr) technology covers the region of $|\eta |<3.2$. A hadronic calorimeter based on steel absorbers and plastic-scintillator tiles provides coverage for hadrons, jets, and $\tau$-leptons in the range of $|\eta |$ < 1.7. LAr technology using a copper absorber is also used for the hadronic calorimeters in the end-cap region of 1.5 < $|\eta |$ < 3.2 and for electromagnetic and hadronic measurements with copper and tungsten absorbing materials in the forward region of 3.1 < $|\eta |$ < 4.9. The muon spectrometer (MS) surrounds the calorimeters. It consists of three air-core superconducting toroid magnet systems, precision tracking chambers to provide accurate muon tracking out to $|\eta |$ $=$ 2.7, and additional detectors for triggering in the region of $|\eta |$ < 2.4. A precision measurement of the track coordinates is provided by layers of drift tubes at three radial positions within $|\eta |$ < 2.0. For 2.0 < $|\eta |$ < 2.7, cathode-strip chambers with high granularity are instead used in the innermost plane. The muon trigger system consists of resistive-plate chambers in the barrel ($|\eta |$ < 1.05) and thin-gap chambers in the end-cap regions (1.05 < $|\eta |$ < 2.4).

## Data samples and event selection

ATLAS recorded *pp* collisions at a centre-of-mass energy of 8 $$\text {TeV}$$ with a bunch crossing interval (bunch spacing) of $$50\,\mathrm{ns}$$ in 2012. The resulting integrated luminosity is 20.3 $$\mathrm{fb}^{-1}$$ [3]. Multiple inelastic $$pp $$ interactions occurred in each bunch crossing, and the mean number of inelastic collisions per bunch crossing ($$\langle \mu \rangle $$) over the full dataset is 21 [4], exceptionally reaching as high as about 70.

Data are analysed only if they satisfy the standard ATLAS data-quality assessment criteria [5]. Jet-cleaning cuts [5] are applied to minimize the impact of instrumental noise and out-of-time energy deposits in the calorimeter from cosmic rays or beam-induced backgrounds. This ensures that the residual sources of $E_{\mathrm {T}}^{\mathrm {miss}}$ mismeasurement due to those instrumental effects are suppressed.

### Track and vertex selection

The ATLAS detector measures the momenta of charged particles using the ID [6]. Hits from charged particles are recorded and are used to reconstruct tracks; these are used to reconstruct vertices [7, 8].

Each vertex must have at least two tracks with ${p}_{\text {T}}$ > 0.4 $\text {GeV}$; for the primary hard-scatter vertex (PV), the requirement on the number of tracks is raised to three. The PV in each event is selected as the vertex with the largest value of $\Sigma \,({p}_{\text {T}})^2$, where the scalar sum is taken over all the tracks matched to the vertex. The following track selection criteria^3^ [7] are used throughout this paper, including the vertex reconstruction:

- ${p}_{\text {T}}$  > 0.5 $\text {GeV}$ (0.4 $\text {GeV}$ for vertex reconstruction and the calorimeter soft term),
- $$|\eta |$$ < 2.5,
- Number of hits in the pixel detector $\ge$ 1,
- Number of hits in the SCT $\ge$ 6.

These tracks are then matched to the PV by applying the following selections:

- $$|d_0|$$ < 1.5 mm,
- $$|z_0\sin (\theta $$)| < 1.5 mm.

The transverse (longitudinal) impact parameter $d_0$ $(z_0)$ is the transverse (longitudinal) distance of the track from the PV and is computed at the point of closest approach to the PV in the plane transverse to the beam axis. The requirements on the number of hits ensures that the track has an accurate ${p}_{\text {T}}$  measurement. The $|\eta |$ requirement keeps only the tracks within the ID acceptance, and the requirement of ${p}_{\text {T}}$  > 0.4 $\text {GeV}$ ensures that the track reaches the outer layers of the ID. Tracks with low ${p}_{\text {T}}$  have large curvature and are more susceptible to multiple scattering.

The average spread along the beamline direction for *pp* collisions in ATLAS during 2012 data taking is around 50 mm, and the typical track $z_0$ resolution for those with $|\eta |~<~0.2$ and $0.5~<~p_{\text {T}} ~<~0.6$ $\text {GeV}$ is 0.34 mm. The typical track $d_0$ resolution is around 0.19 mm for the same $\eta$ and $p_{\text {T}}$ ranges, and both the $z_0$ and $d_0$ resolutions improve with higher track ${p}_{\text {T}}$ .

Pileup effects come from two sources: in-time and out-of-time. In-time pileup is the result of multiple *pp* interactions in the same LHC bunch crossing. It is possible to distinguish the in-time pileup interactions by using their vertex positions, which are spread along the beam axis. At $\langle \mu \rangle$ $=$ 21, the efficiency to reconstruct and select the correct vertex for $\mathrm{Z} \rightarrow \mu {}\mu$  simulated events is around 93.5% and rises to more than 98% when requiring two generated muons with ${p}_{\text {T}}$  > 10 $\text {GeV}$ inside the ID acceptance [10]. When vertices are separated along the beam axis by a distance smaller than the position resolution, they can be reconstructed as a single vertex. Each track in the reconstructed vertex is assigned a weight based upon its compatibility with the fitted vertex, which depends on the $\chi ^2$ of the fit. The fraction of $\mathrm{Z} \rightarrow \mu {}\mu$  reconstructed vertices with more than 50% of the sum of track weights coming from pileup interactions is around 3% at $\langle \mu \rangle$ $=$ 21 [7, 10]. Out-of-time pileup comes from *pp* collisions in earlier and later bunch crossings, which leave signals in the calorimeters that can take up to 450 ns for the charge collection time. This is longer than the 50 ns between subsequent collisions and occurs because the integration time of the calorimeters is significantly larger than the time between the bunch crossings. By contrast the charge collection time of the silicon tracker is less than 25 ns.

### Event selection for $\mathrm{Z} \rightarrow \ell{}\ell$

The “standard candle” for evaluation of the $E_{\mathrm {T}}^{\mathrm {miss}}$ performance is $\mathrm{Z} \rightarrow \ell{}\ell$  events ($\ell =e$ or $\mu$). They are produced without neutrinos, apart from a very small number originating from heavy-flavour decays in jets produced in association with the *Z* boson. The intrinsic $E_{\mathrm {T}}^{\mathrm {miss}}$ is therefore expected to be close to zero, and the $E_{\mathrm {T}}^{\mathrm {miss}}$ distributions are used to evaluate the modelling of the effects that give rise to fake $E_{\text {T}}^{\text {miss}}$ .

Candidate $\mathrm{Z} \rightarrow \ell{}\ell$ events are required to pass an electron or muon trigger [11, 12]. The lowest ${p}_{\text {T}}$  threshold for the unprescaled single-electron (single-muon) trigger is $p_{\text {T}}$  > 25 (24) $\text {GeV}$, and both triggers apply a track-based isolation as well as quality selection criteria for the particle identification. Triggers with higher ${p}_{\text {T}}$  thresholds, without the isolation requirements, are used to improve acceptance at high ${p}_{\text {T}}$ . These triggers require $p_{\text {T}}$  > 60 (36) $\text {GeV}$ for electrons (muons). Events are accepted if they pass any of the above trigger criteria. Each event must contain at least one primary vertex with a *z* displacement from the nominal *pp* interaction point of less than $200\,\mathrm{mm}$ and with at least three associated tracks.

The offline selection of $\mathrm{Z} \rightarrow \mu {}\mu$ events requires the presence of exactly two identified muons [13]. An identified muon is reconstructed in the MS and is matched to a track in the ID. The combined ID$+$MS track must have ${p}_{\text {T}}$  > 25 $\text {GeV}$ and $|\eta |$ < 2.5. The *z* displacement of the muon track from the primary vertex is required to be less than 10 mm. An isolation criterion is applied to the muon track, where the scalar sum of the ${p}_{\text {T}}$  of additional tracks within a cone of size $\Delta R$ $=$ $\sqrt{(\Delta \eta )^2+(\Delta \phi )^2}$ $=$ 0.2 around the muon is required to be less than 10% of the muon $p_{\text {T}}$ . In addition, the two leptons are required to have opposite charge, and the reconstructed dilepton invariant mass, $m_{\ell \ell }$, is required to be consistent with the *Z* boson mass: 66 < $m_{\ell \ell }$ < 116 $\text {GeV}$.

The $E_{\text {T}}^{\text {miss}}$ modelling and performance results obtained in $\mathrm{Z} \rightarrow \mu {}\mu$ and $Z\rightarrow e e$ events are very similar. For the sake of brevity, only the $\mathrm{Z} \rightarrow \mu {}\mu$ distributions are shown in all sections except for Sect. 6.6.

### Event selection for $W\rightarrow \ell {}\nu$

Leptonically decaying *W* bosons ($W\rightarrow \ell {}\nu$) provide an important event topology with intrinsic $E_{\text {T}}^{\text {miss}}$; the $E_{\text {T}}^{\text {miss}}$ distribution for such events is presented in Sect. 5.2. Similar to $\mathrm{Z} \rightarrow \ell{}\ell$ events, a sample dominated by leptonically decaying *W* bosons is used to study the $E_{\mathrm {T}}^{\mathrm {miss}}$ scale in Sect. 6.2.2, the resolution of the $E_{\text {T}}^{\text {miss}}$  direction in Sect. 6.3, and the impact on a reconstructed kinematic observable in Sect. 6.4.

The $E_{\text {T}}^{\text {miss}}$  distributions for *W* boson events in Sect. 5.2 use the electron final state. These electrons are selected with $|\eta |$ < 2.47, are required to meet the “medium” identification criteria [14] and satisfy ${p}_{\text {T}}$  > 25 $\text {GeV}$. Electron candidates in the region 1.37 < $|\eta |$ < 1.52 suffer from degraded momentum resolution and particle identification due to the transition from the barrel to the end-cap detector and are therefore discarded in these studies. The electrons are required to be isolated, such that the sum of the energy in the calorimeter within a cone of size $\Delta R$ $=$ 0.3 around the electron is less than 14% of the electron ${p}_{\text {T}}$ . The summed ${p}_{\text {T}}$ of other tracks within the same cone is required to be less than 7% of the electron ${p}_{\text {T}}$ . The calorimeter isolation variable [14] is corrected by subtracting estimated contributions from the electron itself, the underlying event [15], and pileup. The electron tracks are then matched to the PV by applying the following selections:

- $$|d_0|$$ < 5.0 mm,
- $$|z_0\sin (\theta $$)| < 0.5 mm.

The *W* boson selection is based on the single-lepton triggers and the same lepton selection criteria as those used in the $\mathrm{Z} \rightarrow \ell{}\ell$  selection. Events are rejected if they contain more than one reconstructed lepton. Selections on the $E_{\mathrm {T}}^{\mathrm {miss}}$ and transverse mass ($m_{\mathrm {T}}$) are applied to reduce the multi-jet background with one jet misidentified as an isolated lepton. The transverse mass is calculated from the lepton and the $\vec {E}_{{\mathrm{T}}}^{\mathrm{miss}}$,

1 $$\begin{aligned} m_{\mathrm {T}}= \sqrt{2p_{\mathrm T} ^{\ell } E_{\mathrm {T}}^{\mathrm {miss}}(1-\cos \Delta \phi )}, \end{aligned}$$

where $p_{\mathrm T}^{\ell }$ is the transverse momentum of the lepton and $\Delta \phi$ is the azimuthal angle between the lepton and $\vec {E}_{{\mathrm{T}}}^{\mathrm{miss}}$ directions. Both the $m_{\mathrm {T}}$ and $E_{\text {T}}^{\text {miss}}$ are required to be greater than 50 $\text {GeV}$. These selections can bias the event topology and its phase space, so they are only used when comparing simulation to data in Sect. 5.2, as they substantially improve the purity of *W* bosons in data events.

The $E_{\text {T}}^{\text {miss}}$ modelling and performance results obtained in $W\rightarrow e{}v$ and $W\rightarrow \mu {}v$ events are very similar. For the sake of brevity, only one of the two is considered in following two sections: $E_{\text {T}}^{\text {miss}}$ distributions in $W\rightarrow e{}v$ events are presented in Sect. 5.2 and the performance studies show $W\rightarrow \mu {}v$ events in Sect. 6. When studying the $E_{\text {T}}^{\text {miss}}$ tails, both final states are considered in Sect. 6.6, because the $\eta$-coverage and reconstruction performance between muons and electrons differ.

### Monte Carlo simulation samples

Table 2 summarizes the MC simulation samples used in this paper. The $\mathrm{Z} \rightarrow \ell{}\ell$ and $W\rightarrow \ell {}\nu$  samples are generated with Alpgen [16] interfaced with Pythia  [17] (denoted by Alpgen $+$ Pythia) to model the parton shower and hadronization, and underlying event using the PERUGIA2011C set [18] of tunable parameters. One exception is the $Z \rightarrow \tau \tau$  sample with leptonically decaying $\tau$-leptons, which is generated with Alpgen interfaced with Herwig  [19] with the underlying event modelled using Jimmy [20] and the AUET2 tunes [21]. Alpgen is a multi-leg generator that provides tree-level calculations for diagrams with up to five additional partons. The matrix-element MC calculations are matched to a model of the parton shower, underlying event and hadronization. The main processes that are backgrounds to $\mathrm{Z} \rightarrow \ell{}\ell$  and $W\rightarrow \ell {}\nu$  are events with one or more top quarks ($t\bar{t}$  and single-top-quark processes) and diboson production (*WW*, *WZ*, *ZZ*). The $t\bar{t}$  and *tW* processes are generated with Powheg [22] interfaced with Pythia  [17] for hadronization and parton showering, and PERUGIA2011C for the underlying event modelling. All the diboson processes are generated with Sherpa  [23]. Powheg is a leading-order generator with corrections at next-to-leading order in $\alpha _{\text {S}}$, whereas Sherpa is a multi-leg generator at tree level.

To study event topologies with high jet multiplicities and to investigate the tails of the $E_{\mathrm {T}}^{\mathrm {miss}}$ distributions, $t\bar{t}$  events with at least one leptonically decaying *W* boson are considered in Sect. 6.6. The single top quark (*tW*) production is considered with at least one leptonically decaying *W* boson. Both the $t\bar{t}$  and *tW* processes contribute to the *W* and *Z* boson distributions shown in Sect. 5 as well as *Z* boson distributions in Sects. 4, 6, and 8 that compare data and simulation. A supersymmetric (SUSY) model comprising pair-produced 500 GeV gluinos each decaying to a $t\bar{t}$ pair and a neutralino is simulated with Herwig $++$ [24]. Finally, to study events with forward jets, the vector-boson fusion (VBF) production of $H \rightarrow \tau \tau$ , generated with Powheg $+$Pythia8 [25], is considered. Both $\tau$-leptons are forced to decay leptonically in this sample.

**Table 2:** Generators, cross-section normalizations, PDF sets, and MC tunes used in this analysis

Sample	Generator	Use	Cross-section	PDF set	Tune
$\mathrm{Z} \rightarrow \mu {}\mu$	Alpgen $+$ Pythia	Signal	NNLO [26]	CTEQ6L1 [27]	PERUGIA2011C [18]
$Z\rightarrow e e$	Alpgen $+$ Pythia	Signal	NNLO [26]	CTEQ6L1	PERUGIA2011C
$Z \rightarrow \tau \tau$	Alpgen $+$ Herwig	Signal	NNLO [26]	CTEQ6L1	AUET2 [21]
$W\rightarrow \mu {}v$	Alpgen $+$ Pythia	Signal	NNLO [26]	CTEQ6L1	PERUGIA2011C
$W\rightarrow e{}v$	Alpgen $+$ Pythia	Signal	NNLO [26]	CTEQ6L1	PERUGIA2011C
$W\rightarrow \tau {}v$	Alpgen $+$ Pythia	Signal	NNLO [26]	CTEQ6L1	PERUGIA2011C
$t\bar{t}$	Powheg $+$ Pythia	Signal/background	NNLO+NNLL [28, 29]	CTEQ6L1	PERUGIA2011C
VBF $H \rightarrow \tau \tau$	Powheg $+$Pythia8	Signal	–	NLO CT10 [30]	AU2 [31]
SUSY 500	Herwig $++$	Signal	–	CTEQ6L1	UE EE3 [32]
$W^{\pm }Z\rightarrow \ell ^{\pm }\nu \ell ^+\ell ^-$	Sherpa	Background	NLO [33, 34]	NLO CT10	Sherpa default
$ZZ\rightarrow \ell ^+\ell ^-\nu \bar{\nu }$	Sherpa	Background	NLO [33, 34]	NLO CT10	Sherpa default
$W^+W^-\rightarrow \ell ^+\nu \ell ^-\bar{\nu }$	Sherpa	Background	NLO [33, 34]	NLO CT10	Sherpa default
*tW*	Powheg $+$ Pythia	Background	NNLO+NNLL [35]	CTEQ6L1	PERUGIA2011C
$\mathrm{Z} \rightarrow \mu {}\mu$	Powheg $+$Pythia8	Systematic effects	NNLO [36, 37]	NLO CT10	AU2
$\mathrm{Z} \rightarrow \mu {}\mu$	Alpgen $+$ Herwig	Systematic effects	NNLO [36, 37]	CTEQ6L1	AUET2
$\mathrm{Z} \rightarrow \mu {}\mu$	Sherpa	Systematic effects	NNLO [36, 37]	NLO CT10	Sherpa default

To estimate the systematic uncertainties in the data/MC ratio arising from the modelling of the soft hadronic recoil, $E_{\text {T}}^{\text {miss}}$ distributions simulated with different MC generators, parton shower and underlying event models are compared. The estimation of systematic uncertainties is performed using a comparison of data and MC simulation, as shown in Sect. 8.2. The following combinations of generators and parton shower models are considered: Sherpa, Alpgen $+$ Herwig , Alpgen $+$ Pythia , and Powheg $+$Pythia8. The corresponding underlying event tunes are mentioned in Table 2. Parton distribution functions are taken from CT10 [30] for Powheg and Sherpa  samples and CTEQ6L1 [38] for Alpgen samples.

Generated events are propagated through a Geant4 simulation [39, 40] of the ATLAS detector. Pileup collisions are generated with Pythia8 for all samples, and are overlaid on top of simulated hard-scatter events before event reconstruction. Each simulation sample is weighted by its corresponding cross-section and normalized to the integrated luminosity of the data.

## Reconstruction and calibration of the $E_{\text {T}}^{\text {miss}}$

Several algorithms have been developed to reconstruct the $E_{\text {T}}^{\text {miss}}$  in ATLAS. They differ in the information used to reconstruct the $p_{\text {T}}$ of the particles, using either energy deposits in the calorimeters, tracks reconstructed in the ID, or both. This section describes these various reconstruction algorithms, and the remaining sections discuss the agreement between data and MC simulation as well as performance studies.

### Reconstruction of the $E_{\text {T}}^{\text {miss}}$

The $E_{\text {T}}^{\text {miss}}$  reconstruction uses calibrated physics objects to estimate the amount of missing transverse momentum in the detector. The $E_{\text {T}}^{\text {miss}}$ is calculated using the components along the *x* and *y* axes:

2 $$\begin{aligned} E_{{x(y)}}^{\mathrm {miss}} = E_{{x(y)}}^{\mathrm {miss},e} + E_{{x(y)}}^{\mathrm {miss},\gamma } + E_{{x(y)}}^{\mathrm {miss},\tau } + E_{{x(y)}}^{\mathrm {miss,jets}} + E_{{x(y)}}^{\mathrm {miss},\mu } + E_{{x(y)}}^{\mathrm {miss,soft}} , \end{aligned}$$

where each term is calculated as the negative vectorial sum of transverse momenta of energy deposits and/or tracks. To avoid double counting, energy deposits in the calorimeters and tracks are matched to reconstructed physics objects in the following order: electrons (*e*), photons ($\gamma$), the visible parts of hadronically decaying $\tau$-leptons ($\tau _{\mathrm{had}{\text {-}}\mathrm{vis}}$; labelled as $\tau$), jets and muons ($\mu$). Each type of physics object is represented by a separate term in Eq. (2). The signals not associated with physics objects form the “soft term”, whereas those associated with the physics objects are collectively referred to as the “hard term”.

The magnitude and azimuthal angle^4^ ($\phi ^\mathrm{miss}$) of $\vec {E}_{{\mathrm{T}}}^{\mathrm{miss}}$ are calculated as:

3 $$\begin{aligned} E_{\mathrm {T}}^{\mathrm {miss}}= & {} \sqrt{(E_{{x}} ^{\mathrm {miss}})^{2} +(E_{{y}}^{\mathrm {miss}})^{2}}, \nonumber \\ \phi ^\mathrm{miss}= & {} \text {arctan}(E_{{y}}^{\mathrm {miss}}/E_{{x}}^{\mathrm {miss}}). \end{aligned}$$

The total transverse energy in the detector, labelled as $\Sigma E_{\mathrm {T}}$, quantifies the total event activity and is an important observable for understanding the resolution of the $E_{\text {T}}^{\text {miss}}$ , especially with increasing pileup contributions. It is defined as:

4 $$\begin{aligned} \sum E_{\mathrm {T}} = \sum p_{\mathrm {T}}^{e} + \sum p_{\mathrm {T}}^{\gamma } + \sum p_{\mathrm {T}}^{\tau } + \sum p_{\mathrm {T}}^{\mathrm {jets}} + \sum p_{\mathrm {T}}^{\mu } + \sum p_{\mathrm {T}}^{\mathrm {soft}}, \end{aligned}$$

which is the scalar sum of the transverse momenta of reconstructed physics objects and soft-term signals that contribute to the $E_{\text {T}}^{\text {miss}}$ reconstruction. The physics objects included in $\sum p_{\mathrm {T}}^{\mathrm {soft}}$ depend on the $E_{\text {T}}^{\text {miss}}$  definition, so both calorimeter objects and track-based objects may be included in the sum, despite differences in ${p}_{\text {T}}$  resolution.

#### Reconstruction and calibration of the $E_{\mathrm {T}}^{\mathrm {miss}}$ hard terms

The hard term of the $E_{\text {T}}^{\text {miss}}$ , which is computed from the reconstructed electrons, photons, muons, $\tau$-leptons, and jets, is described in more detail in this section.

Electrons are reconstructed from clusters in the electromagnetic (EM) calorimeter which are associated with an ID track [14]. Electron identification is restricted to the range of $|\eta |$ < 2.47, excluding the transition region between the barrel and end-cap EM calorimeters, 1.37 < $|\eta |$ < 1.52. They are calibrated at the EM scale^5^ with the default electron calibration, and those satisfying the “medium” selection criteria [14] with $p_{\text {T}} >10$ $\text {GeV}$ are included in the $E_{\text {T}}^{\text {miss}}$ reconstruction.

The photon reconstruction is also seeded from clusters of energy deposited in the EM calorimeter and is designed to separate electrons from photons. Photons are calibrated at the EM scale and are required to satisfy the “tight” photon selection criteria with $p_{\text {T}}$  > 10 $\text {GeV}$ [14].

Muon candidates are identified by matching an ID track with an MS track or segment [13]. MS tracks are used for 2.5 < $|\eta |$ < 2.7 to extend the $\eta$ coverage. Muons are required to satisfy ${p}_{\text {T}}$ > 5 $\text {GeV}$ to be included in the $E_{\text {T}}^{\text {miss}}$ reconstruction. The contribution of muon energy deposited in the calorimeter is taken into account using either parameterized estimates or direct measurements, to avoid double counting a small fraction of their momenta.

Jets are reconstructed from three-dimensional topological clusters (topoclusters) [41] of energy deposits in the calorimeter using the anti-$k_t$ algorithm [42] with a distance parameter *R* $=$ 0.4. The topological clustering algorithm suppresses noise by forming contiguous clusters of calorimeter cells with significant energy deposits. The local cluster weighting (LCW) [43, 44] calibration is used to account for different calorimeter responses to electrons, photons and hadrons. Each cluster is classified as coming from an EM or hadronic shower, using information from its shape and energy density, and calibrated accordingly. The jets are reconstructed from calibrated topoclusters and then corrected for in-time and out-of-time pileup as well as the position of the PV [4]. Finally, the jet energy scale (JES) corrects for jet-level effects by restoring, on average, the energy of reconstructed jets to that of the MC generator-level jets. The complete procedure is referred to as the LCW+JES scheme [43, 44]. Without changing the average calibration, additional corrections are made based upon the internal properties of the jet (global sequential calibration) to reduce the flavour dependence and energy leakage effects [44]. Only jets with calibrated ${p}_{\text {T}}$  greater than 20 $\text {GeV}$ are used to calculate the jet term $E_{{x(y)}}^{\mathrm {miss,jets}}$ in Eq. (2), and the optimization of the 20 $\text {GeV}$ threshold is discussed in Sect. 7.

To suppress contributions from jets originating from pileup interactions, a requirement on the jet vertex-fraction (JVF) [4] may be applied to selected jet candidates. Tracks matched to jets are extrapolated back to the beamline to ascertain whether they originate from the hard scatter or from a pileup collision. The JVF is then computed as the ratio shown below:

5 $$\begin{aligned} {\mathrm {JVF}} = \sum _{\mathrm {track,PV,jet}} p_{\mathrm {T}}{{{/}}}\sum _{\mathrm {track,jet}} p_{\mathrm {T}}. \end{aligned}$$

This is the ratio of the scalar sum of transverse momentum of all tracks matched to the jet and the primary vertex to the ${p}_{\text {T}}$  sum of all tracks matched to the jet, where the sum is performed over all tracks with $p_{\text {T}}$  > 0.5 $\text {GeV}$ and $|\eta |$ < 2.5 and the matching is performed using the “ghost-association” procedure [45, 46].

The JVF distribution is peaked toward 1 for hard-scatter jets and toward 0 for pileup jets. No JVF selection requirement is applied to jets that have no associated tracks. Requirements on the JVF are made in the STVF, EJAF, and TST $E_{\text {T}}^{\text {miss}}$  algorithms as described in Table 3 and Sect. 4.1.3.

Hadronically decaying $\tau$-leptons are seeded by calorimeter jets with $|\eta |$ < 2.5 and $p_{\text {T}}$  > 10 $\text {GeV}$. As described for jets, the LCW calibration is applied, corrections are made to subtract the energy due to pileup interactions, and the energy of the hadronically decaying $\tau$ candidates is calibrated at the $\tau$-lepton energy scale (TES) [47]. The TES is independent of the JES and is determined using an MC-based procedure. Hadronically decaying $\tau$-leptons passing the “medium” requirements [47] and having $p_{\text {T}}$  > 20 $\text {GeV}$ after TES corrections are considered for the $E_{\text {T}}^{\text {miss}}$ reconstruction.

#### Reconstruction and calibration of the $E_{\mathrm {T}}^{\mathrm {miss}}$ soft term

The soft term is a necessary but challenging ingredient of the $E_{\text {T}}^{\text {miss}}$ reconstruction. It comprises all the detector signals not matched to the physics objects defined above and can contain contributions from the hard scatter as well as the underlying event and pileup interactions. Several algorithms designed to reconstruct and calibrate the soft term have been developed, as well as methods to suppress the pileup contributions. A summary of the $E_{\text {T}}^{\text {miss}}$ and soft-term reconstruction algorithms is given in Table 3.

**Table 3:** Summary of $E_{\text {T}}^{\text {miss}}$ and soft-term reconstruction algorithms used in this paper

Term	Brief description	Section list
CST $E_{\text {T}}^{\text {miss}}$	The Calorimeter Soft Term (CST) $E_{\text {T}}^{\text {miss}}$ takes its soft term from energy deposits in the calorimeter which are not matched to high-${p}_{\text {T}}$ physics objects. Although noise suppression is applied to reduce fake signals, no additional pileup suppression techniques are used	
		Section 4.1.2 (definition)
		Section 5.1 ($Z \rightarrow \mu \mu$ modelling)
		Section 5.2 ($W\rightarrow e{}v$ modelling)
		Section 6 (perf. studies)
TST $E_{\text {T}}^{\text {miss}}$	The Track Soft Term (TST) $E_{\text {T}}^{\text {miss}}$ algorithm uses a soft term that is calculated using tracks within the inner detector that are not associated with high-${p}_{\text {T}}$ physics objects. The JVF selection requirement is applied to jets	
		Section 4.1.2 (definition)
		Section 5.1 ($Z \rightarrow \mu \mu$ modelling)
		Section 5.2 ($W\rightarrow e{}v$ modelling)
		Section 6 (perf. studies)
EJAF $E_{\text {T}}^{\text {miss}}$	The Extrapolated Jet Area with Filter $E_{\text {T}}^{\text {miss}}$ algorithm applies pileup subtraction to the CST based on the idea of jet-area corrections. The JVF selection requirement is applied to jets	
		Section 4.1.2 (definition)
		Section 5.1 ($Z \rightarrow \mu \mu$ modelling)
		Section 6 (perf. studies)
STVF $E_{\text {T}}^{\text {miss}}$	The Soft-Term Vertex-Fraction (STVF) $E_{\text {T}}^{\text {miss}}$ algorithm suppresses pileup effects in the CST by scaling the soft term by a multiplicative factor calculated based on the fraction of scalar-summed track $p_{\text {T}}$ not associated with high-${p}_{\text {T}}$ physics objects that can be matched to the primary vertex. The JVF selection requirement is applied to jets	
		Section 4.1.2 (definition)
		Section 5.1 ($Z \rightarrow \mu \mu$ modelling)
		Section 6 (perf. studies)
Track $E_{\text {T}}^{\text {miss}}$	The Track $E_{\text {T}}^{\text {miss}}$ is reconstructed entirely from tracks to avoid pileup contamination that affects the other algorithms	
		Section 4.2 (definition)
		Section 5.1 ($Z \rightarrow \mu \mu$ modelling)
		Section 6 (perf. studies)

Four soft-term reconstruction algorithms are considered in this paper. Below the first two are defined, and then some motivation is given for the remaining two prior to their definition.

- Calorimeter Soft Term (CST) This reconstruction algorithm [1] uses information mainly from the calorimeter and is widely used by ATLAS. The algorithm also includes corrections based on tracks but does not attempt to resolve the various *pp* interactions based on the track $z_0$ measurement. The soft term is referred to as the CST, whereas the entire $E_{\text {T}}^{\text {miss}}$ is written as CST $E_{\text {T}}^{\text {miss}}$ . Corresponding naming schemes are used for the other reconstruction algorithms. The CST is reconstructed using energy deposits in the calorimeter which are not matched to the high-${p}_{\text {T}}$ physics objects used in the $E_{\text {T}}^{\text {miss}}$ . To avoid fake signals in the calorimeter, noise suppression is important. This is achieved by calculating the soft term using only cells belonging to topoclusters, which are calibrated at the LCW scale [43, 44]. The tracker and calorimeter provide redundant ${p}_{\text {T}}$ measurements for charged particles, so an energy-flow algorithm is used to determine which measurement to use. Tracks with ${p}_{\text {T}}$  > 0.4 $\text {GeV}$ that are not matched to a high-${p}_{\text {T}}$  physics objects are used instead of the calorimeter ${p}_{\text {T}}$  measurement, if their ${p}_{\text {T}}$  resolution is better than the expected calorimeter $p_{\text {T}}$  resolution. The calorimeter resolution is estimated as $0.4\cdot \sqrt{p_{\text {T}}}~\text {GeV}{}$, in which the $p_{\text {T}}$  is the transverse momentum of the reconstructed track. Geometrical matching between tracks and topoclusters (or high-${p}_{\text {T}}$  physics objects) is performed using the $\Delta R$ significance defined as $\Delta R / \sigma _{\Delta R}$, where $\sigma _{\Delta R}$ is the $\Delta R$ resolution, parameterized as a function of the track ${p}_{\text {T}}$ . A track is considered to be associated to a topocluster in the soft term when its minimum $\Delta R / \sigma _{\Delta R}$ is less than 4. To veto tracks matched to high-${p}_{\text {T}}$  physics objects, tracks are required to have $\Delta R / \sigma _{\Delta R}$ > 8. The $E_{\mathrm {T}}^{\mathrm {miss}}$ calculated using the CST algorithm is documented in previous publications such as Ref. [1] and is the standard algorithm in most ATLAS 8 $\text {TeV}$ analyses.
- Track Soft Term (TST) The TST is reconstructed purely from tracks that pass the selections outlined in Sect. 3.1 and are not associated with the high-${p}_{\text {T}}$ physics objects defined in Sect. 4.1.1. The detector coverage of the TST is the ID tracking volume ($|\eta |$ < 2.5), and no calorimeter topoclusters inside or beyond this region are included. This algorithm allows excellent vertex matching for the soft term, which almost completely removes the in-time pileup dependence, but misses contributions from soft neutral particles. The track-based reconstruction also entirely removes the out-of-time pileup contributions that affect the CST. To avoid double counting the ${p}_{\text {T}}$ of particles, the tracks matched to the high-${p}_{\text {T}}$ physics objects need to be removed from the soft term. All of the following classes of tracks are excluded from the soft term:
  - tracks within a cone of size $\Delta R$ $=$ 0.05 around electrons and photons
  - tracks within a cone of size $\Delta R$ $=$ 0.2 around $\tau _{\mathrm{had}{\text {-}}\mathrm{vis}}$
  - ID tracks associated with identified muons
  - tracks matched to jets using the ghost-association technique described in Sect. 4.1.1
  - isolated tracks with ${p}_{\text {T}} ~\ge ~120$ $\text {GeV}$ ($\ge$200 $\text {GeV}$ for $|\eta |$ < 1.5) having transverse momentum uncertainties larger than 40% or having no associated calorimeter energy deposit with $p_{\text {T}}$  larger than 65% of the track ${p}_{\text {T}}$ . The ${p}_{\text {T}}$  thresholds are chosen to ensure that muons not in the coverage of the MS are still included in the soft term. This is a cleaning cut to remove mismeasured tracks.

A deterioration of the CST $E_{\text {T}}^{\text {miss}}$  resolution is observed as the average number of pileup interactions increases [1]. All $E_{\text {T}}^{\text {miss}}$  terms in Eq. (2) are affected by pileup, but the terms which are most affected are the jet term and CST, because their constituents are spread over larger regions in the calorimeters than those of the $E_{\text {T}}^{\text {miss}}$  hard terms. Methods to suppress pileup are therefore needed, which can restore the $E_{\mathrm {T}}^{\mathrm {miss}}$ resolution to values similar to those observed in the absence of pileup.

**Fig. 1:**
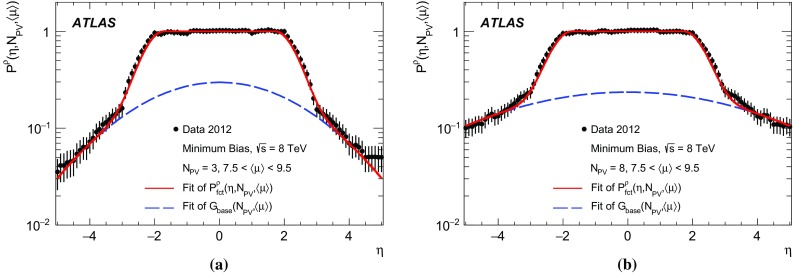
The average transverse momentum density shape $P^\rho (\eta ,N_{\text {PV}},$ $\langle \mu \rangle$) for jets in data is compared to the model in Eq. (9) with $\langle \mu \rangle$ $=$ 7.5–9.5 and with **a** three reconstructed vertices and **b** eight reconstructed vertices. The increase of jet activity in the forward regions coming from more in-time pileup with ${N}_{\mathrm {PV}}$ $=$ 8 in **b** can be seen by the flatter shape of the Gaussian fit of the forward activity $G_{\mathrm {base}}($ ${N}_{\mathrm {PV}}$, $\langle \mu \rangle$) (*blue dashed line*)

**Fig. 2:**
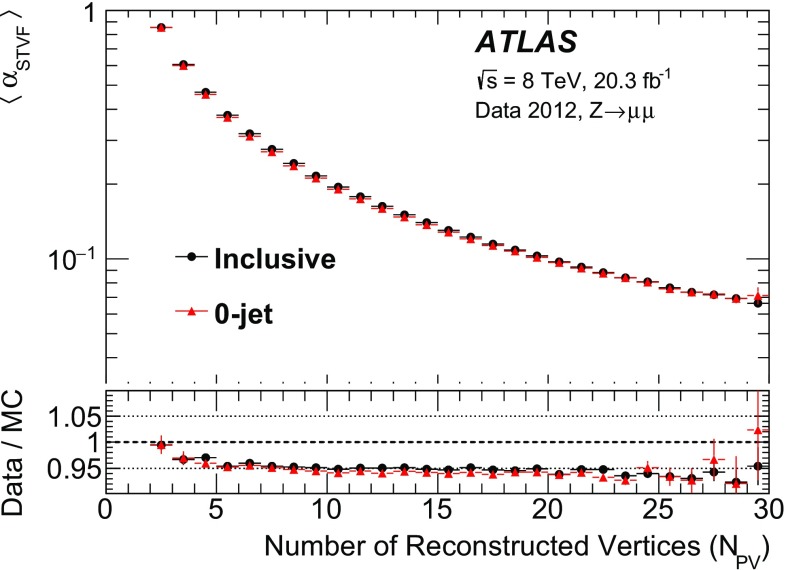
The mean $\alpha _{\text {STVF}}$  weight is shown versus the number of reconstructed vertices (${N}_{\mathrm {PV}}$) for 0-jet and inclusive events in $\mathrm{Z} \rightarrow \mu {}\mu$  data. The *inset* at the *bottom* of the figure shows the ratio of the data to the MC predictions with only the statistical uncertainties on the data and MC simulation. The bin boundary always includes the lower edge and not the upper edge

The TST algorithm is very stable with respect to pileup but does not include neutral particles. Two other pileup-suppressing algorithms were developed, which consider contributions from neutral particles. One uses an $\eta$-dependent event-by-event estimator for the transverse momentum density from pileup, using calorimeter information, while the other applies an event-by-event global correction based on the amount of charged-particle ${p}_{\text {T}}$ from the hard-scatter vertex, relative to all other *pp* collisions. The definitions of these two soft-term algorithms are described in the following:

- Extrapolated Jet Area with Filter (EJAF) The jet-area method for the pileup subtraction uses a soft term based on the idea of jet-area corrections [45]. This technique uses direct event-by-event measurements of the energy flow throughout the entire ATLAS detector to estimate the ${p}_{\text {T}}$  density of pileup energy deposits and was developed from the strategy applied to jets as described in Ref. [4]. The topoclusters belonging to the soft term are used for jet finding with the $k_{t}$ algorithm [48, 49] with distance parameter *R* $=$ 0.6 and jet $p_{\text {T}}$  > 0. The catchment areas [45, 46] for these reconstructed jets are labelled $A_{\mathrm {jet}}$; this provides a measure of the jet’s susceptibility to contamination from pileup. Jets with ${p}_{\text {T}}$  < 20 $\text {GeV}$ are referred to as soft-term jets, and the ${p}_{\text {T}}$-density of each soft-term jet *i* is then measured by computing:
  6 $$\begin{aligned} \rho _{\mathrm {jet}, i} = \frac{p_{\mathrm {T}, i}^{\mathrm {jet}}}{A_{\mathrm {jet}, i}} . \end{aligned}$$
  In a given event, the median ${p}_{\text {T}}$-density $\rho _{\mathrm {evt}}^{\mathrm {med}}$ for all soft-term $k_{t}$ jets in the event ($N_{\mathrm {jets}}$) found within a given range $-\eta _{\mathrm {max}}< \eta _{\mathrm {jet}}< \eta _{\mathrm {max}}$ can be calculated as
  7 $$\begin{aligned} \rho _{\mathrm {evt}}^{\mathrm {med}}= \mathrm {median}\{\rho _{\mathrm {jet,}i}\}\mathrm {\ for\ } i = 1\ldots N_{\mathrm {jets}}\mathrm {\ in\ } |\eta _{\mathrm {jet}}| < \eta _{\mathrm {max}}\,. \end{aligned}$$
  This median ${p}_{\text {T}}$-density $\rho _{\mathrm {evt}}^{\mathrm {med}}$ gives a good estimate of the in-time pileup activity in each detector region. If determined with $\eta _{\mathrm {max}}$ $=$ 2, it is found to also be an appropriate indicator of out-of-time pileup contributions [45]. A lower value for $\rho _{\mathrm {evt}}^{\mathrm {med}}$ is computed by using jets with $|\eta _{\mathrm {jet}}|$ larger than 2, which is mostly due to the particular geometry of the ATLAS calorimeters and their cluster reconstruction algorithms.^6^ In order to extrapolate $\rho _{\mathrm {evt}}^{\mathrm {med}}$ into the forward regions of the detector, the average topocluster $p_{\text {T}}$  in slices of $\eta$, ${N}_{\mathrm {PV}}$, and $\langle \mu \rangle$ is converted to an average $p_{\text {T}}$  density $\langle \rho \rangle (\eta ,{N}_{\mathrm {PV}}{}, \mu )$ for the soft term. As described for the $\rho _{\mathrm {evt}}^{\mathrm {med}}$, $\langle \rho \rangle (\eta ,{N}_{\mathrm {PV}}{}, \mu )$ is found to be uniform in the central region of the detector with $|\eta |$ < $\eta _\mathrm {plateau}$ $=$ 1.8. The transverse momentum density profile is then computed as
  8 $$\begin{aligned} P^\rho (\eta ,N_{\text {PV}},\langle \mu \rangle ) = \frac{\langle \rho \rangle (\eta ,{N}_{\mathrm {PV}}{}, \mu )}{\langle \rho \rangle _{\text {central}}({N}_{\mathrm {PV}}{}, \mu )} \end{aligned}$$
  where $\langle \rho \rangle _{\text {central}}({N}_{\mathrm {PV}}{}, \mu )$ is the average $\langle \rho \rangle (\eta ,{N}_{\mathrm {PV}}{}, \mu )$ for $|\eta |$ < $\eta _\mathrm {plateau}$. The $P^\rho (\eta ,N_{\text {PV}},$ $\langle \mu \rangle$) is therefore 1, by definition, for $|\eta |$ < $\eta _\mathrm {plateau}$ and decreases for larger $|\eta |$. A functional form of $P^\rho (\eta ,N_{\text {PV}},$ $\langle \mu \rangle$) is used to parameterize its dependence on $\eta$, ${N}_{\mathrm {PV}}$, and $\langle \mu \rangle$ and is defined as
  9 $$\begin{aligned} P_{\text {fct}}^\rho (\eta ,N_{\text {PV}},\langle \mu \rangle ) = \left\{ \begin{array}{ll} 1 &{} (|\eta |~<~\eta _\mathrm {plateau}) \\ (1 - G_\mathrm{{base}}(\eta _\mathrm {plateau})) \cdot G_\mathrm{{core}}(|\eta |-\eta _\mathrm {plateau}) + G_\mathrm{{base}}(\eta ) &{} \left( |\eta |~\ge ~\eta _\mathrm {plateau}\right) \end{array} \right. \end{aligned}$$
  where the central region $|\eta |$ < $\eta _\mathrm {plateau}$ $=$ 1.8 is plateaued at 1, and then a pair of Gaussian functions $G_\mathrm{{core}}(|\eta |-\eta _\mathrm {plateau})$ and $G_\mathrm{{base}}(\eta )$ are added for the fit in the forward regions of the calorimeter. The value of $G_\mathrm{{core}}(0)~=~1$ so that Eq. (9) is continuous at $\eta ~=~\eta _\mathrm {plateau}$. Two example fits are shown in Fig. 1 for ${N}_{\mathrm {PV}}$ $=$ 3 and 8 with $\langle \mu \rangle$ $=$ 7.5–9.5 interactions per bunch crossing. For both distributions the value is defined to be unity in the central region ($|\eta |$ < $\eta _\mathrm {plateau}$), and the sum of two Gaussian functions provides a good description of the change in the amount of in-time pileup beyond $\eta _\mathrm {plateau}$. The baseline Gaussian function $G_\mathrm{{base}}(\eta )$ has a larger width and is used to describe the larger amount of in-time pileup in the forward region as seen in Fig. 1. Fitting with Eq. (9) provides a parameterized function for in-time and out-of-time pileup which is valid for the whole 2012 dataset. The soft term for the EJAF $E_{\text {T}}^{\text {miss}}$  algorithm is calculated as
  10 $$\begin{aligned} E_{x(y)}^{\mathrm {miss,soft}}= - \sum _{i=0}^{N_{{\mathrm{filter}{\text {-}}\mathrm{jet}}}} p_{x(y),i}^{\mathrm {jet,corr}}, \end{aligned}$$
  which sums the transverse momenta, labelled $p_{x(y),i}^{\mathrm {jet,corr}}$, of the corrected soft-term jets matched to the primary vertex. The number of these filtered jets, which are selected after the pileup correction based on their JVF and ${p}_{\text {T}}$ , is labelled $N_{{\mathrm{filter}{\text {-}}\mathrm{jet}}}$. More details of the jet selection and the application of the pileup correction to the jets are given in Appendix A.
- Soft-Term Vertex-Fraction (STVF)
  The algorithm, called the soft-term vertex-fraction, utilizes an event-level parameter computed from the ID track information, which can be reliably matched to the hard-scatter collision, to suppress pileup effects in the CST. This correction is applied as a multiplicative factor ($\alpha _{\text {STVF}}$ ) to the CST, event by event, and the resulting STVF-corrected CST is simply referred to as STVF. The $\alpha _{\text {STVF}}$ is calculated as
  11 $$\begin{aligned} \alpha _{\text {STVF}} ={\sum _{\mathrm {tracks,PV}} p_{\mathrm {T}}}{{{/}}}\sum _{\mathrm {tracks}} p_{\mathrm {T}}, \end{aligned}$$
  which is the scalar sum of ${p}_{\text {T}}$  of tracks matched to the PV divided by the total scalar sum of track ${p}_{\text {T}}$  in the event, including pileup. The sums are taken over the tracks that do not match high-$p_{\text {T}}$ physics objects belonging to the hard term. The mean $\alpha _{\text {STVF}}$  value is shown versus the number of reconstructed vertices (${N}_{\mathrm {PV}}$) in Fig. 2. Data and simulation (including *Z*, diboson, $t\bar{t}$ , and *tW* samples) are shown with only statistical uncertainties and agree within 4–7% across the full range of ${N}_{\mathrm {PV}}$ in the 8 $\text {TeV}$ dataset. The differences mostly arise from the modelling of the amount of the underlying event and $p_{\mathrm {T}}^{Z}$. The 0-jet and inclusive samples have similar values of $\alpha _{\text {STVF}}$ , with that for the inclusive sample being around 2% larger.

#### Jet ${p}_{\text {T}}$  threshold and JVF selection

The TST, STVF, and EJAF $E_{\text {T}}^{\text {miss}}$  algorithms complement the pileup reduction in the soft term with additional requirements on the jets entering the $E_{\text {T}}^{\text {miss}}$  hard term, which are also aimed at reducing pileup dependence. These $E_{\text {T}}^{\text {miss}}$  reconstruction algorithms apply a requirement of $\text {JVF}$ > 0.25 to jets with ${p}_{\text {T}}$ < 50 $\text {GeV}$ and $|\eta |$ < 2.4 in order to suppress those originating from pileup interactions. The maximum $|\eta |$ value is lowered to 2.4 to ensure that the core of each jet is within the tracking volume ($|\eta |$ < 2.5) [4]. Charged particles from jets below the $p_{\text {T}}$  threshold are considered in the soft terms for the STVF, TST, and EJAF (see Sect. 4.1.2 for details).

The same $\text {JVF}$ requirements are not applied to the CST $E_{\text {T}}^{\text {miss}}$  because its soft term includes the soft recoil from all interactions, so removing jets not associated with the hard-scatter interaction could create an imbalance. The procedure for choosing the jet ${p}_{\text {T}}$ and $\text {JVF}$ criteria is summarized in Sect. 7.

Throughout most of this paper the number of jets is computed without a $\text {JVF}$ requirement so that the $E_{\text {T}}^{\text {miss}}$  algorithms are compared on the same subset of events. However, the $\text {JVF}$ > 0.25 requirement is applied in jet counting when 1-jet and $\ge$ 2-jet samples are studied using the TST $E_{\text {T}}^{\text {miss}}$ reconstruction, which includes Figs. 8 and 22. The $\text {JVF}$ removes pileup jets that obscure trends in samples with different jet multiplicities.

### Track $E_{\text {T}}^{\text {miss}}$

Extending the philosophy of the TST definition to the full event, the $E_{\text {T}}^{\text {miss}}$  is reconstructed from tracks alone, reducing the pileup contamination that afflicts the other object-based algorithms. While a purely track-based $E_{\text {T}}^{\text {miss}}$ , designated Track $E_{\text {T}}^{\text {miss}}$ , has almost no pileup dependence, it is insensitive to neutral particles, which do not form tracks in the ID. This can degrade the $E_{\text {T}}^{\text {miss}}$ calibration, especially in event topologies with numerous or highly energetic jets. The $\eta$ coverage of the Track $E_{\text {T}}^{\text {miss}}$  is also limited to the ID acceptance of $|\eta |$ < 2.5, which is substantially smaller than the calorimeter coverage, which extends to $|\eta |$ $=$ 4.9.

Track $E_{\text {T}}^{\text {miss}}$ is calculated by taking the negative vectorial sum of $\vec {p_{\text {T}}}$ of tracks satisfying the same quality criteria as the TST tracks. Similar to the TST, tracks with poor momentum resolution or without corresponding calorimeter deposits are removed. Because of Bremsstrahlung within the ID, the electron ${p}_{\text {T}}$ is determined more precisely by the calorimeter than by the ID. Therefore, the Track $E_{\text {T}}^{\text {miss}}$  algorithm uses the electron ${p}_{\text {T}}$ measurement in the calorimeter and removes tracks overlapping its shower. Calorimeter deposits from photons are not added because they cannot be reliably associated to particular *pp* interactions. For muons, the ID track ${p}_{\text {T}}$ is used and not the fits combining the ID and MS ${p}_{\text {T}}$ . For events without any reconstructed jets, the Track and TST $E_{\text {T}}^{\text {miss}}$  would have similar values, but differences could still originate from muon track measurements as well as reconstructed photons or calorimeter deposits from $\tau _{\mathrm{had}{\text {-}}\mathrm{vis}}$, which are only included in the TST.

The soft term for the Track $E_{\text {T}}^{\text {miss}}$  is defined to be identical to the TST by excluding tracks associated with the high-${p}_{\text {T}}$ physics objects used in Eq. (2).

## Comparison of $E_{\text {T}}^{\text {miss}}$  distributions in data and MC simulation

In this section, basic $E_{\text {T}}^{\text {miss}}$  distributions before and after pileup suppression in $\mathrm{Z} \rightarrow \ell{}\ell$  and $W\rightarrow \ell {}\nu$  data events are compared to the distributions from the MC signal plus relevant background samples. All distributions in this section include the dominant systematic uncertainties on the high-$p_{\text {T}}$ objects, the $\vec {E}_\mathrm{T}^{\mathrm {\ miss,soft}}$ (described in Sect. 8) and pileup modelling [7]. The systematics listed above are the largest systematic uncertainties in the $E_{\mathrm {T}}^{\mathrm {miss}}$ for *Z* and *W* samples.

### Modelling of $\mathrm{Z} \rightarrow \ell{}\ell$  events

**Fig. 3:**
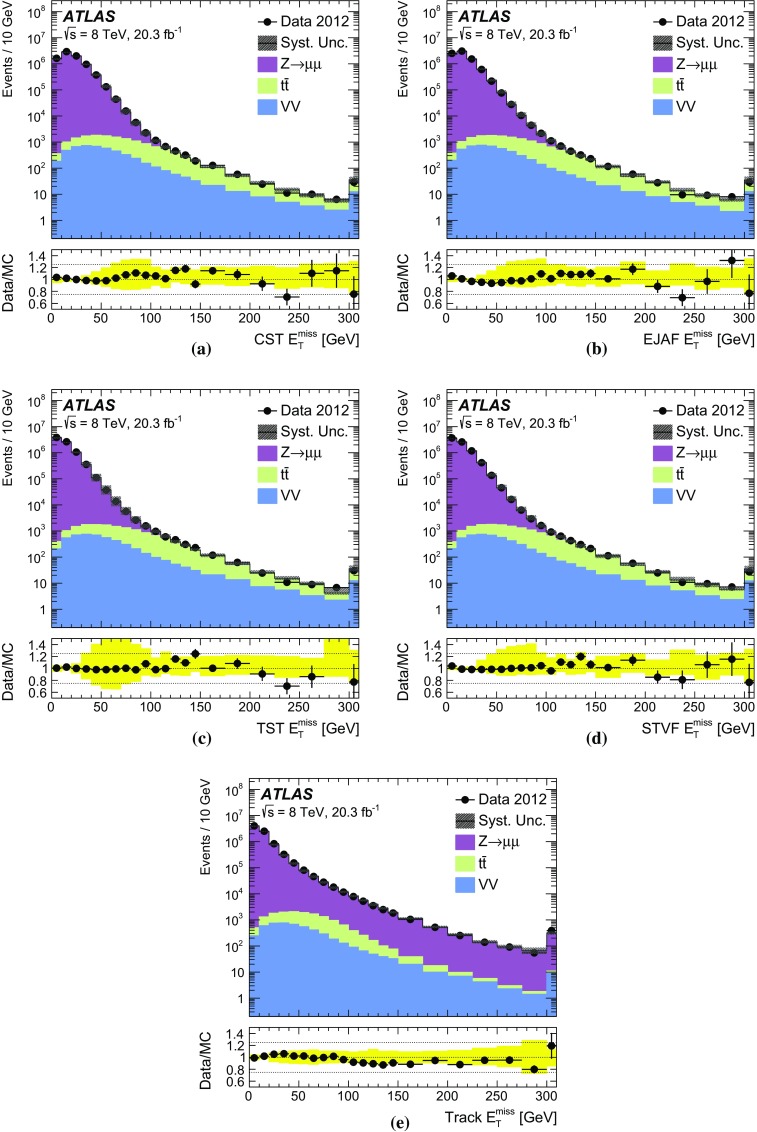
Distributions of the $E_{\mathrm {T}}^{\mathrm {miss}}$ with the **a** CST, **b** EJAF, **c** TST, **d** STVF, and **e** Track $E_{\text {T}}^{\text {miss}}$ are shown in data and MC simulation events satisfying the $\mathrm{Z} \rightarrow \mu {}\mu$ selection. The *lower panel of the figures* shows the ratio of data to MC simulation, and the *bands* correspond to the combined systematic and MC statistical uncertainties. The far right bin includes the integral of all events with $E_{\text {T}}^{\text {miss}}$ above 300 $\text {GeV}$

The CST, EJAF, TST, STVF, and Track $E_{\mathrm {T}}^{\mathrm {miss}}$ distributions for $\mathrm{Z} \rightarrow \mu {}\mu$ data and simulation are shown in Fig. 3. The *Z* boson signal region, which is defined in Sect. 3.2, has better than 99% signal purity. The MC simulation agrees with data for all $E_{\mathrm {T}}^{\mathrm {miss}}$ reconstruction algorithms within the assigned systematic uncertainties. The mean and the standard deviation of the $E_{\text {T}}^{\text {miss}}$  distribution is shown for all of the $E_{\text {T}}^{\text {miss}}$  algorithms in $Z \rightarrow \mu {}\mu$  inclusive simulation in Table 4. The CST $E_{\text {T}}^{\text {miss}}$  has the highest mean $E_{\text {T}}^{\text {miss}}$ and thus the broadest $E_{\mathrm {T}}^{\mathrm {miss}}$ distribution. All of the $E_{\mathrm {T}}^{\mathrm {miss}}$ algorithms with pileup suppression have narrower $E_{\mathrm {T}}^{\mathrm {miss}}$ distributions as shown by their smaller mean $E_{\text {T}}^{\text {miss}}$ values. However, those algorithms also have non-Gaussian tails in the $E_\mathrm{x}^\mathrm{miss}$ and $E_\mathrm{y}^\mathrm{miss}$ distributions, which contribute to the region with $E_{\mathrm {T}}^{\mathrm {miss}}$ $\gtrsim$50 $\text {GeV}$. The Track $E_{\mathrm {T}}^{\mathrm {miss}}$ has the largest tail because it does not include contributions from the neutral particles, and this results in it having the largest standard deviation.

**Table 4:** The mean and standard deviation of the $E_{\text {T}}^{\text {miss}}$  distributions in $\mathrm{Z} \rightarrow \mu {}\mu$  inclusive simulation

$E_{\text {T}}^{\text {miss}}$ alg.	Mean ± SD [GeV]
CST $E_{\text {T}}^{\text {miss}}$	20.4 ± 12.5
EJAF $E_{\text {T}}^{\text {miss}}$	16.8 ± 11.5
TST $E_{\text {T}}^{\text {miss}}$	13.2 ± 10.3
STVF $E_{\text {T}}^{\text {miss}}$	13.8 ± 10.8
Track $E_{\text {T}}^{\text {miss}}$	13.9 ± 14.4

The tails of the $E_{\mathrm {T}}^{\mathrm {miss}}$ distributions in Fig. 3 for $Z \rightarrow \mu {}\mu$  data are observed to be compatible with the sum of expected signal and background contributions, namely $t\bar{t}$  and the summed diboson (*VV*) processes including *WW*, *WZ*, and *ZZ*, which all have high-${p}_{\text {T}}$  neutrinos in their final states. Instrumental effects can show up in the tails of the $E_{\mathrm {T}}^{\mathrm {miss}}$, but such effects are small.

The $E_{\text {T}}^{\text {miss}}$ $\phi$ distribution is not shown in this paper but is very uniform, having less than 4 parts in a thousand difference from positive and negative $\phi$. Thus the $\phi$-asymmetry is greatly reduced from that observed in Ref. [1].

The increase in systematic uncertainties in the range 50–120 $\text {GeV}$ in Fig. 3 comes from the tail of the $E_{\text {T}}^{\text {miss}}$  distribution for the simulated $\mathrm{Z} \rightarrow \mu {}\mu$  events. The increased width in the uncertainty band is asymmetric because many systematic uncertainties increase the $E_{\text {T}}^{\text {miss}}$  tail in $\mathrm{Z} \rightarrow \mu {}\mu$  events by creating an imbalance in the transverse momentum. The largest of these systematic uncertainties are those associated with the jet energy resolution, the jet energy scale, and pileup. The pileup systematic uncertainties affect mostly the CST and EJAF $E_{\text {T}}^{\text {miss}}$, while the jet energy scale uncertainty causes the larger systematic uncertainty for the TST and STVF $E_{\text {T}}^{\text {miss}}$ . The Track $E_{\text {T}}^{\text {miss}}$  does not have the same increase in systematic uncertainties because it does not make use of reconstructed jets. Above 120 $\text {GeV}$, most events have a large intrinsic $E_{\text {T}}^{\text {miss}}$ , and the systematic uncertainties on the $E_{\text {T}}^{\text {miss}}$ , especially the soft term, are smaller.

**Fig. 4:**
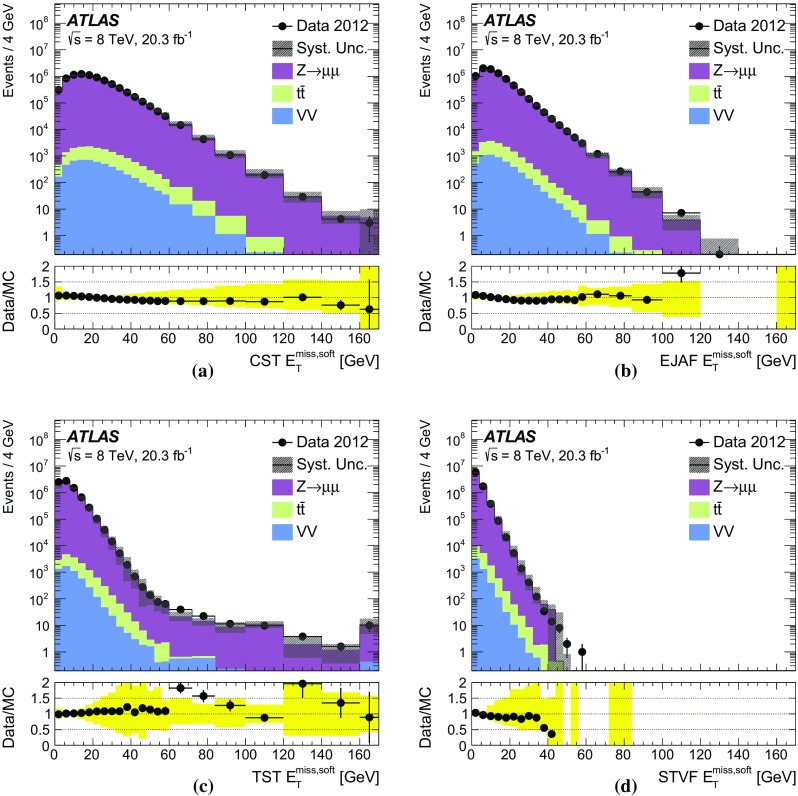
Distributions of the soft term for the **a** CST, **b** EJAF, **c** TST, and **d** STVF are shown in data and MC simulation events satisfying the $\mathrm{Z} \rightarrow \mu {}\mu$ selection. The *lower panel* of the figures show the ratio of data to MC simulation, and the *bands* correspond to the combined systematic and MC statistical uncertainties. The far right bin includes the integral of all events with $E_\mathrm{T}^{\mathrm {miss,soft}}$ above 160 $\text {GeV}$

Figure 4 shows the soft-term distributions. The pileup-suppressed $E_{\text {T}}^{\text {miss}}$  algorithms generally have a smaller mean soft term as well as a sharper peak near zero compared to the CST. Among the $E_{\text {T}}^{\text {miss}}$  algorithms, the soft term from the EJAF algorithm shows the smallest change relative to the CST. The TST has a sharp peak near zero similar to the STVF but with a longer tail, which mostly comes from individual tracks. These tracks are possibly mismeasured and further studies are planned. The simulation under-predicts the TST relative to the observed data between 60–85 $\text {GeV}$, and the differences exceed the assigned systematic uncertainties. This region corresponds to the transition from the narrow core to the tail coming from high-$p_{\text {T}}$ tracks. The differences between data and simulation could be due to mismodelling of the rate of mismeasured tracks, for which no systematic uncertainty is applied. The mismeasured-track cleaning, as discussed in Sect. 4.1.2, reduces the TST tail starting at 120 $\text {GeV}$, and this region is modelled within the assigned uncertainties. The mismeasured-track cleaning for tracks below 120 $\text {GeV}$ and entering the TST is not optimal, and future studies aim to improve this.

**Fig. 5:**
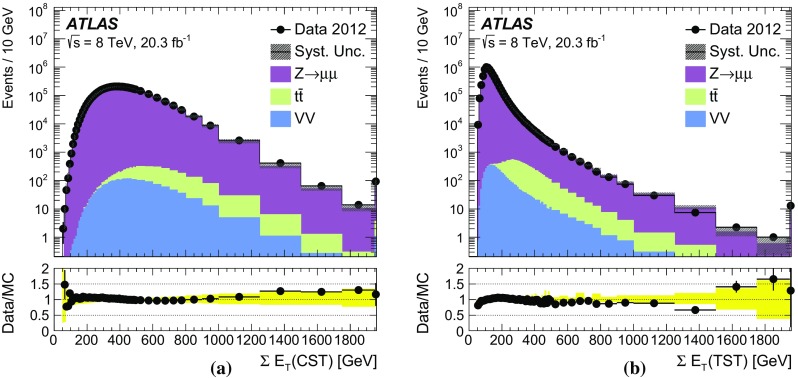
Distributions of **a** $\Sigma E_{\mathrm {T}}$ (CST) and **b** $\Sigma E_{\mathrm {T}}$ (TST) are shown in data and MC simulation events satisfying the $\mathrm{Z} \rightarrow \mu {}\mu$ selection. The *lower panel* of the figures show the ratio of data to MC simulation, and the *bands* correspond to the combined systematic and MC statistical uncertainties. The far right bin includes the integral of all events with $\Sigma E_{\mathrm {T}}$ above 2000 $\text {GeV}$

The $E_{\text {T}}^{\text {miss}}$ resolution is expected to be proportional to $\sqrt{\Sigma E_{\mathrm {T}}}$ when both quantities are measured with the calorimeter alone [1]. While this proportionality does not hold for tracks, it is nevertheless interesting to understand the modelling of $\Sigma E_{\mathrm {T}}$ and the dependence of $E_{\text {T}}^{\text {miss}}$ resolution on it. Figure 5 shows the $\Sigma E_{\mathrm {T}}$ distribution for $\mathrm{Z} \rightarrow \mu {}\mu$ data and MC simulation both for the TST and the CST algorithms. The $\Sigma E_{\mathrm {T}}$  is typically larger for the CST algorithm than for the TST because the former includes energy deposits from pileup as well as neutral particles and forward contributions beyond the ID volume. The reduction of pileup contributions in the soft and jet terms leads to the $\Sigma E_{\mathrm {T}}$ (TST) having a sharper peak at around 100 $\text {GeV}$ followed by a large tail, due to high-${p}_{\text {T}}$  muons and large $\sum p_{\mathrm {T}}^{\mathrm {jets}}$. The data and simulation agree within the uncertainties for the $\Sigma E_{\mathrm {T}}$ (CST) and $\Sigma E_{\mathrm {T}}$ (TST) distributions.

### Modelling of $W\rightarrow \ell {}\nu$  events

In this section, the selection requirements for the $m_{\mathrm {T}}$ and $E_{\text {T}}^{\text {miss}}$  distributions are defined using the same $E_{\text {T}}^{\text {miss}}$  algorithm as that labelling the distribution (e.g. selection criteria are applied to the CST $E_{\text {T}}^{\text {miss}}$  for distributions showing the CST $E_{\text {T}}^{\text {miss}}$ ). The intrinsic $E_{\text {T}}^{\text {miss}}$  in $W\rightarrow \ell {}\nu$  events allows a comparison of the $E_{\mathrm {T}}^{\mathrm {miss}}$ scale between data and simulation. The level of agreement between data and MC simulation for the $E_{\text {T}}^{\text {miss}}$  reconstruction algorithms is studied using $W\rightarrow e{}v$  events with the selection defined in Sect. 3.3.

**Fig. 6:**
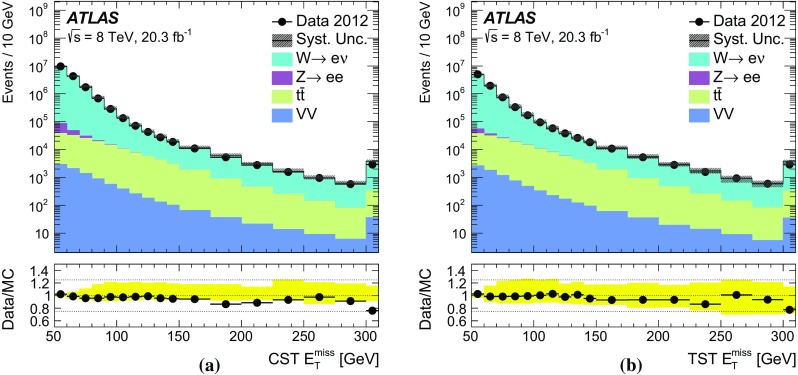
Distributions of the **a** CST and **b** TST $E_{\text {T}}^{\text {miss}}$  as measured in a data sample of $W\rightarrow e{}v$  events. The *lower panel* of the figures show the ratio of data to MC simulation, and the *bands* correspond to the combined systematic and MC statistical uncertainties. The far right bin includes the integral of all events with $E_{\text {T}}^{\text {miss}}$ above 300 $\text {GeV}$

The CST and TST $E_{\text {T}}^{\text {miss}}$  distributions in $W\rightarrow e{}v$  events are shown in Fig. 6. The $W\rightarrow \tau {}v$  contributions are combined with $W\rightarrow e{}v$  events in the figure. The data and MC simulation agree within the assigned systematic uncertainties for both the CST and TST $E_{\text {T}}^{\text {miss}}$  algorithms. The other $E_{\text {T}}^{\text {miss}}$ algorithms show similar levels of agreement between data and MC simulation.

## Performance of the $E_{\text {T}}^{\text {miss}}$  in data and MC simulation

### Resolution of $E_{\text {T}}^{\text {miss}}$

The $E_\mathrm{x}^\mathrm{miss}$ and $E_\mathrm{y}^\mathrm{miss}$ are expected to be approximately Gaussian distributed for $\mathrm{Z} \rightarrow \ell{}\ell$  events as discussed in Ref. [1]. However, because of the non-Gaussian tails in these distributions, especially for the pileup-suppressing $E_{\text {T}}^{\text {miss}}$  algorithms, the root-mean-square (RMS) is used to estimate the resolution. This includes important information about the tails, which would be lost if the result of a Gaussian fit over only the core of the distribution were used instead. The resolution of the $E_{\text {T}}^{\text {miss}}$  distribution is extracted using the RMS from the combined distribution of $E_\mathrm{x}^\mathrm{miss}$ and $E_\mathrm{y}^\mathrm{miss}$, which are determined to be independent from correlation studies. The previous ATLAS $E_{\text {T}}^{\text {miss}}$  performance paper [1] studied the resolution defined by the width of Gaussian fits in a narrow range of $\pm 2$RMS around the mean and used a separate study to investigate the tails. Therefore, the results of this paper are not directly comparable to those of the previous study. The resolutions presented in this paper are expected to be larger than the width of the Gaussian fitted in this manner because the RMS takes into account the tails.

In this section, the resolution for the $E_{\text {T}}^{\text {miss}}$ is presented for $\mathrm{Z} \rightarrow \mu {}\mu$  events using both data and MC simulation. Unless it is a simulation-only figure (labelled with “Simulation” under the ATLAS label), the MC distribution includes the signal sample (e.g. $\mathrm{Z} \rightarrow \mu {}\mu$ ) as well as diboson, $t\bar{t}$ , and *tW* samples.

#### Resolution of the $E_{\text {T}}^{\text {miss}}$  as a function of the number of reconstructed vertices

**Fig. 7:**
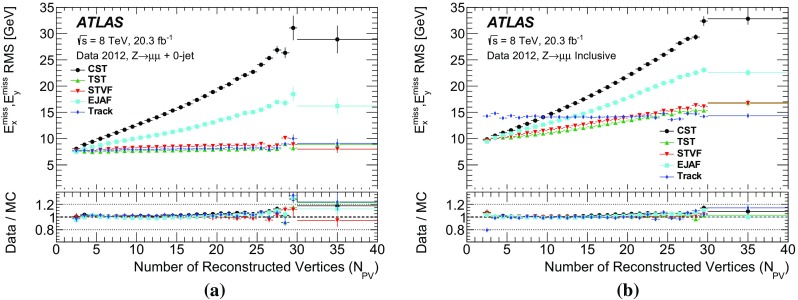
The resolution obtained from the combined distribution of $E_\mathrm{x}^\mathrm{miss}$ and $E_\mathrm{y}^\mathrm{miss}$ for the CST, STVF, EJAF, TST, and Track $E_{\mathrm {T}}^{\mathrm {miss}}$ algorithms as a function of ${N}_{\mathrm {PV}}$ in **a** 0-jet and **b** inclusive $\mathrm{Z} \rightarrow \mu {}\mu$  events in data. The *insets* at the *bottom* of the figures show the ratios of the data to the MC predictions

The stability of the $E_{\text {T}}^{\text {miss}}$  performance as a function of the amount of pileup is estimated by studying the $E_{\mathrm {T}}^{\mathrm {miss}}$ resolution as a function of the number of reconstructed vertices (${N}_{\mathrm {PV}}$) for $\mathrm{Z} \rightarrow \mu {}\mu$  events as shown in Fig. 7. The bin edge is always including the lower edge and not the upper. For example, the events with ${N}_{\mathrm {PV}}$ in the inclusive range 30–39 are combined because of small sample size. In addition, very few events were collected below ${N}_{\mathrm {PV}}$ of 2 during 2012 data taking. Events in which there are no reconstructed jets with $p_{\text {T}}$  > 20 $\text {GeV}$ are referred to collectively as the 0-jet sample. Distributions are shown here for both the 0-jet and inclusive samples. For both samples, the data and MC simulation agree within 2% up to around ${N}_{\mathrm {PV}}$ $=$ 15 but the deviation grows to around 5–10% for ${N}_{\mathrm {PV}}$ > 25, which might be attributed to the decreasing sample size. All of the $E_{\text {T}}^{\text {miss}}$  distributions show a similar level of agreement between data and simulation across the full range of ${N}_{\mathrm {PV}}$.

For the 0-jet sample in Fig. 7a, the STVF, TST, and Track $E_{\text {T}}^{\text {miss}}$  resolutions all have a small slope with respect to ${N}_{\mathrm {PV}}$, which implies stability of the resolution against pileup. In addition, their resolutions agree within 1 $\text {GeV}$ throughout the ${N}_{\mathrm {PV}}$ range. In the 0-jet sample, the TST and Track $E_{\mathrm {T}}^{\mathrm {miss}}$ are both primarily reconstructed from tracks; however, small differences arise mostly from accounting for photons in the TST $E_{\text {T}}^{\text {miss}}$  reconstruction algorithm. The CST $E_{\text {T}}^{\text {miss}}$  is directly affected by the pileup as its reconstruction does not apply any pileup suppression techniques. Therefore, the CST $E_{\text {T}}^{\text {miss}}$  has the largest dependence on ${N}_{\mathrm {PV}}$, with a resolution ranging from 7 $\text {GeV}$ at ${N}_{\mathrm {PV}}$ $=$ 2 to around 23 $\text {GeV}$ at ${N}_{\mathrm {PV}}$ $=$ 25. The $E_{\text {T}}^{\text {miss}}$  resolution of the EJAF distribution, while better than that of the CST $E_{\text {T}}^{\text {miss}}$ , is not as good as that of the other pileup-suppressing algorithms.

For the inclusive sample in Fig. 7b, the Track $E_{\mathrm {T}}^{\mathrm {miss}}$ is the most stable with respect to pileup with almost no dependence on ${N}_{\mathrm {PV}}$. For ${N}_{\mathrm {PV}}$ > 20, the Track $E_{\text {T}}^{\text {miss}}$  has the best resolution showing that pileup creates a larger degradation in the resolution of the other $E_{\text {T}}^{\text {miss}}$  distributions than excluding neutral particles, as the Track $E_{\text {T}}^{\text {miss}}$  algorithm does. The EJAF $E_{\text {T}}^{\text {miss}}$  algorithm does not reduce the pileup dependence as much as the TST and STVF $E_{\text {T}}^{\text {miss}}$  algorithms, and the CST $E_{\text {T}}^{\text {miss}}$  again has the largest dependence on ${N}_{\mathrm {PV}}$.

**Fig. 8:**
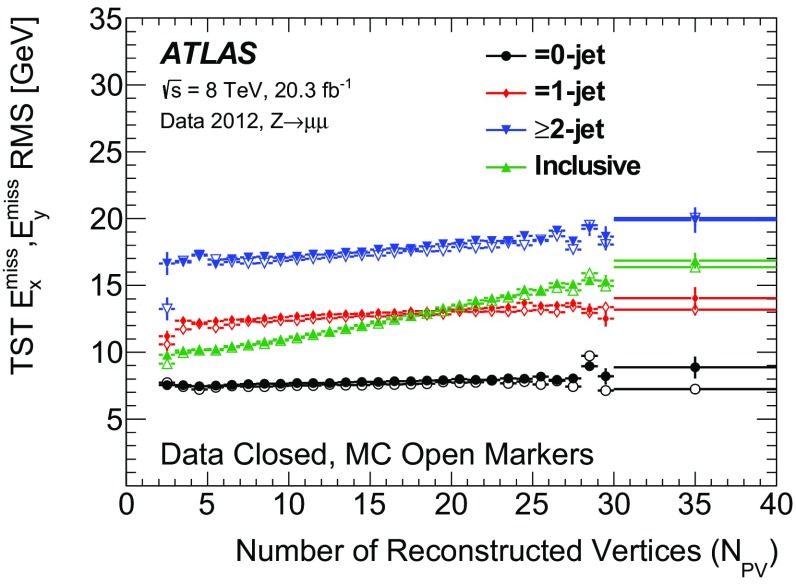
The resolution of the combined distribution of $E_\mathrm{x}^\mathrm{miss}$ and $E_\mathrm{y}^\mathrm{miss}$ for the TST $E_{\mathrm {T}}^{\mathrm {miss}}$ as a function of ${N}_{\mathrm {PV}}$ for the 0-jet, 1-jet, $\ge$ 2-jet, and inclusive $\mathrm{Z} \rightarrow \mu {}\mu$  samples. The data (*closed markers*) and MC simulation (*open markers*) are overlaid. The jet counting uses the same $\text {JVF}$ criterion as the TST $E_{\text {T}}^{\text {miss}}$  reconstruction algorithm

Figure 7 also shows that the pileup dependence of the TST, CST, EJAF and STVF $E_{\text {T}}^{\text {miss}}$  is smaller in the 0-jet sample than in the inclusive sample. Hence, the evolution of the $E_{\text {T}}^{\text {miss}}$  resolution is shown for different numbers of jets in Fig. 8 with the TST $E_{\text {T}}^{\text {miss}}$  algorithm as a representative example. The jet counting for this figure includes only the jets used by the TST $E_{\text {T}}^{\text {miss}}$  algorithm, so the $\text {JVF}$ criterion discussed in Sect. 4.1.3 is applied. Comparing the 0-jet, 1-jet and $\ge$2-jet distributions, the resolution is degraded by 4–5 $\text {GeV}$ with each additional jet, which is much larger than any dependence on ${N}_{\mathrm {PV}}$. The inclusive distribution has a larger slope with respect to ${N}_{\mathrm {PV}}$ than the individual jet categories, which indicates that the behaviour seen in the inclusive sample is driven by an increased number of pileup jets included in the $E_{\text {T}}^{\text {miss}}$  calculation at larger ${N}_{\mathrm {PV}}$.

#### Resolution of the $E_{\text {T}}^{\text {miss}}$  as a function of $\Sigma E_{\mathrm {T}}$

**Fig. 9:**
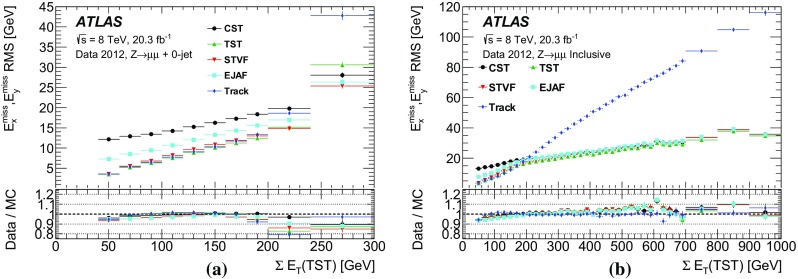
The resolution of the combined distribution of $E_\mathrm{x}^\mathrm{miss}$ and $E_\mathrm{y}^\mathrm{miss}$ for the CST, STVF, EJAF, TST, and Track $E_{\mathrm {T}}^{\mathrm {miss}}$ as a function of $\Sigma E_{\mathrm {T}}$ (TST) in $\mathrm{Z} \rightarrow \mu {}\mu$  events in data for the **a** 0-jet and **b** inclusive samples. The insets at the bottom of the figures show the ratios of the data to the MC predictions

The resolutions of $E_{\text {T}}^{\text {miss}}$ , resulting from the different reconstruction algorithms, are compared as a function of the scalar sum of transverse momentum in the event, as calculated using Eq. (4). The CST $E_{\text {T}}^{\text {miss}}$  resolution is observed to depend linearly on the square root of the $\Sigma E_{\mathrm {T}}$  computed with the CST $E_{\text {T}}^{\text {miss}}$  components in Ref. [1]. However, the $\Sigma E_{\mathrm {T}}$  used in this subsection is calculated with the TST $E_{\text {T}}^{\text {miss}}$  algorithm. This allows studies of the resolution as a function of the momenta of particles from the selected PV without including the amount of pileup activity in the event. Figure 9 shows the resolution as a function of $\Sigma E_{\mathrm {T}}$ (TST) for $Z \rightarrow \mu \mu$  data and MC simulation in the 0-jet and inclusive samples.

In the 0-jet sample shown in Fig. 9a, the use of tracking information in the soft term, especially for the STVF, TST, and Track $E_{\mathrm {T}}^{\mathrm {miss}}$, greatly improves the resolution relative to the CST $E_{\text {T}}^{\text {miss}}$ . The EJAF $E_{\text {T}}^{\text {miss}}$ has a better resolution than that of the CST $E_{\text {T}}^{\text {miss}}$ but does not perform as well as the other reconstruction algorithms. All of the resolution curves have an approximately linear increase with $\Sigma E_{\mathrm {T}}$ (TST); however, the Track $E_{\text {T}}^{\text {miss}}$ resolution increases sharply starting at $\Sigma E_{\mathrm {T}}$ (TST) $=$ 200 $\text {GeV}$ due to missed neutral contributions like photons. The resolution predicted by the simulation is about 5% larger than in data for all $E_{\text {T}}^{\text {miss}}$  algorithms at $\Sigma E_{\mathrm {T}}$ (TST) $=$ 50 $\text {GeV}$, but agreement improves as $\Sigma E_{\mathrm {T}}$ (TST) increases until around $\Sigma E_{\mathrm {T}}$ (TST) $=$ 200 $\text {GeV}$. Events with jets can end up in the 0-jet event selection, for example, if a jet is misidentified as a hadronically decaying $\tau$-lepton. The $\sum p_{\mathrm {T}}^{\tau }$ increases with $\Sigma E_{\mathrm {T}}$ (TST), and the rate of jets misreconstructed as hadronically decaying $\tau$-leptons is not well modelled by the simulation, which leads to larger $E_{\text {T}}^{\text {miss}}$ resolution at high $\Sigma E_{\mathrm {T}}$ (TST) than that observed in the data. The Track $E_{\text {T}}^{\text {miss}}$ can be more strongly affected by misidentified jets because neutral particles from the high-${p}_{\text {T}}$ jets are not included.

For the inclusive sample in Fig. 9b, the pileup-suppressed $E_{\mathrm {T}}^{\mathrm {miss}}$ distributions have better resolution than the CST $E_{\text {T}}^{\text {miss}}$  for $\Sigma E_{\mathrm {T}}$ (TST) < 200 $\text {GeV}$, but these events are mostly those with no associated jets. For higher $\Sigma E_{\mathrm {T}}$ (TST), the impact from the $\Sigma E_{\mathrm {T}}^\mathrm{jets}$  term starts to dominate the resolution as well as the $\Sigma E_{\mathrm {T}}$ (TST). Since the vector sum of jet momenta is mostly common^7^ to all $E_{\mathrm {T}}^{\mathrm {miss}}$ algorithms except for the Track $E_{\mathrm {T}}^{\mathrm {miss}}$, those algorithms show similar performance in terms of the resolution. At larger $\Sigma E_{\mathrm {T}}$ (TST), the Track $E_{\mathrm {T}}^{\mathrm {miss}}$ resolution begins to degrade relative to the other algorithms because it does not include the high-${p}_{\text {T}}$ neutral particles coming from jets. The ratio of data to MC simulation for the Track $E_{\text {T}}^{\text {miss}}$  distribution is close to one, while for other algorithms the MC simulation is below the data by about 5% at large $\Sigma E_{\mathrm {T}}$ (TST). While the Track $E_{\text {T}}^{\text {miss}}$  appears well modelled for the Alpgen $+$ Pythia simulation used in this figure, the modelling depends strongly on the parton shower model.

### The $E_{\text {T}}^{\text {miss}}$ response

The balance of $\vec {E}_{{\mathrm{T}}}^{\mathrm{miss}}$ against the vector boson $\vec {p_{\text {T}}}$  in $W/Z+$jets events is used to evaluate the $E_{\text {T}}^{\text {miss}}$  response. A lack of balance is a global indicator of biases in $E_{\text {T}}^{\text {miss}}$  reconstruction and implies a systematic misestimation of at least one of the $E_{\text {T}}^{\text {miss}}$ terms, possibly coming from an imperfect selection or calibration of the reconstructed physics objects. The procedure to evaluate the response differs between $Z\mathrm {+jets}$ events (Sect. 6.2.1) and $W\mathrm {+jets}$ events (Sect. 6.2.2) because of the high-$p_{\text {T}}$  neutrino in the leptonic decay of the *W* boson.

#### Measuring $E_{\text {T}}^{\text {miss}}$  recoil versus $p_{\mathrm {T}}^{Z}$

In events with $\mathrm{Z} \rightarrow \mu {}\mu$  decays, the $\vec {p_{\text {T}}}$  of the *Z* boson defines an axis in the transverse plane of the ATLAS detector, and for events with 0-jets, the $\vec {E}_{{\mathrm{T}}}^{\mathrm{miss}}$ should balance the $\vec {p_{\text {T}}}$  of the *Z* boson ($\vec {p}_{\mathrm {T}}^{Z}\;$) along this axis. Comparing the response in events with and without jets allows distinction between the jet and soft-term responses. The component of the $\vec {E}_{{\mathrm{T}}}^{\mathrm{miss}}$ along the $\vec {p}_{\mathrm {T}}^{Z}\;$ axis is sensitive to biases in detector responses [51]. The unit vector of $\vec {p}_{\mathrm {T}}^{Z}\;$ is labelled as $\hat{\mathcal {A}}_Z$ and is defined as:

12 $$\begin{aligned} \hat{\mathcal {A}}_Z=\frac{\vec {p_{\mathrm {T}}}^{\ell ^+}+\vec {p_{\mathrm {T}}}^{\ell ^-}}{|\vec {p_{\mathrm {T}}}^{\ell ^+}+\vec {p_{\mathrm {T}}}^{\ell ^-}|}, \end{aligned}$$

where $\vec {p_{\mathrm {T}}}^{\ell ^+}$ and $\vec {p_{\mathrm {T}}}^{\ell ^-}$ are the transverse momentum vectors of the leptons from the *Z* boson decay.

The recoil of the *Z* boson is measured by removing the *Z* boson decay products from the $\vec {E}_{{\mathrm{T}}}^{\mathrm{miss}}$ and is computed as

13 $$\begin{aligned} \vec {\mathcal {R}}{}= \vec {E}_{{\mathrm{T}}}^{\mathrm{miss}}{}+\vec {p}_{\mathrm {T}}^{Z}\;{}. \end{aligned}$$

**Fig. 10:**
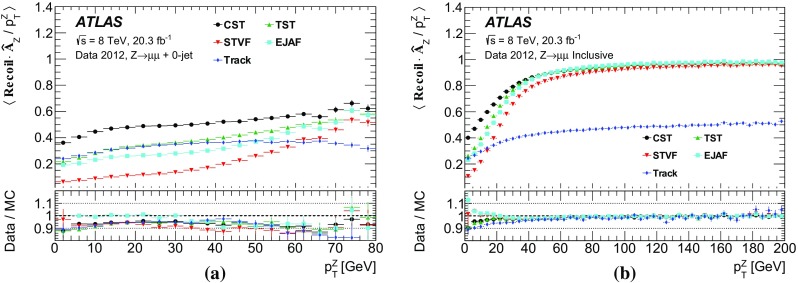
$\langle \vec {\mathcal {R}}{}\cdot \hat{\mathcal {A}}_Z{}/p_{\mathrm {T}}^{Z}{}\rangle$ as a function $p_{\mathrm {T}}^{Z}$ for the **a** 0-jet and **b** inclusive events in $\mathrm{Z} \rightarrow \mu {}\mu$  data. The *insets* at the *bottom* of the figures show the ratios of the data to the MC predictions

Since the $\vec {E}_{{\mathrm{T}}}^{\mathrm{miss}}$ includes a negative vector sum over the lepton momenta, the addition of $\vec {p}_{\mathrm {T}}^{Z}\;$ removes its contribution. With an ideal detector and $E_{\text {T}}^{\text {miss}}$  reconstruction algorithm, $\mathrm{Z} \rightarrow \ell{}\ell$  events have no $E_{\text {T}}^{\text {miss}}$ , and the $\vec {\mathcal {R}}$ balances with $\vec {p}_{\mathrm {T}}^{Z}\;$ exactly. For the real detector and $E_{\text {T}}^{\text {miss}}$  reconstruction algorithm, the degree of balance is measured by projecting the recoil onto $\hat{\mathcal {A}}_Z$, and the relative recoil is defined as the projection $\vec {\mathcal {R}}{}\cdot \hat{\mathcal {A}}_Z{}$ divided by $p_{\mathrm {T}}^{Z}$, which gives a dimensionless estimate that is unity if the $E_{\text {T}}^{\text {miss}}$  is ideally reconstructed and calibrated. Figure 10 shows the mean relative recoil versus $p_{\mathrm {T}}^{Z}$ for $\mathrm{Z} \rightarrow \mu {}\mu$ events where the average value is indicated by angle brackets. The data and MC simulation agree within around 10% for all $E_{\text {T}}^{\text {miss}}$ algorithms for all $p_{\mathrm {T}}^{Z}$; however, the agreement is a few percent worse for $p_{\mathrm {T}}^{Z}$ > 50 $\text {GeV}$ in the 0-jet sample.

The $\mathrm{Z} \rightarrow \mu {}\mu$  events in the 0-jet sample in Fig. 10a have a relative recoil significantly lower than unity ($\langle \vec {\mathcal {R}}{}\cdot \hat{\mathcal {A}}_Z{}/p_{\mathrm {T}}^{Z}{}\rangle$ < 1) throughout the $p_{\mathrm {T}}^{Z}$ range. In the 0-jet sample, the relative recoil estimates how well the soft term balances the $\vec {p_{\text {T}}}$ of muons from the *Z* decay, which are better measured than the soft term. The relative recoil below one indicates that the soft term is underestimated. The CST $E_{\text {T}}^{\text {miss}}$ has a relative recoil measurement of $\langle \vec {\mathcal {R}}{}\cdot \hat{\mathcal {A}}_Z{}/p_{\mathrm {T}}^{Z}{}\rangle$ $\sim$ 0.5 throughout the $p_{\mathrm {T}}^{Z}$ range, giving it the best recoil performance among the $E_{\text {T}}^{\text {miss}}$  algorithms. The TST and Track $E_{\text {T}}^{\text {miss}}$  have slightly larger biases than the CST $E_{\text {T}}^{\text {miss}}$ because neutral particles are not considered in the soft term. The TST $E_{\text {T}}^{\text {miss}}$  recoil improves relative to that of the Track $E_{\text {T}}^{\text {miss}}$  for $p_{\mathrm {T}}^{Z}$ > 40 $\text {GeV}$ because of the inclusion of photons in its reconstruction. The relative recoil distribution for the STVF $E_{\text {T}}^{\text {miss}}$  shows the largest bias for $p_{\mathrm {T}}^{Z}$ < 60 $\text {GeV}$. The STVF algorithm scales the recoil down globally by the factor $\alpha _{\text {STVF}}$  as defined in Eq. (11), and this correction decreases the already underestimated soft term. The $\alpha _{\text {STVF}}$  does increase with $p_{\mathrm {T}}^{Z}$ going from 0.06 at $p_{\mathrm {T}}^{Z}$ $=$ 0 $\text {GeV}$ to around 0.15 at $p_{\mathrm {T}}^{Z}$ $=$ 50 $\text {GeV}$, and this results in a rise in the recoil, which approaches the TST $E_{\text {T}}^{\text {miss}}$  near $p_{\mathrm {T}}^{Z}$ $\sim$ 70 $\text {GeV}$.

In Fig. 10b, the inclusive $\mathrm{Z} \rightarrow \mu {}\mu$  events have a significantly underestimated relative recoil for $p_{\mathrm {T}}^{Z}$ < 40 $\text {GeV}$. The balance between the $\vec {\mathcal {R}}$ and $\vec {p}_{\mathrm {T}}^{Z}\;$ improves with $p_{\mathrm {T}}^{Z}$ because of an increase in events having high-${p}_{\text {T}}$  calibrated jets recoiling against the *Z* boson. The presence of jets included in the hard term also reduces the sensitivity to the soft term, which is difficult to measure accurately. The difficulty in isolating effects from soft-term contributions from high-${p}_{\text {T}}$  physics objects is one reason why the soft term is not corrected. As with the 0-jet sample, the CST $E_{\text {T}}^{\text {miss}}$ has a significantly under-calibrated relative recoil in the low-$p_{\mathrm {T}}^{Z}$ region, and all of the other $E_{\text {T}}^{\text {miss}}$  algorithms have a lower relative recoil than the CST $E_{\text {T}}^{\text {miss}}$ . Of the pileup-suppressing $E_{\text {T}}^{\text {miss}}$  algorithms, the TST $E_{\text {T}}^{\text {miss}}$  is closest to the relative recoil of the CST $E_{\text {T}}^{\text {miss}}$ . The relative recoil of the Track $E_{\text {T}}^{\text {miss}}$  is significantly lower than unity because the neutral particles recoiling from the *Z* boson are not included in its reconstruction. Finally, the STVF $E_{\text {T}}^{\text {miss}}$ shows the lowest relative recoil among the object-based $E_{\text {T}}^{\text {miss}}$  algorithms as discussed above for Fig. 10a, even lower than the Track $E_{\text {T}}^{\text {miss}}$  for $p_{\mathrm {T}}^{Z}$ < 16 $\text {GeV}$.

#### Measuring $E_{\mathrm {T}}^{\mathrm {miss}}$ response in simulated $W\rightarrow \ell {}\nu$  events

For simulated events with intrinsic $E_{\text {T}}^{\text {miss}}$ , the response is studied by looking at the relative mismeasurement of the reconstructed $E_{\text {T}}^{\text {miss}}$ . This is referred to here as the “linearity”, and is a measure of how consistent the reconstructed $E_{\text {T}}^{\text {miss}}$ is with the $E_{\mathrm {T}}^{\mathrm {miss,True}}$. The linearity is defined as the mean value of the ratio, $(E_{\mathrm {T}}^\mathrm{miss}-E_{\mathrm {T}}^\mathrm{miss,True})/E_{\mathrm {T}}^\mathrm{miss,True}$ and is expected to be zero if the $E_{\mathrm {T}}^{\mathrm {miss}}$ is reconstructed at the correct scale.

**Fig. 11:**
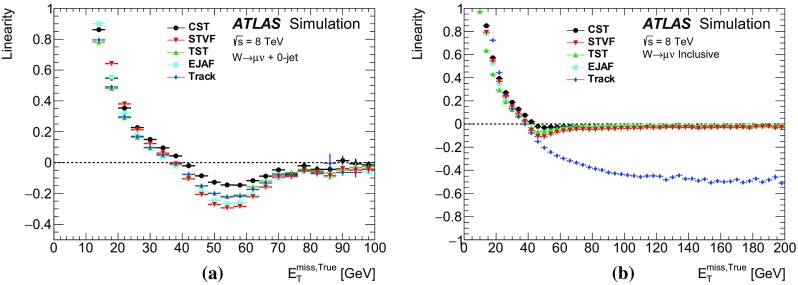
$E_{\mathrm {T}}^{\mathrm {miss}}$ linearity in $W\rightarrow \mu {}v$  MC simulation is shown versus $E_{\mathrm {T}}^{\mathrm {miss,True}}$ in the **a** 0-jet and **b** inclusive events

For the linearity studies, no selection on the $E_{\mathrm {T}}^{\mathrm {miss}}$ or $m_{\mathrm {T}}$ is applied, in order to avoid biases as these are purely simulation-based studies. In Fig. 11, the linearity for $W\rightarrow \mu {}v$  simulated events is presented as a function of the $E_{\mathrm {T}}^{\mathrm {miss,True}}$. Despite the relaxed selection, a positive linearity is evident for $E_{\mathrm {T}}^{\mathrm {miss,True}}$< 40 $\text {GeV}$, due to the finite resolution of the $E_{\mathrm {T}}^{\mathrm {miss}}$ reconstruction and the fact that the reconstructed $E_{\mathrm {T}}^{\mathrm {miss}}$ is positive by definition. The CST $E_{\text {T}}^{\text {miss}}$ has the largest deviation from zero at low $E_{\mathrm {T}}^{\mathrm {miss,True}}$ because it has the largest $E_{\mathrm {T}}^{\mathrm {miss}}$ resolution.

For the events in the 0-jet sample in Fig. 11a, all $E_{\text {T}}^{\text {miss}}$  algorithms have a negative linearity for $E_{\mathrm {T}}^{\mathrm {miss,True}}$ > 40 $\text {GeV}$, which diminishes for $E_{\mathrm {T}}^{\mathrm {miss,True}}$ $\gtrsim 60$ $\text {GeV}$. The region of $E_{\mathrm {T}}^{\mathrm {miss,True}}$ between 40 and 60 $\text {GeV}$ mostly includes events lying in the Jacobian peak of the *W* transverse mass, and these events include mostly on-shell *W* bosons. For $E_{\text {T}}^{\text {miss}}$  $\gtrsim$ 40 $\text {GeV}$, the on-shell *W* boson must have non-zero ${p}_{\text {T}}$ , which typically comes from its recoil against jets. However, no reconstructed or generator-level jets are found in this 0-jet sample. Therefore, most of the events with 40 < $E_{\mathrm {T}}^{\mathrm {miss,True}}$ < 60 $\text {GeV}$ have jets below the 20 $\text {GeV}$ threshold contributing to the soft term, and the soft term is not calibrated. The under-estimation of the soft term, described in Sect. 6.2.1, causes the linearity to deviate further from zero in this region. Events with $E_\mathrm{T}^\mathrm{miss,True}$ >60 $\text {GeV}$ are mostly off-shell *W* bosons that are produced with very low ${p}_{\text {T}}$ . For these events, the $\vec {p_{\text {T}}}$  contributions to the $E_{\text {T}}^{\text {miss}}$  reconstruction come mostly from the well-measured muon $\vec {p_{\text {T}}}$ , and the soft term plays a much smaller role. Hence, the linearity improves as the impact of the soft term decreases with larger $E_\mathrm{T}^\mathrm{miss,True}$.

For inclusive events in Fig. 11b with $E_{\mathrm {T}}^{\mathrm {miss,True}}$ $>40$ $\text {GeV}$, the deviation of the linearity from zero is smaller than 5% for the CST $E_{\text {T}}^{\text {miss}}$. The linearity of the TST $E_{\text {T}}^{\text {miss}}$  is within 10% of unity in the range of 40–60 $\text {GeV}$ and improves for higher $E_{\mathrm {T}}^{\mathrm {miss,True}}$ values. The STVF $E_{\text {T}}^{\text {miss}}$  has the most negative bias in the linearity among the object-based $E_{\text {T}}^{\text {miss}}$  algorithms for $E_{\mathrm {T}}^{\mathrm {miss,True}}$ > 40 $\text {GeV}$. The TST, CST, STVF, and EJAF $E_{\text {T}}^{\text {miss}}$  algorithms perform similarly for all $E_{\mathrm {T}}^{\mathrm {miss,True}}$ values. As expected, the linearity of the Track $E_{\text {T}}^{\text {miss}}$  settles below zero due to not accounting for neutral particles in jets.

### The $\vec {E}_{{\mathrm{T}}}^{\mathrm{miss}}$ angular resolution

The angular resolution is important for the reconstruction of kinematic observables such as the transverse mass of the *W* boson and the invariant mass in $H \rightarrow \tau \tau$  events [52]. For simulated $W\rightarrow \ell {}\nu$ events, the direction of the reconstructed $\vec {E}_{{\mathrm{T}}}^{\mathrm{miss}}$ is compared to the $\vec {E}_{\mathrm{T}}^{\mathrm{miss,True}}$ for each $E_{\text {T}}^{\text {miss}}$  reconstruction algorithm using the difference in the azimuthal angles, $\Delta \phi (\vec {E}_{{\mathrm{T}}}^{\mathrm{miss}},\vec {E}_{\mathrm{T}}^{\mathrm{miss,True}})$ , which has a mean value of zero. The RMS of the distribution is taken as the resolution, which is labelled $\text {RMS}\left( \Delta \phi \right)$ .

**Fig. 12:**
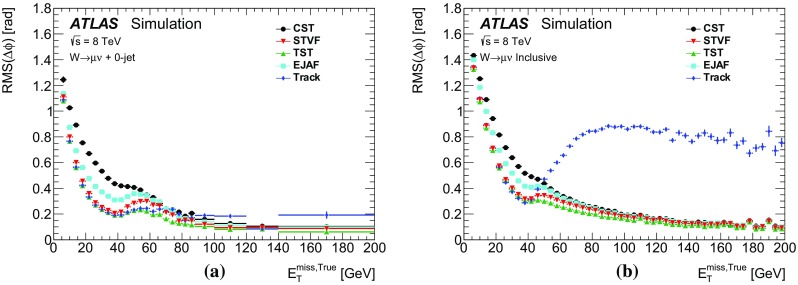
The resolution of $\Delta \phi (\vec {E}_{{\mathrm{T}}}^{\mathrm{miss}},\vec {E}_{\mathrm{T}}^{\mathrm{miss,True}})$ , labelled as $\text {RMS}\left( \Delta \phi \right)$ , is shown for $W\rightarrow \mu {}v$  MC simulation for the **a** 0-jet and **b** inclusive samples

No selection on the $E_{\mathrm {T}}^{\mathrm {miss}}$ or $m_{\mathrm {T}}$ is applied in order to avoid biases. The $\text {RMS}\left( \Delta \phi \right)$  is shown as a function of $E_{\mathrm {T}}^{\mathrm {miss,True}}$ in Fig. 12a for the 0-jet sample in $W\rightarrow \mu {}v$  simulation; the angular resolution generally improves as the $E_{\mathrm {T}}^{\mathrm {miss,True}}$ increases, for all algorithms. For $E_{\mathrm {T}}^{\mathrm {miss,True}}$ $\lesssim$ 120 $\text {GeV}$, the pileup-suppressing algorithms improve the resolution over the CST $E_{\text {T}}^{\text {miss}}$  algorithm, but all of the algorithms produce distributions with similar resolutions in the higher $E_{\mathrm {T}}^{\mathrm {miss,True}}$ region. The increase in $\text {RMS}\left( \Delta \phi \right)$  at around 40–60 $\text {GeV}$ in the 0-jet sample is due to the larger contribution of jets below 20 $\text {GeV}$ entering the soft term as mentioned in Sect. 6.2.2. The distribution from the inclusive sample shown in Fig. 12b has the same pattern as the one from the 0-jet sample, except that the performance of the Track $E_{\text {T}}^{\text {miss}}$  algorithm is again significantly worse. In addition, the transition region near 40 < $E_\mathrm{T}^\mathrm{miss,True}$ < 60 $\text {GeV}$ is smoother as the under-estimation of the soft term becomes less significant due to the presence of events with high-${p}_{\text {T}}$  calibrated jets. The TST $E_{\text {T}}^{\text {miss}}$  algorithm has the best angular resolution for both the 0-jet and inclusive topologies throughout the entire range of $E_{\mathrm {T}}^{\mathrm {miss,True}}$.

### Transverse mass in $W\rightarrow \ell {}\nu$  events

**Fig. 13:**
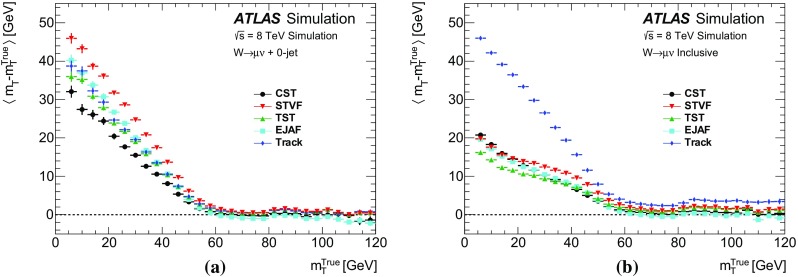
The $\langle m_{\mathrm {T}}- m_{\mathrm {T}}^{\mathrm {True}}\rangle$ is shown versus $m_{\mathrm {T}}^{\mathrm {True}}$ for $W\rightarrow \mu {}v$  MC simulation in the **a** 0-jet and **b** inclusive samples

The *W* boson events are selected using kinematic observables that are computed from the $\vec {E}_{{\mathrm{T}}}^{\mathrm{miss}}$ and lepton transverse momentum. This section evaluates the scale of the $m_{\mathrm {T}}$, as defined in Eq. (1), reconstructed with each $E_{\text {T}}^{\text {miss}}$  definition. The $m_{\mathrm {T}}$ computed using the reconstructed $\vec {E}_{{\mathrm{T}}}^{\mathrm{miss}}$ is compared to the $m_{\mathrm {T}}^{\mathrm {True}}$, which is calculated using the $\vec {E}_{\mathrm{T}}^{\mathrm{miss,True}}$ in $W\rightarrow \mu {}v$  MC simulation. The mean of the difference between the reconstructed and generator-level $m_{\mathrm {T}}$, ($\langle m_{\mathrm {T}}- m_{\mathrm {T}}^{\mathrm {True}}\rangle$), is shown as a function of $m_{\mathrm {T}}^{\mathrm {True}}$ in Figure 13 for the 0-jet and inclusive samples. No $E_{\mathrm {T}}^{\mathrm {miss}}$ or $m_{\mathrm {T}}$ selection is made in these figures, to avoid biases. All distributions for the $E_{\text {T}}^{\text {miss}}$  algorithms have a positive bias at low values of $m_{\mathrm {T}}^{\mathrm {True}}$ coming from the positive-definite nature of the $m_{\mathrm {T}}$ and the finite $E_{\text {T}}^{\text {miss}}$  resolution. For the 0-jet sample, the CST algorithm has the smallest bias for $m_{\mathrm {T}}$ $\lesssim$ 60 $\text {GeV}$ because it includes the neutral particles with no corrections for pileup. However, for the inclusive sample the TST $E_{\text {T}}^{\text {miss}}$ has the smallest bias as the $E_{\text {T}}^{\text {miss}}$  resolution plays a larger role. The STVF and Track $E_{\text {T}}^{\text {miss}}$  have the largest bias for $m_{\mathrm {T}}^{\mathrm {True}}$ < 50 $\text {GeV}$ in the 0-jet and inclusive samples, respectively. This is due to the over-correction in the soft term by $\alpha _{\text {STVF}}$ for the former and from the missing neutral particles in the latter case. For events with $m_{\mathrm {T}}$ $\gtrsim$ 60 $\text {GeV}$, all of the $E_{\text {T}}^{\text {miss}}$  algorithms have $\langle m_{\mathrm {T}}- m_{\mathrm {T}}^{\mathrm {True}}\rangle$ close to zero, with a spread of less than 3 $\text {GeV}$.

### Proxy for $E_{\text {T}}^{\text {miss}}$ significance

The $E_{\text {T}}^{\text {miss}}$  significance is a metric defined to quantify how likely it is that a given event contains intrinsic $E_{\text {T}}^{\text {miss}}$ and is computed by dividing the measured $E_{\mathrm {T}}^{\mathrm {miss}}$ by an estimate of its uncertainty. Using 7 $\text {TeV}$ data, it was shown that the CST $E_{\mathrm {T}}^{\mathrm {miss}}$ resolution follows an approximately stochastic behaviour as a function of $\Sigma E_{\mathrm {T}}$ , computed with the CST components, and is described by

14 $$\begin{aligned} \sigma (E_{\text {T}}^{\text {miss}}) = a \cdot \sqrt{\Sigma E_{\mathrm {T}}}, \end{aligned}$$

where $\sigma (E_{\text {T}}^{\text {miss}})$ is the CST $E_{\text {T}}^{\text {miss}}$  resolution [1]. The typical value of *a* in the 8 $\text {TeV}$ dataset is around 0.97 $\text {GeV}{}^{1/2}$ for the CST $E_{\text {T}}^{\text {miss}}$ . The proxy of the $E_{\text {T}}^{\text {miss}}$  significance presented in this section is defined as the $\frac{1}{a}\cdot$ $E_{\text {T}}^{\text {miss}}$ /$\sqrt{\Sigma E_{\mathrm {T}}}$. This choice is motivated by the linear relationship for the CST $E_{\text {T}}^{\text {miss}}$ between its $\sqrt{\Sigma E_{\mathrm {T}}}$ and its $E_{\text {T}}^{\text {miss}}$  resolution. The same procedure does not work for the TST $E_{\text {T}}^{\text {miss}}$ resolution, so a value of 2.27 $\text {GeV}{}^{1/2}$ is used to tune the *x*-axis so that integral of $\mathrm{Z} \rightarrow \mu {}\mu$ simulation fits the multiples of the standard deviation of a normal distribution at the value of 2. Ideally, only events with large intrinsic $E_{\text {T}}^{\text {miss}}$  have large values of $\frac{1}{a}\cdot$ $E_{\text {T}}^{\text {miss}}$ /$\sqrt{\Sigma E_{\mathrm {T}}}$, while events with no intrinsic $E_{\text {T}}^{\text {miss}}$  such as $\mathrm{Z} \rightarrow \mu {}\mu$  have low values. It is important to point out that in general $\mathrm{Z} \rightarrow \mu {}\mu$ is not a process with large $E_{\text {T}}^{\text {miss}}$ uncertainties or large $\sqrt{\Sigma E_{\mathrm {T}}}$. However, when there are many additional jets (large $\Sigma E_{\mathrm {T}}$ ), there is a significant probability that one of them is mismeasured, which generates fake $E_{\text {T}}^{\text {miss}}$ .

**Fig. 14:**
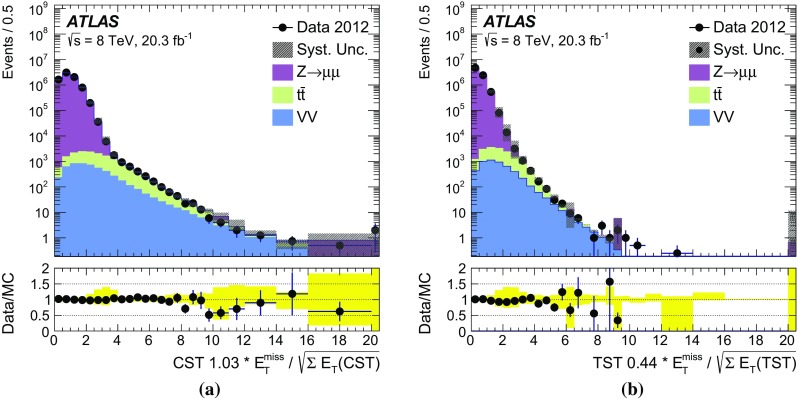
The proxy for $E_{\text {T}}^{\text {miss}}$  significance is shown in data and MC simulation events satisfying the $\mathrm{Z} \rightarrow \mu {}\mu$ selection for the **a** CST and **b** TST $E_{\text {T}}^{\text {miss}}$  algorithms. The *solid band* shows the combined MC statistical and systematic uncertainties, and the *insets* at the *bottom* of the figures show the ratios of the data to the MC predictions. The far right bin includes the integral of all events above 20

The distribution of $\frac{1}{a}\cdot$ $E_{\text {T}}^{\text {miss}}$ /$\sqrt{\Sigma E_{\mathrm {T}}}$ is shown for the CST and TST $E_{\text {T}}^{\text {miss}}$  algorithms in Fig. 14 in $\mathrm{Z} \rightarrow \mu {}\mu$  data and MC simulation. The data and MC simulation agree within the assigned uncertainties for both algorithms. The CST $E_{\text {T}}^{\text {miss}}$ distribution in Fig. 14a has a very narrow core for the $\mathrm{Z} \rightarrow \mu {}\mu$  process, having 97% of data events with 1.03$\cdot$ $E_{\text {T}}^{\text {miss}}$ /$\sqrt{\Sigma E_{\mathrm {T}}}$ < 2. The proxy of the $E_{\text {T}}^{\text {miss}}$  significance, therefore, provides discrimination power between events with intrinsic $E_{\text {T}}^{\text {miss}}$ (e.g. $t\bar{t}$  and dibosons) and those with fake $E_{\text {T}}^{\text {miss}}$ (e.g. poorly measured $\mathrm{Z} \rightarrow \mu {}\mu$  events with a large number of jets).

The TST $E_{\text {T}}^{\text {miss}}$ is shown as an example of a pileup-suppressing algorithm. The $\Sigma E_{\mathrm {T}}$  is not always an accurate reflection of the resolution when there are significant contributions from tracking resolution, as discussed in Sect. 5.1. In particular, the performance of the TST reconstruction algorithm is determined by the tracking resolution, which is generally more precise than the calorimeter energy measurements because of the reduced pileup dependence, especially for charged particles with lower ${p}_{\text {T}}$ . Neutral particles are not included in the $\Sigma E_{\mathrm {T}}$ for the Track $E_{\text {T}}^{\text {miss}}$ and TST algorithms, but they do affect the resolution. In addition, a very small number of tracks do have very large over-estimated momentum measurements due to multiple scattering or other effects in the detector, and the momentum uncertainties of these tracks are not appropriately accounted for in the $\Sigma E_{\mathrm {T}}$ methodology.

### Tails of $E_{\text {T}}^{\text {miss}}$  distributions

Many analyses require large $E_{\text {T}}^{\text {miss}}$  to select events with high-${p}_{\text {T}}$ weakly interacting particles. The selection efficiency, defined as the number of events with $E_{\text {T}}^{\text {miss}}$ above a given threshold divided by the total number of events, is used to compare the performance of various $E_{\mathrm {T}}^{\mathrm {miss}}$ reconstruction algorithms. As $\mathrm{Z} \rightarrow \ell{}\ell$  events very rarely include high-$p_{\text {T}}$ neutrinos, they can be rejected by requiring substantial $E_{\text {T}}^{\text {miss}}$ . For events with intrinsic $E_{\text {T}}^{\text {miss}}$  such as $W\rightarrow \ell {}\nu$, higher selection efficiencies than the $\mathrm{Z} \rightarrow \ell{}\ell$  events are expected when requiring reconstructed $E_{\text {T}}^{\text {miss}}$ . For both cases, it is important to evaluate the performance of the reconstructed $E_{\mathrm {T}}^{\mathrm {miss}}$.

**Fig. 15:**
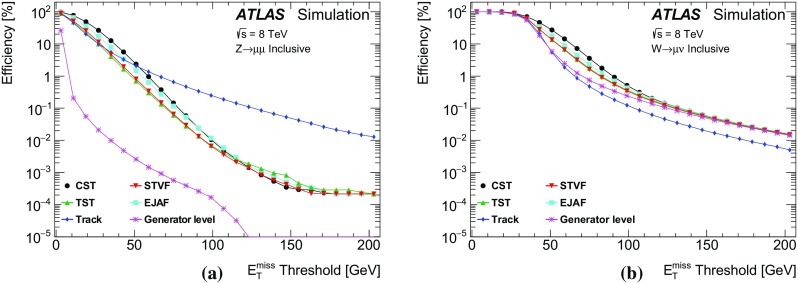
The selection efficiency is shown versus the $E_{\text {T}}^{\text {miss}}$  threshold for **a** $\mathrm{Z} \rightarrow \mu {}\mu$  and **b** $W\rightarrow \mu {}v$  inclusive MC simulation events

The selection efficiencies with various $E_{\text {T}}^{\text {miss}}$  algorithms are compared for simulated $\mathrm{Z} \rightarrow \mu {}\mu$  and $W\rightarrow \mu {}v$  processes as shown in Fig. 15 using the MC simulation. The event selections discussed in Sects. 3.2 and 3.3 are applied except the requirements on $E_{\mathrm {T}}^{\mathrm {miss}}$ and $m_{\mathrm {T}}$ for the $W\rightarrow \mu {}v$ selection.

As shown in Fig. 15a, the selection efficiency for $\mathrm{Z} \rightarrow \mu {}\mu$  events is around 1% for $E_{\text {T}}^{\text {miss}}$  > 50 $\text {GeV}$, for all $E_{\text {T}}^{\text {miss}}$  algorithms. Thus a $E_{\text {T}}^{\text {miss}}$ threshold requirement can be used to reject a large number of events without intrinsic $E_{\text {T}}^{\text {miss}}$ . However, the $E_\mathrm{T}^\mathrm{miss,True}$, which does not include detector resolution effects, shows the selection efficiency under ideal conditions, indicating there may be additional potential for improvement of the reconstructed $E_{\text {T}}^{\text {miss}}$ . Namely, the selection efficiency with $E_\mathrm{T}^\mathrm{miss,True}$ provides a benchmark against which to evaluate the performance of different $E_{\text {T}}^{\text {miss}}$  algorithms. The STVF, TST, and Track $E_{\mathrm {T}}^{\mathrm {miss}}$ distributions have narrow cores, so for $E_{\text {T}}^{\text {miss}}$  threshold $\lesssim$ 50 $\text {GeV}$ these three $E_{\text {T}}^{\text {miss}}$  definitions have the lowest selection efficiencies for $\mathrm{Z} \rightarrow \mu {}\mu$  events. Above 50 $\text {GeV}$, the Track $E_{\mathrm {T}}^{\mathrm {miss}}$ performance is degraded as a result of missing neutral particles, which gives it a very high selection efficiency. The TST and STVF $E_{\text {T}}^{\text {miss}}$  algorithms continue to have the lowest selection efficiency up to $E_{\text {T}}^{\text {miss}}$  threshold $\approx$ 110 $\text {GeV}$. For 110–160 $\text {GeV}$, the TST $E_{\text {T}}^{\text {miss}}$ has a longer tail than the CST $E_{\text {T}}^{\text {miss}}$ , which is a result of mismeasured low-${p}_{\text {T}}$  particles that scatter and are reconstructed as high-${p}_{\text {T}}$ tracks. Such mismeasurements^8^ are rare but significant in the $E_{\text {T}}^{\text {miss}}$  tail. The TST, STVF, CST, and EJAF $E_{\text {T}}^{\text {miss}}$  algorithms provide similar selection efficiencies for $E_{\text {T}}^{\text {miss}}$  > 160 $\text {GeV}$. Above this threshold, the $E_{\text {T}}^{\text {miss}}$ is dominated by mismeasured high-${p}_{\text {T}}$ physics objects which are identical in all object-based $E_{\text {T}}^{\text {miss}}$  definitions. Hence, the events with $E_{\text {T}}^{\text {miss}}$  $\gtrsim$ 160 $\text {GeV}$ are correlated among the TST, STVF, CST, and EJAF $E_{\text {T}}^{\text {miss}}$  distributions.

Figure 15b shows the selection efficiency for the $W\rightarrow \mu {}v$  simulated events passing a $E_{\text {T}}^{\text {miss}}$  threshold for all $E_{\mathrm {T}}^{\mathrm {miss}}$ algorithms. Requiring the $W\rightarrow \mu {}v$  events to pass the $E_{\text {T}}^{\text {miss}}$ threshold should ideally have a high selection efficiency similar to that of the $E_\mathrm{T}^\mathrm{miss,True}$. The CST $E_{\text {T}}^{\text {miss}}$  algorithm gives the highest selection efficiency between 30–120 $\text {GeV}$ but does not agree as well as that of the other $E_{\text {T}}^{\text {miss}}$  algorithms with the $E_\mathrm{T}^\mathrm{miss,True}$ selection efficiency for $E_{\text {T}}^{\text {miss}}$  threshold $\lesssim$ 110 $\text {GeV}$. This comes from the positive-definite nature of the $E_{\mathrm {T}}^{\mathrm {miss}}$ and the worse resolution of the CST $E_{\text {T}}^{\text {miss}}$  relative to the other $E_{\text {T}}^{\text {miss}}$  definitions. The Track $E_{\text {T}}^{\text {miss}}$  has the efficiency closest to that of the $E_\mathrm{T}^\mathrm{miss,True}$, but for Track $E_{\text {T}}^{\text {miss}}$  $\gtrsim$ 60 $\text {GeV}$, the amount of jet activity increases, which results in a lower selection efficiency because of missing neutral particles. The EJAF, STVF, and TST $E_{\text {T}}^{\text {miss}}$  distributions are closer than the CST to the $E_\mathrm{T}^\mathrm{miss,True}$ selection efficiency for $E_{\text {T}}^{\text {miss}}$  threshold $\lesssim$ 100 $\text {GeV}$, but the efficiencies for all the object-based algorithms and $E_\mathrm{T}^\mathrm{miss,True}$ converge for $E_{\text {T}}^{\text {miss}}$  threshold $\gtrsim$ 110 $\text {GeV}$. Hence, for large $E_{\text {T}}^{\text {miss}}$ all object-based algorithms perform similarly.

**Fig. 16:**
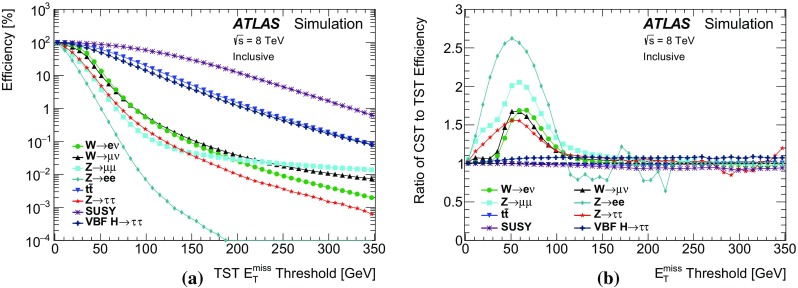
**a** The selection efficiency with TST $E_{\mathrm {T}}^{\mathrm {miss}}$ versus the $E_{\mathrm {T}}^{\mathrm {miss}}$ threshold and **b** the ratio of CST to TST efficiencies versus $E_{\text {T}}^{\text {miss}}$ threshold. In both cases, results are shown for several processes

In Fig. 16, selection efficiencies are shown as a function of the $E_{\text {T}}^{\text {miss}}$  threshold requirement for various simulated physics processes defined in Sect. 3.4 with no lepton, jet, or $m_{\mathrm {T}}$ threshold requirements. The physics object and event selection criteria are not applied in order to show the selection efficiency resulting from the $E_{\text {T}}^{\text {miss}}$  threshold requirement without biases in the event topology from the ATLAS detector acceptance for leptons or jets. Only the efficiencies for the CST and TST $E_{\text {T}}^{\text {miss}}$  distributions are compared for brevity. In Fig. 16a, the efficiencies with the TST $E_{\text {T}}^{\text {miss}}$  selection are shown. Comparing the physics processes while imposing a moderate $E_{\text {T}}^{\text {miss}}$  threshold requirement of $\sim$100 $\text {GeV}$ results in a selection efficiency of 60% for an ATLAS search for gluino-pair production [53], which is labelled as “SUSY”. The VBF $H \rightarrow \tau \tau$  and $t\bar{t}$  events are also selected with high efficiencies of 14 and 20%, respectively. With the 100 $\text {GeV}$ $E_{\text {T}}^{\text {miss}}$  threshold the selection efficiencies for these processes are more than an order of magnitude higher than those for leptonically decaying *W* bosons and more than two orders of magnitude higher than for *Z* boson events.

The $Z\rightarrow e e$ events have a lower selection efficiency (around 20 times lower at $E_{\text {T}}^{\text {miss}}$  $=$ 100 $\text {GeV}$) than the $\mathrm{Z} \rightarrow \mu {}\mu$  events. This is due to the muon tracking coverage, which is limited to $|\eta |$ < 2.7, whereas the calorimeter covers $|\eta |$ < 4.9. Muons behave as minimum-ionizing particles in the ATLAS calorimeters, so they are not included in the $E_{\text {T}}^{\text {miss}}$  outside the muon spectrometer acceptance. The electrons on the other hand are measured by the forward calorimeters. The electron and muon decay modes of the *W* boson have almost identical selection efficiencies at $E_{\text {T}}^{\text {miss}}$  $=$ 100 $\text {GeV}$ because there is $E_\mathrm{T}^\mathrm{miss,True}$ from the neutrino. However, the differences in selection efficiency are around a factor of four higher for $W\rightarrow \mu {}v$  than for $W\rightarrow e{}v$  at $E_{\text {T}}^{\text {miss}}$  $=$ 350 $\text {GeV}$. Over the entire $E_{\text {T}}^{\text {miss}}$  spectrum, the differences between the electron and muon final states for *W* bosons are smaller than that for *Z* bosons because there is a neutrino in $W\rightarrow \ell {}\nu$ events as opposed to none in the $\mathrm{Z} \rightarrow \ell{}\ell$ final state.

In Fig. 16b, the selection efficiencies for CST $E_{\text {T}}^{\text {miss}}$  threshold requirements are divided by those obtained using the TST $E_{\text {T}}^{\text {miss}}$. The selection efficiencies resulting from CST $E_{\text {T}}^{\text {miss}}$  thresholds for SUSY, $t\bar{t}$ , and VBF $H \rightarrow \tau \tau$ are within 10% of the efficiencies obtained using the TST $E_{\text {T}}^{\text {miss}}$ . For $E_{\text {T}}^{\text {miss}}$  thresholds from 40–120 $\text {GeV}$, the selection efficiencies for *W* and *Z* boson events are higher by up to 60–160% for CST $E_{\text {T}}^{\text {miss}}$  than TST $E_{\text {T}}^{\text {miss}}$ , which come from pileup contributions broadening the CST $E_{\text {T}}^{\text {miss}}$  distribution. The $Z \rightarrow \mu {}\mu$  and $Z\rightarrow e e$ events, which have no $E_\mathrm{T}^\mathrm{miss,True}$, show an even larger increase of 2.6 times as many $Z\rightarrow e e$ events passing a $E_{\text {T}}^{\text {miss}}$  threshold of 50 $\text {GeV}$. The increase is not as large for $Z \rightarrow \mu {}\mu$  as $Z\rightarrow e e$ events because neither $E_{\text {T}}^{\text {miss}}$  algorithm accounts for forward muons ($|\eta |$ > 2.7) as discussed above. Moving to a higher $E_{\text {T}}^{\text {miss}}$  threshold, mismeasured tracks in the TST algorithm cause it to select more $Z\rightarrow e e$ events with 120 < $E_{\text {T}}^{\text {miss}}$  < 230 $\text {GeV}$. In addition, the CST $E_{\text {T}}^{\text {miss}}$  also includes electron energy contributions (${p}_{\text {T}}$  < 20 $\text {GeV}$) in the forward calorimeters ($|\eta |$ > 3.1) that the TST does not.

The CST and TST $E_{\text {T}}^{\text {miss}}$  distributions agree within 10% in selection efficiency for $E_{\text {T}}^{\text {miss}}$  > 250 $\text {GeV}$ for all physics processes shown. This demonstrates a strong correlation between the $E_{\text {T}}^{\text {miss}}$  distributions for events with large $E_\mathrm{T}^\mathrm{miss,True}$, or a strong correlation between the physics objects that cause a large mismeasurement in $E_{\text {T}}^{\text {miss}}$  for *Z* events.

### Correlation of fake $E_{\text {T}}^{\text {miss}}$  between algorithms

**Fig. 17:**
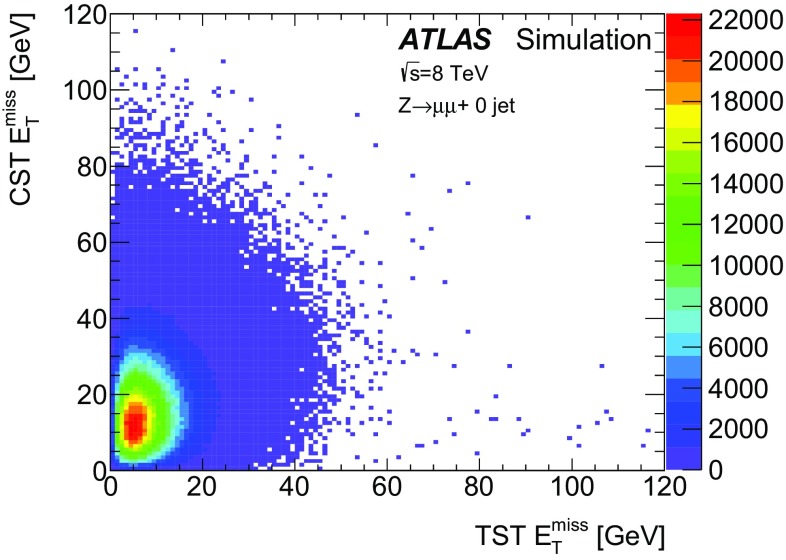
The CST $E_{\mathrm {T}}^{\mathrm {miss}}$ versus the TST $E_{\mathrm {T}}^{\mathrm {miss}}$ in $\mathrm{Z} \rightarrow \mu {}\mu$  $+$ 0-jet events from the MC simulation. The vector correlation coefficient is 0.177 [54]

The tracking and the calorimeters provide almost completely independent estimates of the $E_{\text {T}}^{\text {miss}}$ . These two measurements complement each other, and the $E_{\text {T}}^{\text {miss}}$  algorithms discussed in this paper combine that information in different ways. The distribution of the TST $E_{\text {T}}^{\text {miss}}$  versus the CST $E_{\text {T}}^{\text {miss}}$  is shown for the simulated 0-jet $\mathrm{Z} \rightarrow \mu {}\mu$  sample in Fig. 17. This figure shows the correlation of fake $E_{\text {T}}^{\text {miss}}$  between the two algorithms, which originates from many sources including incorrect vertex association and miscalibration of high-${p}_{\text {T}}$ physics objects.

Vector correlation coefficients [54], shown in Table 5, are used to estimate the correlation between the $E_{\text {T}}^{\text {miss}}$  distributions resulting from different reconstruction algorithms. The value of the vector correlation coefficients ranges from 0 to 2, with 0 being the least correlated and 2 being the most correlated. The coefficients shown are obtained using the simulated 0-jet and inclusive $Z \rightarrow \mu {}\mu$  MC samples. The least-correlated $E_{\text {T}}^{\text {miss}}$  distributions are the CST and Track $E_{\text {T}}^{\text {miss}}$ , which use mostly independent momenta measurements in their reconstructions. The correlations of the other $E_{\text {T}}^{\text {miss}}$  distributions to the CST $E_{\text {T}}^{\text {miss}}$  decrease as more tracking information is used to suppress the pileup dependence of the soft term, with the TST $E_{\text {T}}^{\text {miss}}$ distribution having the second smallest vector correlation coefficient with respect to the CST $E_{\text {T}}^{\text {miss}}$ distribution. Placing requirements on a combination of $E_{\text {T}}^{\text {miss}}$ distributions or requiring the difference in azimuthal direction between two $E_{\text {T}}^{\text {miss}}$ vectors to be small can greatly reduce fake $E_{\text {T}}^{\text {miss}}$  backgrounds, especially using the least-correlated $E_{\text {T}}^{\text {miss}}$  distributions. Such strategies are adopted in several Higgs boson analyses in ATLAS [55–57].

**Table 5:** Vector correlation coefficients are shown between $E_{\text {T}}^{\text {miss}}$  definitions in $\mathrm{Z} \rightarrow \mu {}\mu$  MC simulation. Below the diagonal are events in the 0-jet sample, and above the diagonal are inclusive events

$E_{\text {T}}^{\text {miss}}$	CST	TST	Track	STVF	EJAF
CST	2	0.261	0.035	0.525	0.705
TST	0.177	2	0.232	1.557	0.866
Track	0.153	1.712	2	0.170	0.065
STVF	0.585	1.190	1.017	2	1.256
EJAF	0.761	0.472	0.401	1.000	2

## Jet-${p}_{\text {T}}$  threshold and vertex association selection

Jets can originate from pileup interactions, so tracks matched to the jets are extrapolated back to the beamline to ascertain whether they are consistent with originating from the hard scatter or a pileup collision. The JVF defined in Sect. 4.1.1 is used to separate pileup jets and jets from the hard scatter. The STVF, EJAF, and TST $E_{\text {T}}^{\text {miss}}$  algorithms improve their jet identification by removing jets associated with pileup vertices or jets that have a large degradation in momentum resolution due to pileup activity. Energy contributions from jets not associated with the hard-scatter vertex are included in the soft term. For the TST, this means that charged particles from jets not associated with the hard-scatter vertex may then enter the soft term if their position along the beamline is consistent with the *z*-position of the hard-scatter vertex.

**Fig. 18:**
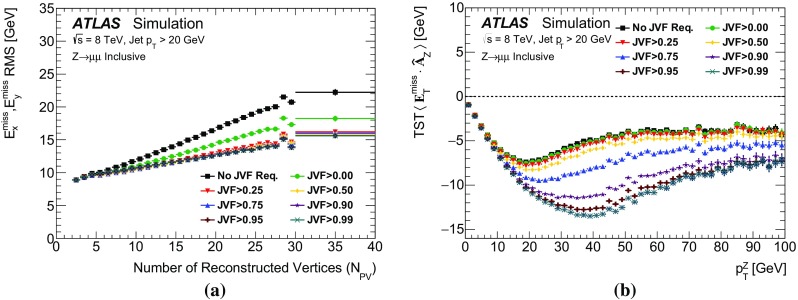
The **a** TST $E_{\text {T}}^{\text {miss}}$ resolution versus the number of reconstructed vertices per bunch crossing (${N}_{\mathrm {PV}}$) and the **b** TST $\vec {E}_{{\mathrm{T}}}^{\mathrm{miss}}$ in the direction of the $\vec {p}_{\mathrm {T}}^{Z}\;$ are shown for the different JVF selection criterion values applied to jets with $p_{\text {T}}$  > 20 $\text {GeV}$ and $|\eta |$ < 2.4 using the $Z \rightarrow {\mu \mu }$ simulation

Applying a JVF cut is a trade-off between removing jets from pileup interactions and losing jets from the hard scatter. Therefore, several values of the JVF selection criterion are considered in $\mathrm{Z} \rightarrow \ell{}\ell$ events with jets having $p_{\text {T}}$  > 20 $\text {GeV}$; their impact on the $E_{\text {T}}^{\text {miss}}$  resolution and scale is investigated in Fig. 18. Larger JVF thresholds on jets reduce the pileup dependence of the $E_{\text {T}}^{\text {miss}}$ resolution, but they simultaneously worsen the $E_{\text {T}}^{\text {miss}}$ scale. Thus the best compromise for the value of the JVT threshold is chosen. Requiring JVF > 0.25 greatly improves the stability of the $E_{\text {T}}^{\text {miss}}$ resolution with respect to pileup by reducing the dependence of the $E_{\text {T}}^{\text {miss}}$  resolution on the number of reconstructed vertices as shown in Fig. 18a. The $\vec {E}_{{\mathrm{T}}}^{\mathrm{miss}}$ in $\mathrm{Z} \rightarrow \ell{}\ell$ events ideally has a magnitude of zero, apart from some relatively infrequent neutrino contributions in jets. So its magnitude should be consistently zero along any direction. The $\vec {p}_{\mathrm {T}}^{Z}\;$ remains unchanged for different JVF requirements, which makes its direction a useful reference to check the calibration of the $\vec {E}_{{\mathrm{T}}}^{\mathrm{miss}}$. The difference from zero of the average value of the reconstructed $E_{\text {T}}^{\text {miss}}$ along $\vec {p}_{\mathrm {T}}^{Z}\;$ increases as tighter JVF selections are applied as shown in Fig. 18b. Requiring a JVF threshold of 0.25 or higher slightly improves the stability of the resolution with respect to pileup, whereas it visibly degrades the $E_{\text {T}}^{\text {miss}}$ response by removing too many hard-scatter jets. Lastly, pileup jets with ${p}_{\text {T}}$  > 50 $\text {GeV}$ are very rare [4], so applying the JVF requirement above this ${p}_{\text {T}}$  threshold is not useful. Therefore, requiring JVF to be larger than 0.25 for jets with $p_{\text {T}}$  < 50 $\text {GeV}$ within the tracking volume ($|\eta |$ < 2.4) is the preferred threshold for the $E_{\text {T}}^{\text {miss}}$  reconstruction.

**Fig. 19:**
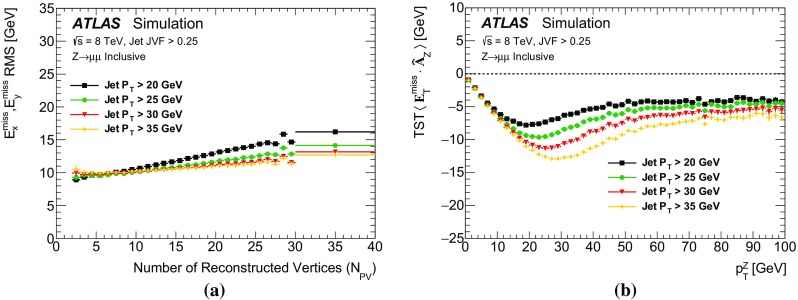
The **a** TST $E_{\text {T}}^{\text {miss}}$ resolution as a function of the number of reconstructed vertices per bunch crossing (${N}_{\mathrm {PV}}$) and the **b** TST $\vec {E}_{{\mathrm{T}}}^{\mathrm{miss}}$ in the direction of the $\vec {p}_{\mathrm {T}}^{Z}\;$ are shown for different jet-${p}_{\text {T}}$ thresholds using the $Z \rightarrow {\mu \mu }$ simulation. JVF > 0.25 is required for all jets with $p_{\text {T}}$  > 20 $\text {GeV}$ and $|\eta |$ < 2.4

In addition, the ${p}_{\text {T}}$  threshold, which defines the boundary between the jet and soft terms, is optimized. For these studies, the jets with $p_{\text {T}}$  > 20 $\text {GeV}$ and $|\eta |$ < 2.4 are required to have JVF > 0.25. A procedure similar to that used for the JVF optimization is used for the jet-${p}_{\text {T}}$  threshold using the same two metrics as shown in Figure 19. While applying a higher ${p}_{\text {T}}$  threshold improves the $E_{\text {T}}^{\text {miss}}$  resolution versus the number of pileup vertices, by decreasing the slope, the $\vec {E}_{{\mathrm{T}}}^{\mathrm{miss}}$ becomes strongly biased in the direction opposite to the $\vec {p}_{\mathrm {T}}^{Z}\;$. Therefore, the ${p}_{\text {T}}$  threshold of 20 $\text {GeV}$ is preferred.

## Systematic uncertainties of the soft term

The $\vec {E}_{{\mathrm{T}}}^{\mathrm{miss}}$ is reconstructed from the vector sum of several terms corresponding to different types of contributions from reconstructed physics objects, as defined in Eq. (2). The estimated uncertainties in the energy scale and momentum resolution for the electrons [14], muons [13], jets [44], $\tau _{\mathrm{had}{\text {-}}\mathrm{vis}}$ [47], and photons [14] are propagated into the $E_{\text {T}}^{\text {miss}}$ . This section describes the estimation of the systematic uncertainties for the $E_{\text {T}}^{\text {miss}}$ soft term. These uncertainties take into account the impact of the generator and underlying-event modelling used by the ATLAS Collaboration, as well as effects from pileup.

The balance of the soft term with the calibrated physics objects is used to estimate the soft-term systematic uncertainties in $\mathrm{Z} \rightarrow \mu {}\mu$  events, which have very little $E_\mathrm{T}^\mathrm{miss,True}$. The transverse momenta of the calibrated physics objects, $\vec {p}_{\mathrm T}^\text {\ hard}$ , is defined as

15 $$\begin{aligned} \vec {p}_{\mathrm T}^\text {\ hard} = \sum \vec {p}_\mathrm{T}^{\ e} + \sum \vec {p}_\mathrm{T}^{\ \mu } + \sum \vec {p}_\mathrm{T}^{\ \gamma } + \sum \vec {p}_\mathrm{T}^{\ \tau } + \sum \vec {p}_\mathrm{T}^{\text {\ jet}} {}, \end{aligned}$$

which is the vector sum of the transverse momenta of the high-${p}_{\text {T}}$ physics objects. It defines an axis (with unit vector $\hat{p}_{\mathrm T}^\text {\ hard}$ ) in the transverse plane of the ATLAS detector along which the $E_{\text {T}}^{\text {miss}}$  soft term is expected to balance $p_{\mathrm {T}}^\mathrm{{hard}}$ in $\mathrm{Z} \rightarrow \mu {}\mu$  events. This balance is sensitive to the differences in calibration and reconstruction of the $E_\mathrm{T}^{\mathrm {miss,soft}}$ between data and MC simulation and thus is sensitive to the uncertainty in the soft term. This discussion is similar to the one in Sect. 6.2; however, here the soft term is compared to the hard term rather than comparing the $\vec {E}_{{\mathrm{T}}}^{\mathrm{miss}}$ to the recoil of the *Z*.

### Methodology for CST

Two sets of systematic uncertainties are considered for the CST. The same approach is used for the STVF and EJAF algorithms to evaluate their soft-term systematic uncertainties. The first approach decomposes the systematic uncertainties into the longitudinal and transverse components along the direction of $\vec {p}_{\mathrm T}^\text {\ hard}$ , whereas the second approach estimates the global scale and resolution uncertainties. While both methods were recommended for analyses of the 8 $\text {TeV}$ dataset, the first method, described in Sect. 8.1.1, gives smaller uncertainties. Therefore, the second method, which is discussed in Sect. 8.1.2, is now treated as a cross-check.

Both methods consider a subset of $\mathrm{Z} \rightarrow \mu {}\mu$  events that do not have any jets with $p_{\text {T}}$ > 20 $\text {GeV}$ and $|\eta |$ < 4.5. Such an event topology is optimal for estimation of the soft-term systematic uncertainties because only the muons and the soft term contribute to the $E_{\text {T}}^{\text {miss}}$. In principle the methods are valid in event topologies with any jet multiplicity, but the $\mathrm{Z} \rightarrow \mu {}\mu$ $+\ge$1-jet events are more susceptible to jet-related systematic uncertainties.

#### Evaluation of balance between the soft term and the hard term

The primary or “balance” method exploits the momentum balance in the transverse plane between the soft and hard terms in $\mathrm{Z} \rightarrow \ell{}\ell$  events, and the level of disagreement between data and simulation is assigned as a systematic uncertainty.

The $\vec {E}_\mathrm{T}^{\mathrm {\ miss,soft}}$ is decomposed along the $\hat{p}_{\mathrm T}^\text {\ hard}$ direction. The direction orthogonal to $\hat{p}_{\mathrm T}^\text {\ hard}$ is referred to as the perpendicular direction while the component parallel to $\hat{p}_{\mathrm T}^\text {\ hard}$  is labelled as the longitudinal direction. The projections of $\vec {E}_\mathrm{T}^{\mathrm {\ miss,soft}}$ along those directions are defined as:

16 $$\begin{aligned} \begin{array}{r@{}l} E_{\parallel }^{\mathrm {miss,soft}}&{}{}= E_\mathrm{T}^{\mathrm {miss,soft}}{} \cos \phi (\vec {E}_\mathrm{T}^{\mathrm {\ miss,soft}}{},\vec {p}_{\mathrm T}^\text {\ hard} {}), \\ E_{\perp }^{\mathrm {miss,soft}}&{}{}= E_\mathrm{T}^{\mathrm {miss,soft}}{} \sin \phi (\vec {E}_\mathrm{T}^{\mathrm {\ miss,soft}}{},\vec {p}_{\mathrm T}^\text {\ hard} {}), \end{array} \end{aligned}$$

**Fig. 20:**
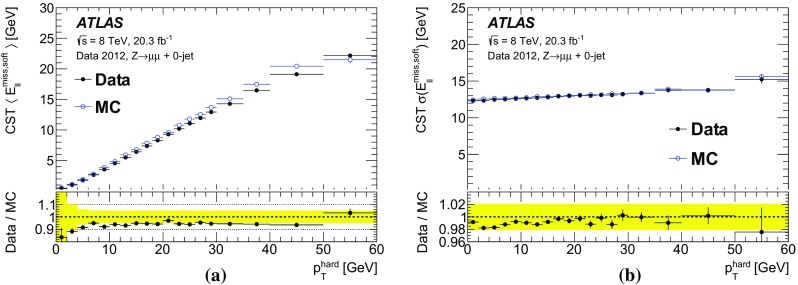
The **a** mean and **b** Gaussian width of the CST $\vec {E}_{{\mathrm{T}}}^{\mathrm{miss}}$ projected onto $\hat{p}_{\mathrm T}^\text {\ hard}$  are each shown as a function of $p_{\mathrm {T}}^\mathrm{{hard}}$ in $\mathrm{Z} \rightarrow \mu {}\mu$ $+$0-jet events. The ratio of data to MC simulation is shown in the *lower portion* of the plot with the band representing the assigned systematic uncertainty

The $E_{\parallel }^{\mathrm {miss,soft}}$ is sensitive to scale and resolution differences between the data and simulation because the soft term should balance the $\vec {p}_{\mathrm T}^\text {\ hard}$ in $\mathrm{Z} \rightarrow \mu {}\mu$  events. For a narrow range of $p_{\mathrm {T}}^\mathrm{{hard}}$ values, the mean and width of the $E_{\parallel }^{\mathrm {miss,soft}}$ are compared between data and MC simulation. On the other hand, the perpendicular component, $E_{\perp }^{\mathrm {miss,soft}}$, is only sensitive to differences in resolution. A Gaussian function is fit to the $\vec {E}_{{\mathrm{T}}}^{\mathrm{miss}}$ projected onto $\hat{p}_{\mathrm T}^\text {\ hard}$ in bins of $p_{\mathrm {T}}^\mathrm{{hard}}$, and the resulting Gaussian mean and width are shown in Fig. 20. The mean increases linearly with $p_{\mathrm {T}}^\mathrm{{hard}}$, because the soft term is not calibrated to the correct energy scale. On the other hand, the width is relatively independent of $p_{\mathrm {T}}^\mathrm{{hard}}$, because the width is mostly coming from pileup contributions.

The small discrepancies in mean and width between data and simulation are taken as the systematic uncertainties for the scale and resolution, respectively. A small dependence on the average number of collisions per bunch crossing is observed for the scale and resolution uncertainties for high $p_{\mathrm {T}}^\mathrm{{hard}}$, so the uncertainties are computed in three ranges of pileup and three ranges of $p_{\mathrm {T}}^\mathrm{{hard}}$. The scale uncertainty varies from $-0.4$ to 0.3 $\text {GeV}$ depending on the bin, which reduces the uncertainties from the 5% shown in Fig. 20 for $p_{\mathrm {T}}^\mathrm{{hard}}$ > 10 $\text {GeV}$. A small difference in the uncertainties for the resolution along the longitudinal and perpendicular directions is observed, so they are considered separately. The average uncertainty is about 2.1% (1.8%) for the longitudinal (perpendicular) direction.

#### Cross-check method for the CST systematic uncertainties

**Fig. 21:**
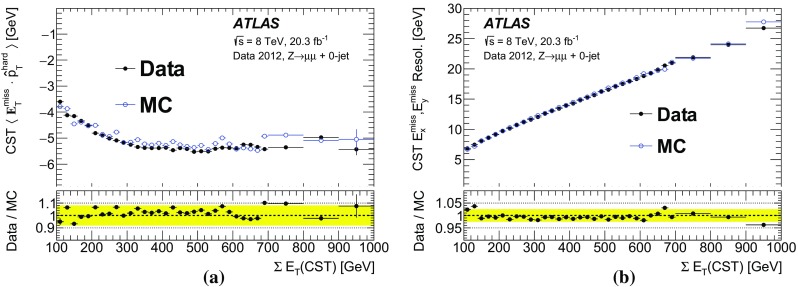
The **a** projection of CST $\vec {E}_{{\mathrm{T}}}^{\mathrm{miss}}$ onto $\hat{p}_{\mathrm T}^\text {\ hard}$ and **b** the Gaussian width (resol.) of the combined distribution of CST $E_\mathrm{x}^\mathrm{miss}$ and $E_\mathrm{y}^\mathrm{miss}$ are shown versus $\Sigma E_{\mathrm {T}}$ (CST). The ratio of data to MC simulation is shown in the lower portion of the plot with the *solid band* representing the assigned systematic uncertainty

As a cross-check of the method used to estimate the CST uncertainties, the sample of $\mathrm{Z} \rightarrow \mu {}\mu$ $+$0-jet events is also used to evaluate the level of agreement between data and simulation. The projection of the $\vec {E}_{{\mathrm{T}}}^{\mathrm{miss}}$ onto $\hat{p}_{\mathrm T}^\text {\ hard}$  provides a test for potential biases in the $E_{\text {T}}^{\text {miss}}$  scale. The systematic uncertainty in the soft-term scale is estimated by comparing the ratio of data to MC simulation for $\langle \vec {E}_{{\mathrm{T}}}^{\mathrm{miss}}\cdot \hat{p}_{\mathrm T}^\text {\ hard} \rangle$ versus $\Sigma E_{\mathrm {T}}$ (CST) as shown in Fig. 21a. The average deviation from unity in the ratio of data to MC simulation is about 8%, which is taken as a flat uncertainty in the absolute scale. The systematic uncertainty in the soft-term resolution is estimated by evaluating the level of agreement between data and MC simulation in the $E_\mathrm{x}^\mathrm{miss}$ and $E_\mathrm{y}^\mathrm{miss}$ resolution as a function of the $\Sigma E_{\mathrm {T}}$ (CST) (Fig. 21b). The uncertainty on the soft-term resolution is about 2.5% and is shown as the band in the data/MC ratio.

Even though the distributions appear similar, the results in this section are derived by projecting the full $E_{\text {T}}^{\text {miss}}$  onto the $\hat{p}_{\mathrm T}^\text {\ hard}$  in the 0-jet events, and are not directly comparable to the ones in Sect. 8.1.1, in which only the soft term is projected onto $\hat{p}_{\mathrm T}^\text {\ hard}$ .

### Methodology for TST and Track $E_{\text {T}}^{\text {miss}}$

A slightly different data-driven methodology is used to evaluate the systematic uncertainties in the TST and Track $E_{\text {T}}^{\text {miss}}$ . Tracks matched to jets that are included in the hard term are removed from the Track $E_{\text {T}}^{\text {miss}}$  and are treated separately, as described in Sect. 8.2.3.

The method exploits the balance between the soft track term and $\vec {p}_{\mathrm T}^\text {\ hard}$ and is similar to the balance method for the CST. The systematic uncertainties are split into two components: the longitudinal ($E_{\parallel }^{\mathrm {miss,soft}}$) and transverse ($E_{\perp }^{\mathrm {miss,soft}}$) projections onto $\vec {p}_{\mathrm T}^\text {\ hard}$ as defined in Eq. (16).

The $E_{\parallel }^{\mathrm {miss,soft}}$ in data is fit with the MC simulation convolved with a Gaussian function, and the fitted Gaussian mean and width are used to extract the differences between simulation and data. The largest fit values of the Gaussian width and offset define the systematic uncertainties. For the perpendicular component, the simulation is only smeared by a Gaussian function of width $\sigma _{\perp }$ to match the data. The mean, which is set to zero in the fit, is very small in data and MC simulation because the hadronic recoil only affects $E_{\parallel }^{\mathrm {miss,soft}}$. The fitting is done in 5 or 10 $\text {GeV}$ bins of $p_{\mathrm {T}}^\mathrm{{hard}}$ from 0–50 $\text {GeV}$, and a single bin for $p_{\mathrm {T}}^\mathrm{{hard}}$ > 50 $\text {GeV}$.

**Fig. 22:**
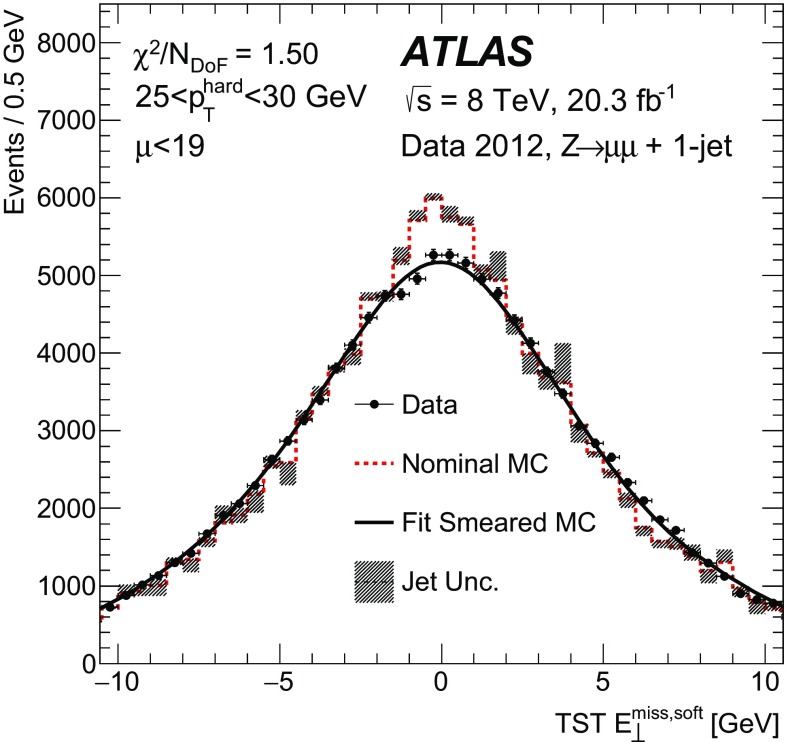
Fit to the TST $E_{\perp }^{\mathrm {miss,soft}}$ for $\mu$ < 19 and 25 < $p_{\mathrm {T}}^\mathrm{{hard}}$ < 30 $\text {GeV}$ in the 1-jet sample. The nominal MC simulation, the jet-related systematic uncertainties (*hashed band*), and the data are shown. The nominal MC simulation is convolved with a Gaussian function until it matches the data, and the resulting fit is shown with the *solid curve*. The jet counting for the 1-jet selection uses the same JVF criterion as the TST $E_{\text {T}}^{\text {miss}}$  reconstruction algorithm

An example fit is shown in Fig. 22 for illustration. The 1-jet selection with the JVF requirement is used to show that the differences between data and simulation, from the jet-related systematic uncertainties, are small relative to the differences in the soft-term modelling. The impact of the jet-related systematic uncertainties is less than 0.1% in the Gaussian smearing ($\sigma$ $=$ 1.61 $\text {GeV}$), indicating that the jet-related systematic uncertainties do not affect the extraction of the TST systematic uncertainties.

**Fig. 23:**
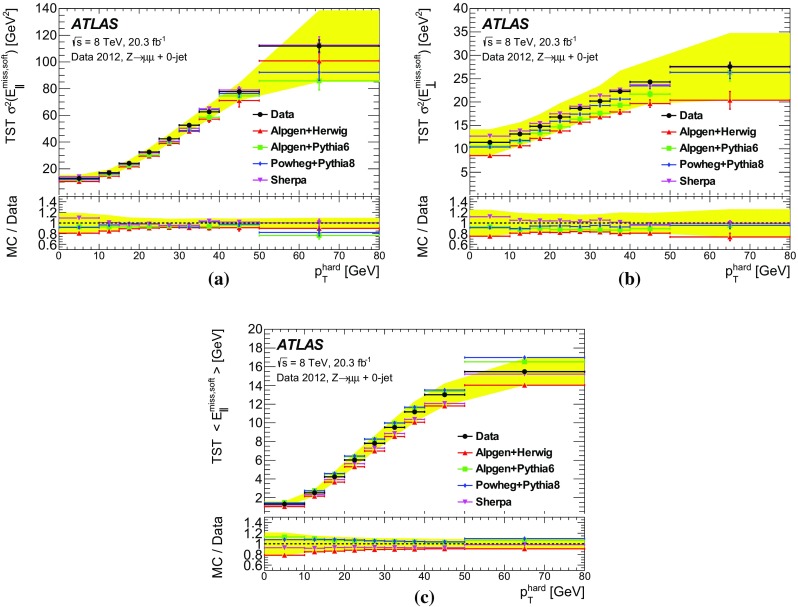
The fitted TST **a** $\sigma ^2(E_{\parallel }^{\mathrm {miss,soft}})$, **b** $\sigma ^2(E_{\perp }^{\mathrm {miss,soft}})$, and **c** $\langle E_{\parallel }^{\mathrm {miss,soft}}\rangle$ in each case versus $p_{\mathrm {T}}^\mathrm{{hard}}$ are shown in data and Alpgen $+$ Herwig , Powheg $+$Pythia8, Sherpa , and Alpgen $+$ Pythia  $\mathrm{Z} \rightarrow \mu {}\mu$ simulation. The *error bars* on the data and MC simulation points are the errors from the Gaussian fits. The *solid band*, which is centred on the data, shows the parameter’s systematic uncertainties from Table 6. The *insets* at the *bottom* of the figures show the ratios of the MC predictions to the data

The Gaussian width squared of $E_{\parallel }^{\mathrm {miss,soft}}$ and $E_{\perp }^{\mathrm {miss,soft}}$ components and the fitted mean of $E_{\parallel }^{\mathrm {miss,soft}}$ for data and MC simulation are shown versus $p_{\mathrm {T}}^\mathrm{{hard}}$ in Fig. 23. The systematic uncertainty squared of the convolved Gaussian width and the systematic uncertainty of the offset for the longitudinal component are shown in the bands. While the systematic uncertainties are applied to the MC simulation, the band is shown centred around the data to show that all MC generators plus parton shower models agree with the data within the assigned uncertainties. Similarly for the $E_{\perp }^{\mathrm {miss,soft}}$, the width of the convolved Gaussian function for the perpendicular component is shown in the band. The Alpgen+Herwig simulation has the largest disagreement with data, so the Gaussian smearing parameters and offsets applied to the simulation are used as the systematic uncertainties in the soft term. The $p_{\mathrm {T}}^\mathrm{{hard}}$ > 50 $\text {GeV}$ bin has the smallest number of data entries; therefore, it has the largest uncertainties in the fitted mean and width. In this bin of the distribution shown in Figure 23(a), the statistical uncertainty from the Alpgen $+$ Herwig simulation, which is not the most discrepant from data, is added to the uncertainty band, and this results in a systematic uncertainty band that spans the differences in MC generators for $\sigma ^2(E_{\parallel }^{\mathrm {miss,soft}})$ for events with $p_{\mathrm {T}}^\mathrm{{hard}}$ > 50 $\text {GeV}$.

The impact of uncertainties coming from the parton shower model, the number of jets, $\mu$ dependence, JER/JES uncertainties, and forward versus central jet differences was evaluated. Among the uncertainties, the differences between the generator and parton shower models have the most dominant effects. The total TST systematic uncertainty is summarized in Table 6.

**Table 6:** The TST scale ($\Delta _{\mathrm {TST}}$) and resolution uncertainties ($\sigma _{\parallel }$ and $\sigma _{\perp }$) are shown in bins of $p_{\mathrm {T}}^\mathrm{{hard}}$

$p_{\mathrm {T}}^\mathrm{{hard}}$ range ($\text {GeV}$)	$\Delta _{\mathrm {TST}}$ ($\text {GeV}$)	$\sigma _{\parallel }$ ($\text {GeV}$)	$\sigma _{\perp }$ ($\text {GeV}$)
0–10	0.3	1.6	1.7
10–15	0.4	1.6	1.6
15–20	0.6	1.6	1.6
20–25	0.7	1.8	1.7
25–30	0.8	1.9	1.7
30–35	1.0	2.1	1.8
35–40	1.1	2.4	2.1
40–50	1.2	2.6	2.2
>50	1.4	5.2	2.7

#### Propagation of systematic uncertainties

The CST systematic uncertainties from the balance method defined in Sect. 8.1.1 are propagated to the nominal $\vec {E}_\mathrm{T}^{\mathrm {\ miss,soft}}$ as follows:

17a $$\begin{aligned} E_{\parallel (\perp ), \mathrm{reso}}^{\mathrm {miss,soft}}= & {} (1 \pm R_{\parallel (\perp )})(E_{\parallel (\perp )}^{\mathrm {miss,soft}}- \langle E_{\parallel (\perp )}^{\mathrm {miss,soft}}\rangle ) + \langle E_{\parallel (\perp )}^{\mathrm {miss,soft}}\rangle \end{aligned}$$

17b $$\begin{aligned} E_{\parallel , \mathrm{scale}\pm }^{\mathrm {miss,soft}}= & {} E_{\parallel }^{\mathrm {miss,soft}}\pm \Delta _{\text {CST}} \end{aligned}$$

where $E_{\parallel (\perp ), \mathrm{reso}}^{\mathrm {miss,soft}}$ and $E_{\parallel , \mathrm{scale}\pm }^{\mathrm {miss,soft}}$ are the values after propagating the resolution and scale uncertainties, respectively, in the longitudinal (perpendicular) directions. The mean values of parameters are denoted using angled brackets. The $\Delta _{\text {CST}}$ is the scale uncertainty, and the $R_{\parallel (\perp )}$ is the fractional resolution uncertainty taken from the lower portion of Fig. 20b. Both depend on the $p_{\mathrm {T}}^\mathrm{{hard}}$ and the average number of pileup interactions per bunch crossing. Each propagation of the systematic uncertainties in Eq. (17b) is called a variation, and all of the variations are used in ATLAS analyses.

The systematic uncertainties in the resolution and scale for the CST using the cross-check method defined in Sect. 8.1.2 are propagated to the nominal $\vec {E}_\mathrm{T}^{\mathrm {\ miss,soft}}$ as follows:

18a $$\begin{aligned} E_{x(y), \mathrm {reso}}^{\mathrm {miss,soft}}= & {} E_{x(y)}^{\mathrm {miss,soft}}\cdot \text {Gaus}(1,\hat{\sigma }_{\text {CST}}), \end{aligned}$$

18b $$\begin{aligned} E_{x(y), \mathrm {scale}\pm }^{\mathrm {miss,soft}}= & {} E_{x(y)}^{\mathrm {miss,soft}}\cdot (1\pm \delta ), \end{aligned}$$

where $E_{x(y), \mathrm {reso}}^{\mathrm {miss,soft}}$ and $E_{x(y), \mathrm {scale}\pm }^{\mathrm {miss,soft}}$ are the values after propagating the resolution and scale uncertainties, respectively, in the *x* (*y*) directions. Here, $\delta$ is the fractional scale uncertainty, and $\hat{\sigma }_{\text {CST}}$ corrects for the differences in resolution between the data and simulation.

The systematic uncertainties in the resolution and scale for the TST $\vec {E}_\mathrm{T}^{\mathrm {\ miss,soft}}$ are propagated to the nominal $\vec {E}_\mathrm{T}^{\mathrm {\ miss,soft}}$ as follows:

19a $$\begin{aligned} E_{\parallel (\perp ), \mathrm{reso}}^{\mathrm {miss,soft}}= & {} E_{\parallel (\perp )}^{\mathrm {miss,soft}}+ \text {Gaus}(\Delta _{\text {TST}}, \sigma _{\parallel (\perp )}), \end{aligned}$$

19b $$\begin{aligned} E_{\parallel , \mathrm{scale}\pm }^{\mathrm {miss,soft}}= & {} E_{\parallel }^{\mathrm {miss,soft}}\pm \Delta _{\text {TST}} . \end{aligned}$$

The symbol $\text {Gaus}(\Delta _{\text {TST}} , \sigma _{\parallel (\perp )})$ represents a random number sampled from a Gaussian distribution with mean $\Delta _{\text {TST}}$ and width $\sigma _{\parallel (\perp )}$. The shift $\Delta _{\text {TST}}$ is zero for the perpendicular component. All of the TST systematic-uncertainty variations have a wider distribution than the nominal MC simulation, when the Gaussian smearing is applied. To cover cases in which the data have a smaller resolution (narrower distribution) than MC simulation, a downward variation is computed using Eq. (20). To compute the yield of predicted events in the variation, $Y_{\text {down}}(X)$, for a given value *X* of the $E_{\text {T}}^{\text {miss}}$ , the yield is defined as the

20 $$\begin{aligned} Y_{\text {down}}(X) = \frac{[Y(X)]^2}{Y_{\text {smeared}}(X)}, \end{aligned}$$

where the square of the yield of the nominal distribution, *Y*(*X*), is divided by the yield of events after applying the variation with Gaussian smearing to the kinematic variable, $Y_{\text {smeared}}(X)$. In practice, the yields are typically the content of histogram bins before (*Y*(*X*)) and after ($Y_{\text {smeared}}(X)$) the systematic uncertainty variations. This procedure can be applied to any kinematic observable by propagating only the smeared soft-term variation to the calculation of the kinematic observable *X* and then computing the yield $Y_{\text {down}}(X)$ as defined in Eq. (20).

There are six total systematic uncertainties associated with the TST:

- Increase scale ($$E_{\parallel , \mathrm{scale}+}^{\mathrm {miss,soft}}$$)
- Decrease scale ($$E_{\parallel , \mathrm{scale}-}^{\mathrm {miss,soft}}$$)
- Gaussian smearing of $E_{\parallel }^{\mathrm {miss,soft}}$ ($E_{\parallel , \mathrm{reso}}^{\mathrm {miss,soft}}$)
- The downward variation of the above $E_{\parallel , \mathrm{reso}}^{\mathrm {miss,soft}}$ computed using Eq. (20)
- Gaussian smearing of $E_{\perp }^{\mathrm {miss,soft}}$ ($E_{\perp , \mathrm{reso}}^{\mathrm {miss,soft}}$)
- The downward variation of the above $E_{\perp , \mathrm{reso}}^{\mathrm {miss,soft}}$ computed using Eq. (20)

#### Closure of systematic uncertainties

The systematic uncertainties derived in this section for the CST and TST $E_{\text {T}}^{\text {miss}}$ are validated by applying them to the $\mathrm{Z} \rightarrow \mu {}\mu$ sample to confirm that the differences between data and MC simulation are covered.

**Fig. 24:**
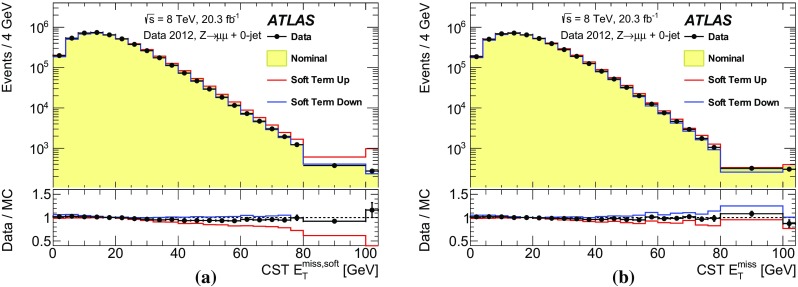
Distributions of **a** $E_\mathrm{T}^{\mathrm {miss,soft}}$ and **b** $E_{\mathrm {T}}^{\mathrm {miss}}$ with the CST algorithm. Data are compared to the nominal simulation distribution as well as those resulting from applying the shifts/smearing according to the scale and resolution systematic uncertainties on the $E_\mathrm{T}^{\mathrm {miss,soft}}$. The resulting changes from the variations are added in quadrature, and the *insets* at the *bottom* of the figures show the ratios of the data to the MC predictions. The uncertainties are estimated using the balance method described in Sect. 8.1.1

**Fig. 25:**
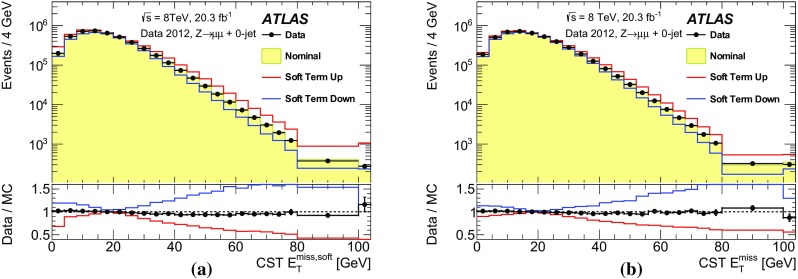
Distributions of **a** $E_\mathrm{T}^{\mathrm {miss,soft}}$ and **b** $E_{\mathrm {T}}^{\mathrm {miss}}$ with the CST algorithm. Data are compared to the nominal simulation distribution as well as those resulting from applying the shifts/smearing according to the scale and resolution systematic uncertainties on the $E_\mathrm{T}^{\mathrm {miss,soft}}$. The resulting changes from the variations are added in quadrature, and the *insets* at the *bottom* of the figures show the ratios of the data to the MC predictions. The uncertainties are estimated from the data/simulation ratio in Sect. 8.1.2

The effects of these systematic uncertainty variations on the CST $E_{\text {T}}^{\text {miss}}$ are shown for the $\mathrm{Z} \rightarrow \mu {}\mu$  events in Figs. 24 and 25 for the primary (Sect. 8.1.1) and the cross-check (Sect. 8.1.2) methods, respectively. The uncertainties are larger for the cross-check method, reaching around 50% for $E_\mathrm{T}^{\mathrm {miss,soft}}$ > 60 $\text {GeV}$ in Fig. 25a.

**Fig. 26:**
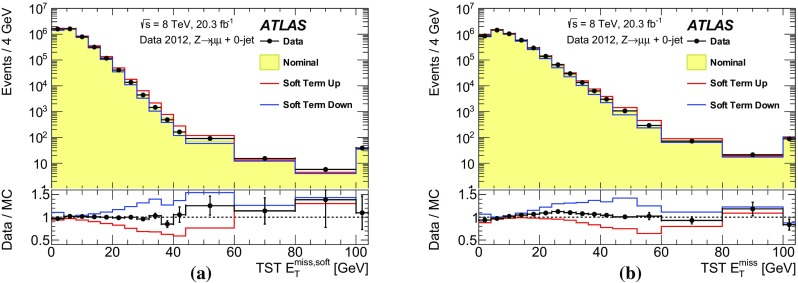
Distributions of **a** $E_\mathrm{T}^{\mathrm {miss,soft}}$ and **b** $E_{\mathrm {T}}^{\mathrm {miss}}$ with the TST algorithm. Data are compared to the nominal simulation distribution as well as those resulting from applying the scale and resolution systematic uncertainties to the $E_\mathrm{T}^{\mathrm {miss,soft}}$ and adding the variations in quadrature, and the *insets* at the *bottom* of the figures show the ratios of the data to the MC predictions. The uncertainties are estimated from the method in Sect. 8.2

The corresponding plots for the TST $E_{\text {T}}^{\text {miss}}$ are shown in Fig. 26 using the $\mathrm{Z} \rightarrow \mu {}\mu$ $+$0-jet control sample, where the uncertainty band is the quadratic sum of the variations with the MC statistical uncertainty. The systematic uncertainty band for the TST is larger in Fig. 26a than the one for the primary CST algorithm. In all the distributions, the systematic uncertainties in the soft term alone cover the disagreement between data and MC simulation.

#### Systematic uncertainties from tracks inside jets

A separate systematic uncertainty is applied to the scalar summed ${p}_{\text {T}}$ of tracks associated with high-${p}_{\text {T}}$  jets in the Track $E_{\text {T}}^{\text {miss}}$ because these tracks are not included in the TST. The fraction of the momentum carried by charged particles within jets was studied in ATLAS [58], and its uncertainty varies from 3 to 5% depending on the jet $\eta$ and ${p}_{\text {T}}$ . These uncertainties affect the azimuthal angle between the Track $E_{\text {T}}^{\text {miss}}$  and the TST $E_{\text {T}}^{\text {miss}}$ , so the modelling is checked with $\mathrm{Z} \rightarrow \mu {}\mu$  events produced with one jet. The azimuthal angle between the Track $E_{\text {T}}^{\text {miss}}$ and the TST $E_{\text {T}}^{\text {miss}}$ directions is well modelled, and the differences between data and MC simulation are within the systematic uncertainties.

## Conclusions

Weakly interacting particles, which leave the ATLAS detector undetected, give rise to a momentum imbalance in the plane transverse to the beamline. An accurate measurement of the missing transverse momentum ($E_{\text {T}}^{\text {miss}}$ ) is thus important in many physics analyses to infer the momentum of these particles. However, additional interactions occurring in a given bunch crossing as well as residual signatures from nearby bunch crossings make it difficult to reconstruct the $E_{\text {T}}^{\text {miss}}$ from the hard-scattering process alone.

The $\vec {E}_{{\mathrm{T}}}^{\mathrm{miss}}$ is computed as the negative vector sum of the reconstructed physics objects including electrons, photons, muons, $\tau$-leptons, and jets. The remaining energy deposits not associated with those high-${p}_{\text {T}}$ physics objects are also considered in the $\vec {E}_{{\mathrm{T}}}^{\mathrm{miss}}$. They collectively form the so-called soft term, which is the $E_{\text {T}}^{\text {miss}}$  component most affected by pileup. The calorimeter and the tracker in the ATLAS detector provide complementary information to the reconstruction of the high-${p}_{\text {T}}$ physics objects as well as the $E_{\text {T}}^{\text {miss}}$ soft term. Charged particles are matched to a particular collision point or vertex, and this information is used to determine which charged particles originated from the hard-scatter collision. Thus tracking information can be used to greatly reduce the pileup dependence of the $E_{\text {T}}^{\text {miss}}$  reconstruction. This has resulted in the development of $E_{\text {T}}^{\text {miss}}$  reconstruction algorithms that combine the information from the tracker and the calorimeter. The performance of these reconstruction algorithms is evaluated using data from 8 $\text {TeV}$ proton–proton collisions collected with the ATLAS detector at the LHC corresponding to an integrated luminosity of 20.3 fb$^{-1}$.

The Calorimeter Soft Term (CST) is computed from the sum of calorimeter topological clusters not associated with any hard object. No distinction can be made between energy contributions from pileup and hard-scatter interactions, which makes the resolution on the $\vec {E}_{{\mathrm{T}}}^{\mathrm{miss}}$ magnitude and direction very dependent on the number of pileup interactions. The pileup-suppressed $E_{\text {T}}^{\text {miss}}$  definitions clearly reduce the dependence on the number of pileup interactions but also introduce a larger under-estimation of the soft term than the CST.

The Track Soft Term (TST) algorithm does not use calorimeter energy deposits in the soft term and uses only the inner detector (ID) tracks. It has stable $E_{\text {T}}^{\text {miss}}$  resolution with respect to the amount of pileup; however, it does not have as good a response as the CST $E_{\text {T}}^{\text {miss}}$, due mainly to missing neutral particles in the soft term. Nevertheless, its response is better than that of the other reconstruction algorithms that aim to combine the tracking and calorimeter information. For large values of $E_\mathrm{T}^\mathrm{miss,True}$, the CST and TST $E_{\text {T}}^{\text {miss}}$  algorithms all perform similarly. This is because contributions from jets dominate the $E_{\text {T}}^{\text {miss}}$  performance, making the differences in soft-term reconstruction less important.

The Extrapolated Jet Area with Filter (EJAF) and Soft-Term Vertex-Fraction (STVF) $E_{\text {T}}^{\text {miss}}$ reconstruction algorithms correct for pileup effects in the CST $E_{\text {T}}^{\text {miss}}$ by utilizing a combination of the ATLAS tracker and calorimeter measurements. Both apply a vertex association to the jets used in the $E_{\text {T}}^{\text {miss}}$ calculation. The EJAF soft-term reconstruction subtracts the pileup contributions to the soft term using a procedure similar to jet area-based pileup corrections, and the EJAF $E_{\text {T}}^{\text {miss}}$  resolution has a reduced dependence on the amount of pileup, relative to the CST algorithm. The STVF reconstruction algorithm uses an event-level correction of the CST, which is the scalar sum of charged-particle ${p}_{\text {T}}$ from the hard-scatter vertex divided by the scalar sum of all charged-particle ${p}_{\text {T}}$ . The STVF correction to the soft term greatly decreases the dependence of the $E_{\text {T}}^{\text {miss}}$ resolution on the amount of pileup but causes the largest under-estimation of all the soft-term algorithms.

Finally, the Track $E_{\text {T}}^{\text {miss}}$  reconstruction uses only the inner detector tracks with the exception of the reconstructed electron objects, which use the calorimeter $E_{\text {T}}$  measurement. The resolutions on the Track $E_{\text {T}}^{\text {miss}}$ magnitude and direction are very stable against pileup, but the limited $|\eta |$ coverage of the tracker degrades the $E_{\text {T}}^{\text {miss}}$ response, as does not accounting for high-${p}_{\text {T}}$ neutral particles, especially in events with many jets.

The different $E_{\text {T}}^{\text {miss}}$  algorithms have their own advantages and disadvantages, which need to be considered in the context of each analysis. For example, removing large backgrounds with low $E_{\text {T}}^{\text {miss}}$ , such as Drell–Yan events, may require the use of more than one $E_{\text {T}}^{\text {miss}}$  definition. The tails of the track and calorimeter $E_{\text {T}}^{\text {miss}}$  distributions remain uncorrelated, and exploiting both definitions in parallel allows one to suppress such backgrounds even under increasing pileup conditions.

The systematic uncertainties in the $E_{\text {T}}^{\text {miss}}$  are estimated with $\mathrm{Z} \rightarrow \mu {}\mu$  events for each reconstruction algorithm, and are found to be small.

## Calculation of EJAF

A jet-level $\eta$-dependent pileup correction of the form

21 $$\begin{aligned} \rho _{\eta }^{\mathrm {med}}(\eta ) = \rho _{\mathrm {evt}}^{\mathrm {med}}\cdot P_{\text {fct}}^\rho (\eta ,N_{\text {PV}},\langle \mu \rangle ), \end{aligned}$$

is used, where the ${N}_{\mathrm {PV}}$ and $\langle \mu \rangle$ are determined from the event properties. This multiplies the median soft-term jet $p_{\text {T}}$-density, $\rho _{\mathrm {evt}}^{\mathrm {med}}$, from Eq. (7) by the functional form, $P_{\text {fct}}^\rho (\eta ,N_{\text {PV}},$ $\langle \mu \rangle$) as defined in Eq. (9), which was fit to the average transverse momentum density. The median transverse momentum density $\rho _{\mathrm {evt}}^{\mathrm {med}}$ is determined from soft-term jets with $|\eta |$ < 2 and then extrapolated to higher $|\eta |$ as discussed in Sect. 4.1.2 using the fitted $P_{\text {fct}}^\rho (\eta ,N_{\text {PV}},$ $\langle \mu \rangle$).

The pileup correction $\rho _{\eta }^{\mathrm {med}}(\eta )$ from Eq. (21) is applied to the transverse momenta of the soft-term jets passing a JVF selection. The pileup-corrected jet ${p}_{\text {T}}$ is labelled $p_{\mathrm {T,}i}^{{\mathrm{filter}{\text {-}}\mathrm{jet}, \mathrm{corr}}}$, and it is computed as

22 $$\begin{aligned} p_{\mathrm {T,}i}^{{\mathrm{filter}{\text {-}}\mathrm{jet}, \mathrm{corr}}}= \left\{ \begin{array}{ll} 0 &{} (p_{\mathrm {T,}i}^{{\mathrm{filter}{\text {-}}\mathrm{jet}}}\le \rho _{\eta }^{\mathrm {med}}({\eta }_{i}^{{\mathrm{filter}{\text {-}}\mathrm{jet}}}) \cdot {A}_{i}^{{\mathrm{filter}{\text {-}}\mathrm{jet}}}) \\ p_{\mathrm {T,}i}^{{\mathrm{filter}{\text {-}}\mathrm{jet}}}- \rho _{\eta }^{\mathrm {med}}({\eta }_{i}^{{\mathrm{filter}{\text {-}}\mathrm{jet}}}) \cdot {A}_{i}^{{\mathrm{filter}{\text {-}}\mathrm{jet}}}&{} (p_{\mathrm {T,}i}^{{\mathrm{filter}{\text {-}}\mathrm{jet}}}> \rho _{\eta }^{\mathrm {med}}({\eta }_{i}^{{\mathrm{filter}{\text {-}}\mathrm{jet}}}) \cdot {A}_{i}^{{\mathrm{filter}{\text {-}}\mathrm{jet}}}). \end{array} \right. \end{aligned}$$

The *x* and *y* components of $p_{\mathrm {T,}i}^{{\mathrm{filter}{\text {-}}\mathrm{jet}, \mathrm{corr}}}$ are used to compute the EJAF soft term using Eq. (10), and only soft-term jets matched to the PV with JVF > 0.25 for $|{\eta }_{i}^{{\mathrm{filter}{\text {-}}\mathrm{jet}}}|~<~2.4$ or jets with $|{\eta }_{i}^{{\mathrm{filter}{\text {-}}\mathrm{jet}}}|$ $\ge$ 2.4 are used. Because of this JVF selection, the label of “filter-jet” is added to the catchment area (${A}_{i}^{{\mathrm{filter}{\text {-}}\mathrm{jet}}}$), to the transverse momentum ($p_{\mathrm {T,}i}^{{\mathrm{filter}{\text {-}}\mathrm{jet}}}$), and to the jet $\eta$ (${\eta }_{i}^{{\mathrm{filter}{\text {-}}\mathrm{jet}}}$) variables.

While all other jets used in this paper use an *R* $=$ 0.4 reconstruction, the larger value of *R* $=$ 0.6 is used to reduce the number of $k_{t}$ soft-term jets with ${p}_{\text {T}}$  $=$ 0 (see Eq. (22)) in the central detector region. While negative energy deposits are possible in the ATLAS calorimeters, their contributions cannot be matched to the soft-term jets by ghost-association. Studies that modify the cluster-to-jet matching to include negative-${p}_{\text {T}}$  clusters indicate no change in the $E_{\text {T}}^{\text {miss}}$  performance, so negative-${p}_{\text {T}}$  clusters are excluded from the soft-term jets. Finally, only filter-jets with $p_{\mathrm {T,}i}^{{\mathrm{filter}{\text {-}}\mathrm{jet}}}$ larger than the pileup correction contribute to the EJAF soft term.

## Footnotes

1 ATLAS uses a right-handed coordinate system with its origin at the nominal interaction point (IP) in the centre of the detector and the *z*-axis along the beam pipe. The *x*-axis points from the IP to the centre of the LHC ring, and the *y*-axis points upward. Cylindrical coordinates $(r,\phi )$ are used in the transverse plane, $\phi$ being the azimuthal angle around the beam pipe. The pseudorapidity is defined in terms of the polar angle $\theta$ as $\eta =-\ln \tan (\theta /2)$.

2 ATLAS defines stable particles as those having a mean lifetime $>0.3\times 10^{-10}$ s.

3 The track reconstruction for electrons and for muons does not strictly follow these definitions. For example, a Gaussian Sum Filter [9] algorithm is used for electrons to improve the measurements of its track parameters, which can be degraded due to Bremsstrahlung losses.

4 The $\text {arctan}$ function returns values from $[-\pi ,+\pi ]$ and uses the sign of both coordinates to determine the quadrant.

5 The EM scale is the basic signal scale for the ATLAS calorimeters. It accounts correctly for the energy deposited by EM showers in the calorimeter, but it does not consider energy losses in the un-instrumented material.

6 The forward ATLAS calorimeters are less granular than those in the central region, which leads to fewer clusters being reconstructed.

7 As defined in Sect. 4.1.3, the CST $E_{\text {T}}^{\text {miss}}$  does not apply a JVF requirement on the jets like the TST, EJAF, and STVF $E_{\text {T}}^{\text {miss}}$ . However, large $\Sigma E_{\mathrm {T}}^\mathrm{jets}$ tends to come from hard-scatter jets and not from pileup.

8 For the TST and Track $E_{\text {T}}^{\text {miss}}$, mismeasured high-$p_{\text {T}}$  tracks with ${p}_{\text {T}}$  > 120 (200) $\text {GeV}$ are removed using the track quality requirements in high (low) $|\eta |$ as defined in Sect. 4.1.2.
